# Extracellular Vesicles Derived Human-miRNAs Modulate the Immune System in Type 1 Diabetes

**DOI:** 10.3389/fcell.2020.00202

**Published:** 2020-03-31

**Authors:** Tine Tesovnik, Jernej Kovač, Katka Pohar, Samo Hudoklin, Klemen Dovč, Nataša Bratina, Katarina Trebušak Podkrajšek, Maruša Debeljak, Peter Veranič, Emanuele Bosi, Lorenzo Piemonti, Alojz Ihan, Tadej Battelino

**Affiliations:** ^1^Department of Pediatric Endocrinology, Diabetes and Metabolic Diseases, University Children’s Hospital, University Medical Centre Ljubljana, Ljubljana, Slovenia; ^2^Clinical Institute of Special Laboratory Diagnostics, University Medical Centre Ljubljana, University Children’s Hospital, Ljubljana, Slovenia; ^3^Faculty of Medicine, University of Ljubljana, Chair of Paediatrics, Ljubljana, Slovenia; ^4^Faculty of Medicine, Institute of Microbiology and Immunology, University of Ljubljana, Ljubljana, Slovenia; ^5^Faculty of Medicine, Institute of Cell Biology, University of Ljubljana, Ljubljana, Slovenia; ^6^Faculty of Medicine, Institute of Biochemistry, University of Ljubljana, Ljubljana, Slovenia; ^7^IRCCS Ospedale San Raffaele, San Raffaele Diabetes Research Institute, Milan, Italy; ^8^Vita-Salute San Raffaele University, Milan, Italy

**Keywords:** type 1 diabetes, extracellular vesicles, miRNA, immune modulating activity, autoimmune disease, TLR7/8

## Abstract

Extracellular vesicles with their molecular cargo can modulate target cell response and may affect the pathogenesis of diseases. The extracellular vesicles containing micro-RNAs (miRNAs), which are often studied as disease biomarkers, but rarely as mediators of the disease development. The role of extracellular vesicles derived miRNAs in type 1 diabetes is currently not well established. We observed a fraction of blood plasma extracellular vesicles positive for membrane proteins potentially associated with insulin-producing beta-cells and identified differentially expressed extracellular vesicles derived miRNAs in individuals with type 1 diabetes. These differentially expressed extracellular vesicles derived human miRNAs in participants with type 1 diabetes and participants with Langerhans islets beta-cells destruction showed the ability to activate TLR7/8 signaling cascade and increase activation as well as cytotoxicity of the effector blood immune cells with cytokine and chemokine release. Our results illustrate extracellular vesicles derived human miRNAs as modulators of the immune system in type 1 diabetes autoimmunity, providing potentially new insight into the pathogenesis of the disease, and novel molecular targets for intervention and type 1 diabetes prevention.

## Introduction

Type 1 diabetes (T1D) incidence is increasing worldwide (Katsarou et al., 2017), affecting both pediatric (Patterson et al., 2019) and adult populations (Salam et al., 2018; Thomas et al., 2018). T1D is an autoimmune disease involving environmental and genetic factors triggering selective destruction of insulin-producing pancreatic beta-cells (Katsarou et al., 2017). The etiology of the disease remains unknown, nonetheless, the most widely accepted theory attributes the T1D onset to the environmental stress-factors affecting the presentation of self-antigens leading to overt autoimmunity (Christoffersson et al., 2016; Paschou et al., 2017). The disease demands lifelong applications of exogenous insulin (Battelino et al., 2017; Katsarou et al., 2017) or treatment with Langerhans islet transplantation (Piemonti and Pileggi, 2000). The individuals with T1D are at risk of acute hyper/hypoglycemic periods and persistent glucose variability (Ceriello and Kilpatrick, 2013), that reduce the quality of life and lead to the development of diabetes complications, accelerated cell senescence (Tesovnik et al., 2018), and early mortality (Katsarou et al., 2017). The direct insight into cell pathogenesis is impeded due to the inaccessibility of the Langerhans islets. The increasing disease prevalence, associated healthcare costs and cumulative negative impact on the quality of life for individuals with T1D and their families clearly demonstrate the need to improve the disease management at earliest stages of its development, with the ultimate goal of preventing or even reversing the disease before the critical level of beta-cell destruction. Analysis of extracellular vesicles obtained from body fluids allows us to probe physiological conditions in distant organs, disease monitoring, and define novel biomarkers (Armstrong and Wildman, 2018), which could also provide a better insight into the pathogenesis of T1D.

Extracellular vesicles (EVs) are small spherical structures encased with the cell membrane, released from cells into extracellular space, carrying parental-cell-specific molecules. There are three main types of EVs; exosomes, microvesicles and apoptotic bodies, which differ by cell-release mechanism, their origin and dimensions (Yáñez-Mó et al., 2015; van Niel et al., 2018). However, it is difficult to differentiate between different types of EVs. Using parental cell-specific molecules, such as proteins, lipids, glycosides, and nucleic acids, EVs are able to transfer the information of parental cells’ to the neighboring cells (Yáñez-Mó et al., 2015) or even to cells in other organs of the organism, where a target cell response can be triggered (Hoshino et al., 2015). With cell-to-cell communication, EVs are involved in physiological as well as potential pathologically cellular processes (Yuana et al., 2013; Hoshino et al., 2015; Tan et al., 2016). While the immunomodulatory role of EVs has been characterized in cancer (Becker et al., 2016; Kamerkar et al., 2017), a few studies also indicate the EVs role in autoimmunity (Tan et al., 2016; Turpin et al., 2016; Salvi et al., 2018; Pluta et al., 2019). Although initial studies on animal models and cell cultures suggest the potential involvement of beta-cell stress released EVs in the disruption of the immune system (Cianciaruso et al., 2017; Freeman et al., 2018), the role of beta-cells’ EVs RNA in autoimmune diabetes has not been defined.

We aimed to determine the presence of insulin-producing beta-cell derived EVs in human blood plasma and demonstrate their potential as a proxy for immune system modulation. Using deep next-generation sequencing, we analyzed human blood plasma EVs’ small RNAs, with the focus on micro RNAs (miRNAs), differentially expressed in children with new-onset T1D, and identified miRNAs potentially involved in the autoimmune pathogenesis of T1D. Furthermore, we demonstrated that differentially expressed miRNAs packed in vesicles could be internalized by immune system cells, preferentially monocytes and granulocytes, via the endolysosomal pathway, consequently activating TLR7/8 response of innate immunity and upregulating activation, transition, and cytotoxicity of the adaptive immune system. To the best of our knowledge, this is the first study investigating human-EVs-derived differentially expressed miRNAs as the potential pathogenic modulators of beta-cell-specific autoimmunity in T1D. Our results demonstrated the potential pathologic immunomodulatory effect of human miRNAs in T1D etiology and a novel potential molecular target for disease treatment and prevention.

## Materials and Methods

The study was designed in three steps: (1) isolating plasma and Langerhans islet medium EVs fraction and TEM EVs imaging (2) comparative EVs plasma miRNA Next-generation sequencing of T1D participants, transplantation individuals and healthy control samples and (3) *in vitro* differentially expressed vesicle miRNA effect study on the human whole blood immune cells. The workflow of our study is presented in Supplementary Figure S1.

### Participants With T1D Onset; T1D 10-Years Duration; Healthy Controls; Langerhans Islet Transplantation Patients

Three blood plasma samples of healthy individuals were collected for EVs miRNA profile characterization and comparison to total plasma and depleted EVs plasma profile.

Ten T1D onset, ten T1D 10-years duration and ten healthy controls blood samples were collected to evaluate EVs miRNA in T1D. Blood plasma of ten new-onset T1D participants (nT1D) was collected at the time of the first hospital visit after the disease onset, typically on day 5 or 6. All newly diagnosed children with T1D were positive for at least one of T1D related antibodies (GAD65, ZnT8, or IA-2), participants were in a pre-pubertal state with no other diagnosed autoimmune diseases or other disorders at the T1D onset (T1D age onset: 6.49 ± 2.57 years, 5 females). Participants with 10-year T1D duration (10yT1D) were examined at regular follow-up medical examinations; participants were not diagnosed for other autoimmune disorders nor diabetic complications (age: 17.76 ± 2.35 years, duration of the disease: 13.03 ± 1.95 years, 5 females). Ten healthy 5-years-old control (HC) individuals’ blood samples were collected during the national systematic check-up examination (age: 5.33 ± 0.33 years, 4 females). Healthy controls did not have T1D or type 2 diabetes family history and were not diagnosed with T1D at the time of this study, nor did they have detectable T1D related antibodies. The characteristics of the participants are listed in Table 1. For characterization of the EVs small non-coding RNA profile, participants’ blood was collected into 10 mL K-EDTA tubes, blood plasma was isolated with 3,000×*g* for 10 min centrifugation and stored at −80°C before further processing, no longer than 6 months. T1D and 10yT1D were clinically characterized by University Children’s Hospital, Department of Pediatric Endocrinology, Diabetes and Metabolic Diseases.

**TABLE 1:** Characteristics of cohorts included in EVs small RNA sequencing.

**Group**	**Individual**	**T1D age onset**	**Age at the examination**	**T1D duration**	**BMI**	**BMI**	**T1D antibodies**	**0**’ **C-peptide**	**6**’ **C-peptide**	**Delta C-peptide**
		**[years]**	**[years]**	**[years]**	**[kg/m^2^]**	**Z-score**		**[nmol/L]**	**[nmol/L]**	**[nmol/L]**
nT1D	nT1D_1	6−10	6−10	0	18.9	0.99	GAD65, IA2, ZnT8	0.13	0.61	0.48
	nT1D_2	6−10	6−10	0	13.5	−2.45	ZnT8	0.03	0.07	0.04
	nT1D_3	1−5	6−10	0	15.6	−0.46	GAD65, ZnT8	0	0.05	0.05
	nT1D_4	1−5	1−5	0	13.1	−2.84	GAD65, IA2, ZnT8	0.07	0.16	0.08
	nT1D_5	1−5	6−10	0	12.9	−3.43	GAD65	0	0.16	0.16
	nT1D_6	6−10	6−10	0	13.6	−1.73	GAD65, IA2, ZnT8	0.04	0.07	0.03
	nT1D_7	11−15	6−10	0	16.5	−0.5	GAD65, IA2, ZnT8	0.04	0.11	0.07
	nT1D_8	6−10	6−10	0	13.3	−1.93	GAD65, IA2, ZnT8	0.1	0.2	0.1
	nT1D_9	1−5	1−5	0	17.3	1.24	GAD65	0.1	0.2	0.1
	nT1D_10	6−10	6−10	0	17.6	1.13	GAD65, IA2, ZnT8	0.2	0.4	0.2

10yT1D	10yT1D_1	1−5	16−20	11−15	25.7	1.3	−	−	−	−
	10yT1D_2	1−5	16−20	16−20	20.8	−0.64	−	−	−	−
	10yT1D_3	1−5	16−20	16−20	20.1	−0.42	−	−	−	−
	10yT1D_4	1−5	11−15	11−15	25.1	1.16	−	−	−	−
	10yT1D_5	1−5	16−20	11−15	22.0	0.43	−	−	−	−
	10yT1D_6	1−5	16−20	11−15	18.4	−1.09	−	−	−	−
	10yT1D_7	6−10	16−20	11−15	26.0	0.99	−	−	−	−
	10yT1D_8	11−15	21−25	11−15	24.7	−	−	−	−	−
	10yT1D_9	1−5	16−20	11−15	20.4	0.16	−	−	−	−
	10yT1D_10	11−15	21−25	6−10	22.1	−	−	−	−	−

HC	HC_1	/	1−5	/	17.5	1.22	−	−	−	−
	HC_2	/	1−5	/	15.5	−0.94	−	−	−	−
	HC_3	/	1−5	/	15.7	0.13	−	−	−	−
	HC_4	/	1−5	/	15.1	−0.26	−	−	−	−
	HC_5	/	1−5	/	15.0	−0.8	−	−	−	−
	HC_6	/	1−5	/	16.7	−0.3	−	−	−	−
	HC_7	/	1−5	/	14.1	−1.23	−	−	−	−
	HC_8	/	1−5	/	17.4	1.22	−	−	−	−
	HC_9	/	1−5	/	15.7	0.22	−	−	−	−
	HC_10	/	1−5	/	14.4	−1.86	−	−	−	−

*Characteristics of nT1D, 10yT1D, and HC participants: for every participant are reported the age as a range at T1D onset, age as a range at the examination when sample for EVs isolation was obtained, T1D duration and Z-score and BMI (BMI only for participants older than 21 years). nT1D participants were at the onset of the disease positive at least for one of T1D associated antibodies (GAD65, Glutamic Acid Decarboxylase; IA-2, Insulinoma Antigen 2; ZnT8, Zinc Transporter 8) [nT1D, new-onset T1D, *n* = 10; 10yT1D, 10 years duration T1D, *n* = 10; HC, healthy controls, *n* = 10; − : data below the limit of detection; /: no data].*

To specify small non-coding RNA EVs profile and signals during active beta-cells destruction in Langerhans islet transplantation stress *in vivo*, where some beta-cells are damaged, miRNA profiles of human Langerhans islet transplantation patients’ (TX) EVs plasma fraction were assessed. Blood samples were obtained prior and after the transplantation procedure from two adult Langerhans islet transplantation recipients (55–50 and 55–60 years old males), both with long-standing T1D. Blood samples were obtained in K-EDTA tubes before the transplantation and after 1, 6, and 24 h. Blood plasma was isolated with 3,000×*g* for 10 min centrifugation and stored at −80°C before further processing, not longer than 4 months. The transplantation plasma samples were provided by the San Raffaele Diabetes Research Institute, IRCCS Ospedale San Raffaele, Milan, Italy. Signed written informed consent was obtained before the study.

### Langerhans Islets’ EVs

Transmission electron microscopy (TEM) was used to assess the beta-cells EVs in plasma samples, and plasma EVs were compared to Langerhans islets medium EVs, which were used as a beta-cells’ EVs positive control. The Langerhans medium samples of 3 adult donors (51–55 year-old female; 41–45 year-old male; 46–50 year-old male) were provided by the San Raffaele Diabetes Research Institute, IRCCS Ospedale San Raffaele, Milan, Italy. The medium where Langerhans islets were cultured at sufficient purity for transplantation (Layer I; >80% purity) was used for TEM characterization. Raw culture medium consisted of CMRL medium without phenol red and with HAS, Hepes, Di-pep-Gln (CORNING, 99-784-CM), to which Nicotinamide (0.01 M), Glutamine (2 mM), and Penicillin/Streptomycin (100U/L) were added. After the Langerhans islets medium collection, the medium was centrifuged 10 min at 3,000×*g* to remove cell debris and stored at −80°C before further EVs characterization.

### Plasma EVs and Langerhans Medium EVs Isolation

Blood plasma and Langerhans medium were thawed and centrifuged for 30 min at 10,000×*g* to remove cell debris. EVs were isolated by the modified protocol based on previously published PEG isolation procedures (Rider et al., 2016; Ludwig et al., 2018). 1 mL of pre-centrifuged plasma was resuspended with 400 μL of PEG-8000 (0.4 g PEG/1mL 1x PBS) (Sigma Aldrich, 81268 and 806544) and incubated for 30 min at 4°C. EVs fraction was collected after 10 min centrifugation at 10,000×*g*.

For isolation Langerhans medium EVs, the medium was centrifuged 30 min at 10,000×*g* to remove cell debris and a higher concentration of precipitation reagent PBS-PEG 8000 was used (500 μL medium, 1 mL 0.5 g PEG/1mL 1x PBS) (isolation based on: Rider et al., 2016) to precipitate EVs. Langerhans islet EVs precipitate fraction was isolated with 10 min centrifugation at 10,000×*g*, after 30 min pre-incubation of PEG-Langerhans medium at 4°C.

Isolated EVs fractions were further used for TEM imaging or miRNA isolation.

### TEM Imaging

Pellets of PEG isolated EVs from plasma samples or Langerhans medium were prepared for immuno-electron microscopy following the modified Tokuyasu technique (Hudoklin et al., 2011, 2012). Briefly, samples were fixed with 4% formaldehyde in 0.1 M phosphate buffer, pH 7.2, for 2.5 h at room temperature and washed with PBS 3 × 5 min and with 0.15% glycine/PBS 2x5min. Pellets were embedded in 12% gelatine, cut into 0.5 × 0.5 × 0.5 mm blocks, cryoprotected with 2.3 M sucrose for 24 h at 4°C. The blocks were then mounted on specimen holders and frozen in liquid nitrogen. Ultrathin cryosections were cut with EM-FCS UCT (Leica) at −120°C, thickness 60 nm, and retrieved with 1:1 mixture of 2.3 M sucrose and 2% methylcellulose on Au grids.

For immunolabeling, sections were incubated on 2% gelatine, washed with 0.15% glycine/PBS 5 × 2 min and blocked with 1% BSA-c (Aurion; 900.022).

Primarily antibodies for Langerhans cells’ specific EVs markers were selected based on The Human Protein Atlas database (^1^ Protein Atlas version 16) with characterized high expression in endocrine pancreas and negligible expression in other tissues. We used validated Human protein atlas antibodies against anti-human-GAD-2 (GAD-65 | Sigma Aldrich, AMAb91048-100UL, HP044637-100UL), anti-human-DGCR-2 (Sigma Aldrich, HPA000873-100UL) and anti-human-PTPRN (IA-2 | Sigma Aldrich, APA007179-100UL). Intra-vesicular EVs INS detection was performed on the plasma samples treated with proteinase K 30min 37°C to degrade plasma free INS. The samples were labeled with anti-human-INS (Abbexa, abx019113) antibody in the combination with selected human protein atlas antibodies (DGCR2, GAD2, PTPRN). Incubation with primary antibodies was performed overnight at 4°C and was followed by incubation with the secondary goat-anti-mouse or goat-anti-rabbit polyclonal antibodies conjugated with 6 and 18 nm colloidal gold (Jackson ImmunoResearch, goat anti rabbit 6 nm 111-195-144, goat anti-rabbit 18 nm 111-215-144, goat anti-mouse 6 nm 115-195-146, and goat anti-mouse 18 nm 115-215-146). Negative controls were done by omitting the primary antibodies, by incubation in rabbit serum, or by using inadequate primary antibodies. After secondary gold-labeled antibody conjugation, sections were stained with methylcellulose/uranyl acetate (Sigma-Aldrich, M-6385; Polysciences 21447), dried, and examined with CM100 transmission electron microscope (Philips) running at 80 kV.

### Extracellular Vesicles’ miRNA Profile Specification

Plasma EVs PEG enriched precipitate of three adult participants were suspended in 200 μL PBS and resuspended in 1mL of Qiazol reagent (Qiagen). Plasma RNA and EVs depleted plasma (PEG-EVs depleted plasma) RNA samples were isolated from 1 mL of blood plasma and 1.3 mL depleted EVs plasma with the addition of the prescribed volume of Qiazol, following the manufacturer’s protocol. RNA was further isolated using RNeasy columns (Qiagen), RWT and RPE buffers (Qiagen) and eluted in nuclease-free water. The RNA profiles were assessed using RNA Pico 6000 kit on Bioanalyzer 2100 (Agilent, 5067-1514) and samples were prepared for sequencing. EVs, blood plasma and EVs depleted blood plasma small RNA libraries were prepared for NGS sequencing using NEBNext Small RNA Library Prep Set for Illumina (New England Biolabs, E7300S) without the size selection step. NGS libraries were checked for QC using DNA 1000 kit on Bioanalyzer 2100 (Agilent, 5067-1505) and quantified using NEBNext Library Quant Kit for Illumina (New England Biolabs, E7630) before being sequenced on MiSeq Illumina sequencer with at least 5M reads per sample. The sample type miRNA profiles specificity was evaluated using bioinformatics analysis.

### T1D Extracellular Vesicles’ RNA Characterization

Type 1 diabetes, 10yT1D, and HC as well as TX plasma enriched EVs precipitated fractions were resuspended in 200 μL PBS and dissolved in 1 mL Qiazol (Qiagen, 79306). After chloroform-Qiazol centrifugation and aqueous layer phase separation, RNA was isolated following a manufacture’s procedure using RNeasy Mini Spin Columns (Qiagen), cleaned with RWT, RPE buffers (Qiagen) and eluted in nuclease free water. Isolated RNA size distribution profile and concentration was assessed on Bioanalyzer 2100 using Bioanalyzer 6000 Pico kit (Agilent, 5067–1514) and stored at −80°C before further processing. Small non-coding RNA EVs libraries were prepared using NEBNext Multiplex Small RNA Library Prep Set for Illumina (New England Biolabs, E7300S) following standard protocol without size selection step. NGS libraries were checked for QC using DNA 1000 kit on Bioanalyzer 2100 (Agilent, 5067-1505) and quantified using NEBNext Library Quant Kit for Illumina (New England Biolabs, E7630). Small RNA libraries were pooled in equimolar ratio. EVs plasma miRNA samples were sequenced with 150 M raw sequencing reads for nT1D, 10yT1D, HC, and TX samples on Illumina Hiseq.

### miRNAs Bioinformatics Analysis

Raw sequences data were trimmed for the adapter sequence using Cutadapt 1.14 (Martin, 2011) (trimming parameters: *Q* ≥ 20, minimal sequence length 15 nucleotides) and further analyzed using sRNAtoolbox (Rueda et al., 2015), a collection of tools for small RNA analysis. sRNAbench module with genome mapping mode was used for alignment and annotation on hg38 human genome reference and sRNAde integrated tool was implemented for differential expression analysis.

For EVs plasma samples miRNA characterization, EVs miRNA profiles were compared to total plasma and EVs depleted plasma. miRNAs of which all the samples of at least one group exceeded 10 miRNAs read counts were compared using the differential expression analysis.

Low sequenced reads per miRNA species were excluded from the differential expression comparison analysis. In the same way miRNA of nT1D, 10yT1D, and HC individuals with a minimum of 10 read counts were compared with the differential expression analysis. Similarly, TX participants’ miRNAs profiles after 1, 6, and 24 h after the transplantation were compared to the samples obtained before the transplantation procedure, where miRNAs in at least one group exceeded 100 miRNAs read counts. edgeR results with FDR < 0.05 were considered as statistically significant.

On the basis of differential expression analysis, eight significantly differentially expressed miRNA with FDR < 0.05 were selected for *in vitro* testing. hsa-miR-122-5p, hsa-miR-192-5p, hsa-miR-193b-5p, hsa-miR-185-5p, hsa-miR-195-3p, and hsa-miR-455-5p were selected based on the results of nT1D-HC, 10yT1D-HC and 10yT1D-nT1D differential expression analysis, while hsa-miR-375-3p and hsa-miR-129-5p were the most significantly overexpressed miRNAs in Langerhans islets transplantation patients.

### miRNA *in vitro* Whole Blood Early and Transient Activation and Degranulation

The immunomodulatory effect of selected differentially expressed EVs miRNAs was assessed on whole blood samples of ten healthy participants and ten newly diagnosed children with T1D (participants’ characteristics in Table 2). Whole blood samples of participants were collected into 10 mL Na-heparin tubes. For miRNAs vesicle derived stimulation, the blood of participants was diluted by RPMI in 1:1 ratio and aliquoted to 0.5 mL in sterile round-bottom polystyrene tubes. HPLC purified miRNAs (IDT) hsa-miR-122-5p, hsa-miR-192-5p, hsa-miR-193b-5p, hsa-miR-185-5p, hsa-miR-195-3p, hsa-miR-455-5p, hsa-miR-375-3p, and hsa-miR-129-5p mixture with DOTAP (N-[1- (2,3-Dioleoyloxy)propyl]-N,N,N-trimethylammonium methyl-sulfate; Sigma Aldrich, 11202375001) reagent were used to generate EVs like vesicular species that were added to whole blood samples to be internalized via endolysosomal pathway.

**TABLE 2:** Characteristics of new-onset T1D (nT1D) participants and healthy controls (HC) included in DOTAP-miRNA stimulation test.

**nT1D**	**Age [years]**	**T1D antibodies**	**HC**	**Age [years]**	**T1D antibodies**
P1	1−5	GAD65, IA2	N1	6−10	−
P2	11−15	GAD65, IA2, ZnT8	N2	11−15	−
P3	11−15	GAD65	N3	11−15	−
P4	11−15	GAD65, ZnT8	N4	6−10	−
P5	11−15	GAD65, IA2, ZnT8	N5	6−10	−
P6	6−10	GAD65, IA2, ZnT8	N7	6−10	−
P7	6−10	GAD65, IA2, ZnT8	N8	6−10	−
P8	6−10	IA2, ZnT8	N9	6−10	−
P9	6−10	GAD65	N10	11−15	−
P10	6−10	GAD65, IA2, ZnT8	N11	6−10	−

*For every nT1D and HC participant are reported the age as a range and the presence of T1D related antibodies (−: below the limit of detection). The cohorts did not statistically differ in age (Wilcoxon parametric *t*-test: *p* = 0.8256) and sex (Chi-square test: *p* = 0.6531).*

DOTAP/miRNAs complex was prepared by combining 7.5 μL DOTAP/mL with miRNAs final reaction concentration 0.5 μM. The optimal miRNA concentration was determined from the results of the titration DOTAP/miRNAs complex (Supplementary Figure S2). 0.5 μM ssRNA40/LyoVec (Inviogen, tlrl-lrna40), 10 μg/mL Poly (I:C) HWC (Invivogen, tlrl-pic), 2.5 μg/mL PHA with 200 IU/mL IL2 (PHA: Sigma Aldrich, L9017-1MG; IL2: ROCHE, 11011456001) were used as positive controls. Blood alone, 0.5 μM RNA41 (Beignon et al., 2005, 1) (HPLC purified, IDT) with 7.5 μL DOTAP/mL and DOTAP (7.5 μL DOTAP/mL) alone were used as negative controls. To specify the effect of extracellularly delivered miRNAs in non-vesicular form, miRNAs were also added to the cell suspension without DOTAP. Altogether 24 conditions were tested. The stimulation response was monitored after 21 h overnight incubation at 37°C, 5% CO_2_. After 20 min labeling using mouse-anti-human antibodies for early and transient activation (CD69) on CD4+ and CD8+ T-cells (BD Biosciencesanti-human-CD69 FITC, 347823; BD Pharmigen: anti-human-CD3 PE, 555340; BD Biosciences: anti-human-CD8 PerCP-Cy 5.5, 341050; BD Pharmigen: anti-human-CD4 APC, 555349), and cytotoxicity/degranulation (CD107a) of CD56+ NK cells and CD8+ T-cells (BD Biosciences: anti-human-CD3 FITC 345762; BD Pharmigen:anti-human-CD107a PE, 555801; BD Biosciences: anti-human-CD8 PerCP-Cy 5.5, 341050; BD Pharmigen:anti-human-CD56 APC, 555518), erythrocytes were lysed using BD FACS Lysing Solution (BD Biosciences; 349202), and lymphocytes quantified by BD FACSCanto II System (BD Biosciences). The flow cytometry results were analyzed using BD FACSDiva^TM^ Software (BD Biosciences).

### Extracellular Vesicle Clearance and Accumulation in Immune Cells

Next, the importance of vesicle delivery and miRNA intracellular accumulation in the cells of the immune system was investigated. miRNA DOTAP vesicle complex internalization and accumulation in the immune cells were assessed on three *in vitro* adult whole blood samples (blood collected in heparinized tubes). Samples were diluted by RPMI in 1:1 ratio transfected *in vitro* with 0.5 μM fluorescent-labeled hsa-miR-375-3p-FAM alone (HPLC purified hsa-miR-375-3p-FAM, IDT), DOTAP/hsa-miR-375-3p-FAM complex, and DOTAP alone.

After 2 h of stimulation, the initial wash step with Phagotest Reagent A (Phagotest; Glycotope Biotechnology, 341060) was used to remove the surplus of miRNA in cell medium, and CD14+ monocytes were labeled for 20 min with anti-human-CD14 PerCP-Cy 5.5 antibody (BD Bioscience; ref: 550787). The fluorescence of potentially attached miRNAs on the cytoplasmic membrane was quenched with the Phagotest Reagent B and after the wash steps, erythrocytes were lysed using Phagotest reagent C. After the wash, cells populations were analyzed using BD FACSCanto II System (BD Biosciences). FSC, SSC, and anti-CD14 parameters were used to specify the lymphocytes, monocytes and granulocytes populations. hsa-miR-375-3p-FAM (FITC absorbance/emission spectrum) signal was used to evaluate miRNA internalization and the quantitative determination of the up-taken vesicles. hsa-miR-375-3p-FAM and DOTAP/hsa-miR-375-3p-FAM were compared to cells-autofluorescence as a result of the stimulation with DOTAP alone. Additionally, internalized and accumulated vesicle DOTAP/miRNA-FAM in the whole blood cells were analyzed after 5 and 30 min, 1, 2, 4, and 6 h from the beginning of the transfection. The results were compared to the sample with the unlabeled hsa-miR-375-3p with DOTAP (DOTAP/miRNA). For every sample at the investigated time of the experiment, the vesicle clearance and accumulation were calculated with the relative median fluorescence intensity ratio of DOTAP delivered hsa-miR-375-3p-FAM vs. DOTAP delivered hsa-miR-375-3p.

Micro-RNAs delivered with DOTAP vesicles’ intracellular accumulation as well as intracellular compartmentalization was characterized using fluorescent microscopy. Whole blood samples from adult healthy individuals were obtained and erythrocytes were lysed with RBC Lysis Buffer (Cell Signaling technology, 46232) and after wash step, leukocytes were resuspended in RPMI-1640 Medium (Sigma Aldrich, R8758). The cells were transfected with DOTAP/hsa-miR-375-3p-FAM (0.5 μM miRNA), where DOTAP vesicles were stained using CellBrite Orange Cytoplasmic Membrane Dye (Biotium, 30022) before the transfection. After 2 h of incubation at 37°C 5% CO2, cells were washed with PBS and stained with Lysosomal Staining Reagent (Abcam, ab176828). The samples were fixated using 4% formaldehyde (Sigma Aldrich, F8775), and cytoplasmic membrane was stained using CellBrite Blue Cytoplasmic Membrane Dye (Biotium, 30024). The cells were imaged using Olympus fluorescence microscopy system (Olympus Life Science BX61). Pictures were processed using ImageJ software (Schneider et al., 2012).

### Vesicle-miRNAs TLR7/8 Activation, Transfection Reagent Specificity, and miRNAs Structure Dependent Activation

To confirm the vesicle derived miRNAs induction of TLR7/8 signaling the Chloroquine (CQ) (Invivogen, tlrl-chq) was used. The stimulation DOTAP/miRNA effect was inhibited with the increasing concentrations of CQ, after ssRNA40/LyoVec and hsa-miR-455-5p DOTAP stimulation. After the CQ titration, the 20 μM CQ inhibitory concentration was identified as optimal (Supplementary Figure S3) and used to inhibit DOTAP hsa-miR-122-5p, hsa-miR-455-5p, hsa-miR-375-3p, hsa-miR-129-5p TLR7/8 activation effect. miRNA TLR7/8 inhibition was assessed on six whole blood adult healthy controls collected in Na-heparin tubes by the aforementioned procedure (labeling protocol: miRNA *in vitro* whole blood early and transient activation and degranulation) with the addition of 20 μM CQ to the reaction tubes before 21h overnight incubation.

Furthermore, the DOTAP transfection reagent specificity was characterized and compared to RNAiMAX Lipofectamine transfection reagent. The parallel sample transfection experiments were performed on three adult donors on the whole blood samples. Transfection reagent specificity was evaluated with transfection of hsa-miR-122-5p, hsa-miR-129-5p, hsa-miR-375-3p, hsa-miR-455-5p, and hsa-miR-193b-5p, including reactions with CQ inhibitor. RNAiMAX Lipofectamine transfection was performed in 0.5 mL 1:1 RPMI diluted whole blood cells following the manufacturer’s protocol with the final 0.5 μM miRNA concentration and 3.5 μL RNAiMAX Lipofectamine/mL. The 20 μM CQ concentration was used in inhibition reactions. For both transfection reagents, DOTAP and RNAiMAX Lipofectamine alone were used as negative controls and ssRNA-40/LyoVec as a positive immune activation control.

The immunomodulatory effect was assessed using flow cytometry for assessing early activation marker CD69 on CD4+ and CD8+ T-cells and expression of CD107a+ cytotoxicity marker on CD8+ T-cells and CD56+ NK-cells (described in the “Mirna *in vitro* Whole Blood Early and Transient Activation and Degranulation” section).

Due to different stimulation responses of studied miRNAs in our “miRNA *in vitro* whole blood early and transient activation and degranulation” results, the miRNAs structures were correlated to the immune-stimulation effect. To predict studied miRNAs secondary structure miRNAs sequences were analyzed with RNAfold web server (Lorenz et al., 2011).

### Cytokine/Chemokine Profile Detection

DOTAP/miRNA immune system activation was additionally evaluated by assessing cytokine and chemokine inflammatory profiles. The whole blood samples of three healthy adult donors were used for DOTAP-miRNA stimulation reactions (hsa-miR-122-5p, hsa-miR-129-5p, hsa-miR-375-3p, hsa-miR-455-5p, and hsa-miR-193b-5p), DOTAP alone as a negative control and ssRNA-40/LyoVec as a positive immune activation control. Samples were incubated 21 h overnight (37°C, 5% CO_2_). After 5 min centrifugation at 3000×*g*, samples’ supernatants were transferred to cryotubes and stored at −80°C. Supernatants were diluted 25-times and the cytokine/chemokine profile was detected using LEGENDplex Human Inflammation Panel multi-analyte flow assay kit (BioLegend, 740118) using BD FACSCanto II System (BD Biosciences) and data were analyzed using LEGENDplex v8.0 software.

### Statistics

Multiple comparisons for different miRNAs stimulation experiments were calculated following the rules of estimation statistics to better represent the effect size of the comparison, its distribution, and to compensate for the flaws of the null-hypothesis significance testing (Cumming, 2014; Claridge-Chang and Assam, 2016). The differences in selected parameters between control and stimulation experiments were calculated using paired and unpaired estimation test implemented in Python DABEST package (Ho et al., 2019). Cytokine and chemokine release was evaluated with Kruskal-Wallis one-way ANOVA test. The results (corresponding *p*-Values) of multiple comparisons were corrected for the false discovery rate (FDR) using two-stage Benjamini, Krieger, and Yekutieli (TSBKY) procedure implemented in Python module StatsModels 0.9.0 (Benjamini et al., 2006). Stimulation DOTAP/miRNA flow cytometry results and cytokine release results were considered significant when FDR *q*-Value was lower than 0.05 for paired mean difference analysis and 0.1 for nT1D-HC group unpaired difference analysis.

Titration curve figures and boxplots of DOTAP/miRNAs, QC titration as well as QC inhibitory response were created in R using ggplot2 (Wickham, 2016).

## Results

### Beta-Cells From Langerhans Islets Release Extracellular Vesicles to Blood Plasma

Due to a low amount of pancreatic beta-cells (Weir and Bonner-Weir, 2013) in proportion to the whole human body, the presence of pancreatic insulin-producing Langerhans islets beta-cells’ EVs was investigated in blood plasma. PEG-based EVs precipitation was used to isolate EVs fraction in blood plasma, and TEM was used to morphologically identify and characterize EVs in the isolated fractions. TEM immunogold labeling on cryo-ultrathin sections confirmed the presence of EVs positive endocrine-pancreatic-cell-membrane-specific proteins GAD2 (GAD65), PTPRN (IA-2) and DGCR2 with a diameter of isolated EVs up to 500 nm (Supplementary Figure S4). EVs isolated from human Langerhans islet medium, collected during pre-transplantation storage and maintenance of Langerhans islets, were used as a positive control in the immunogold labeling experiment.

**FIGURE 1:**
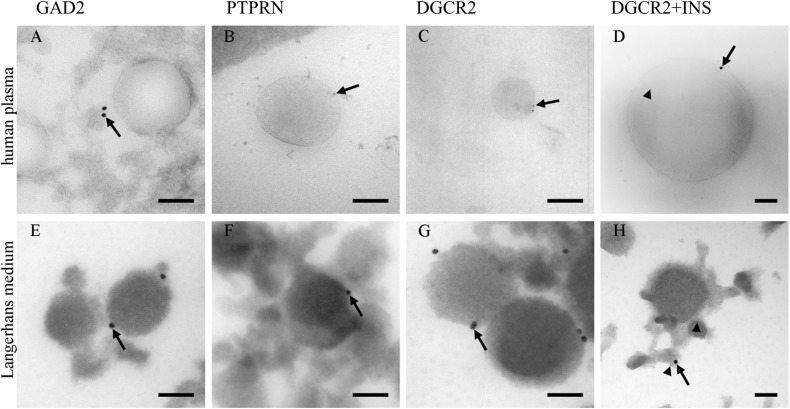
Immunolabeling of EVs. Immunolabeling of EVs isolated from human blood plasma **(A–D)** and the medium of cultured human Langerhans islets **(E–H)**. Antibodies against GAD2 **(A,E)**, PTPRN **(B,F),** and PTPRN **(C,G)** proteins labeled limiting EVs (arrows). Antibodies against insulin were also presented in the lumen of vesicles (arrow-head; **D,H**). INS, insulin; GAD2, Glutamate decarboxylase 2 (GAD65); DGCR2, DiGeorge Syndrome Critical Region Gene 2; PTPRN, Protein tyrosine phosphatase receptor type N. Bars: 100 nm.

The majority of EVs in *ex vivo* cultured Langerhans islets’ medium was positive for the expression of DGCR2 (Figure 1G), estimated to be 10/100 EVs. Conversely, immunogenic T1D associated proteins GAD2 and PTPRN were detected (Figures 1E,F) in a smaller proportion (estimated at 1/100 EVs). EVs in blood plasma were also positive for endocrine-pancreatic cell-membrane specific proteins DGCR2 (estimated at 1/10.000 EVs), GAD2 and PTPRN (Figures 1A–C) (estimated at 1/100.000 EVs), confirming the existence of EVs originating from Langerhans islets in the circulating blood. Furthermore, we also detected DGCR2 positive EVs for intra-vesicular insulin (INS) (Figures 1D,H).

TEM imaging confirmed our hypothesis of beta-cell-released EVs in the blood plasma and potential beta-cells’ communication with other tissues. This led us to further investigate the role of EVs and their RNA cargo in the new-onset T1D plasma EVs.

### Extracellular Vesicle, Blood Plasma, EVs Depleted Plasma miRNA Profiles

EVs, the whole blood plasma, and EVs depleted plasma miRNA profiles (Supplementary Figure S5) were compared using NGS differential expression analysis (Supplementary Figure S6). The analysis revealed different miRNA profiles among EVs, plasma and EVs depleted plasma samples, where the type source of samples (EVs, the whole blood plasma, and EVs depleted plasma) showed greater similarity compared to the samples of the investigated individuals. The sample type clustering is shown in Supplementary Figure S6. The results provided evidence of EVs miRNA profile specificity, which allowed our further EVs miRNA analysis of T1D individuals.

### Extracellular Vesicles’ miRNAs in Type 1 Diabetes and During Intensive Beta-Cell Destruction

The isolated EVs fractions from pediatric pre-pubertal individuals’ plasma samples with nT1D were analyzed using next-generation sequencing, which allows profiling whole EVs small-RNA transcriptome. All nT1D individuals were positive for T1D related autoantibodies and had a negative family history for other autoimmune diseases. To specify small RNAs associated with T1D pathogenesis nT1D EVs miRNA profile was compared to the profiles of the cohort with 10yT1D and negative T1D autoantibodies, and to a cohort of HC (Characteristics of participants: Table 1).

**FIGURE 2:**
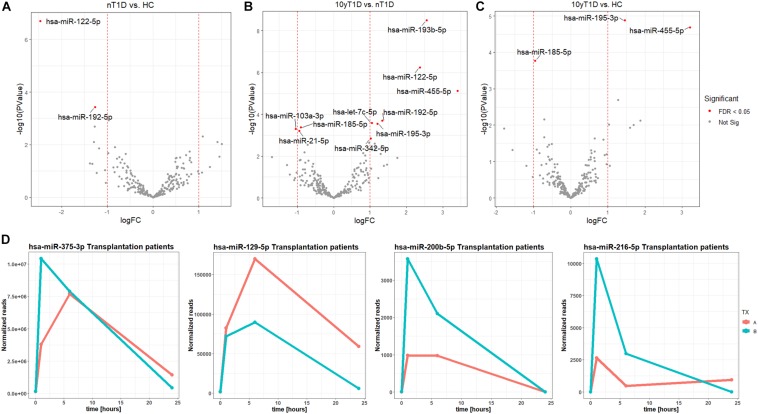
Differentially expressed EVs miRNAs between nT1D, 10yT1D, HC, and Islet transplantation participants. Differential expression Volcano plots of panel **(A)** nT1D and HC, **(B)** 10yT1D and HC, and **(C)** 10yT1D and nT1D (nT1D, new-onset T1D participants, *N* = 10; 10yT1D – 10 years duration T1D participants, *N* = 10; HC, healthy controls, *N* = 10; red dots represent differentially expressed miRNAs with FDR < 0.05). **(D)** Chronological presentation of differentially expressed hsa-miR-375-3p, hsa-miR-129-5p, hsa-miR-200b-5p, and hsa-miR-216-5p in Langerhans islet transplantation participants [TX; **(A,B**) anonymized individuals] before (0 h), and 1, 6, and 24 h after the transplantation.

A higher expression of hsa-miR-122-5p and hsa-miR-192-5p were identified in nT1D cohort when compared to HC (Figure 2A). Differential expression analysis of nT1D versus 10yT1D showed ten potentially differentially expressed miRNAs, most of them with higher expression in nT1D: hsa-miR-193b-5p, hsa-miR-122-5p, and hsa-miR-445-5p showing the most significant difference between compared groups (Figure 2B). Furthermore, hsa-miR-195-3p and hsa-miR-455-5p expression were lower, and hsa-miR-185-5p expression was higher in the 10yT1D-cohort, compared to HC (Figure 2C). Detailed results of differential expression analysis of plasma EVs miRNAs are presented in Table 3.

**TABLE 3:** sRNA toolbox differential expression of nT1D, 10yT1D, HC EVs miRNAs sequencing results.

**nT1D− HC**	**Average nT1D reads**	**Average HC reads**	**logFC**	**logCPM**	***P*-value**	**FDR**
**hsa-miR-122-5p**	**4,832,438.59**	**873,511.46**	−**2.46785**	**16.72597**	**2.01E-07**	**4.96E-05**
**hsa-miR-192-5p**	**41,257.96**	**17,091.76**	−**1.27135**	**10.25227**	**0.000378**	**0.046676**
hsa-miR-378c	143.27	48.72	−1.55396	2.239277	0.000898	0.073921
hsa-miR-193b-5p	3,945.46	1,625.99	−1.27892	6.671317	0.002011	0.124193

**10yT1D - HC**	**Average 10yT1D reads**	**Average HC reads**	**logFC**	**logCPM**	***P*-value**	**FDR**

**hsa-miR-195-3p**	**1,636.58**	**4,503.83**	**1.460336**	**7.339928**	**1.31E-05**	**0.002516**
**hsa-miR-455-5p**	**61.75**	**573.41**	**3.213685**	**4.404186**	**2.04E-05**	**0.002516**
**hsa-miR-185-5p**	**107,325.25**	**55,217.37**	−**0.95879**	**11.81067**	**0.000168**	**0.013854**
hsa-miR-193b-5p	670.36	1,625.99	1.277958	6.671317	0.002032	0.125479

**10yT1D – nT1D**	**Average 10yT1D reads**	**Average nT1D reads**	**logFC**	**logCPM**	***P*-value**	**FDR**

**hsa-miR-193b-5p**	**670.36**	**3,945.46**	**2.556865**	**6.671317**	**3.12E-09**	**7.72E-07**
**hsa-miR-122-5p**	**937,326.45**	**4,832,438.59**	**2.366128**	**16.72597**	**5.62E-07**	**6.95E-05**
**hsa-miR-455-5p**	**61.75**	**657.31**	**3.410471**	**4.404186**	**7.36E-06**	**0.000606**
**hsa-let-7c-5p**	**19,030.00**	**39,414.73**	**1.050441**	**10.49258**	**0.000263**	**0.011701**
**hsa-miR-192-5p**	**16,394.18**	**41,257.96**	**1.331473**	**10.25227**	**0.000201**	**0.011701**
**hsa-miR-195-3p**	**1,636.58**	**3,783.13**	**1.208751**	**7.339928**	**0.000284**	**0.011701**
**hsa-miR-185-5p**	**107,325.25**	**57,606.27**	−**0.89769**	**11.81067**	**0.000421**	**0.01486**
**hsa-miR-103a-3p**	**7,893.16**	**3,831.02**	−**1.04276**	**8.13815**	**0.000496**	**0.015299**
**hsa-miR-21-5p**	**194,384.87**	**100,930.19**	−**0.94556**	**12.83508**	**0.000621**	**0.017045**
**hsa-miR-342-5p**	**3,763.81**	**7,595.65**	**1.012932**	**8.101386**	**0.001444**	**0.035672**
hsa-miR-193a-5p	1,193.08	2,612.78	1.130626	6.479336	0.002455	0.050524
hsa-miR-99a-5p	6,818.56	13,390.50	0.973615	8.923166	0.002289	0.050524
hsa-miR-26a-5p	53,554.47	32,617.91	−0.71534	11.0815	0.002887	0.054844
hsa-miR-100-5p	1,199.68	2,641.43	1.138557	6.625715	0.003799	0.063369
hsa-miR-483-5p	396.41	1,040.34	1.391449	4.955521	0.003848	0.063369
hsa-miR-20a-5p	2,076.58	1,190.35	−0.80257	6.350269	0.006577	0.101525
hsa-miR-125a-5p	8,138.86	14,308.18	0.813912	9.126002	0.007022	0.102028
hsa-miR-150-3p	1,580.15	3,857.16	1.28739	6.946875	0.00793	0.108823
hsa-miR-1246	517.56	945.83	0.869583	5.027435	0.009174	0.11926
hsa-miR-652-3p	106.61	32.75	−1.69822	1.944765	0.011289	0.139417
hsa-miR-365b-5p	83.10	280.48	1.753845	3.458699	0.012046	0.14168

*Differential expression edgeR results of EVs miRNAs results reported as average normalized read counts per miRNAs in groups of participants, log fold-change (logFC), log counts per million (logCPM), *P*-value and FDR for differentially expressed miRNAs. Only miRNAs with FDR lower than 0.15 are reported in this table, miRNAs with FDR < 0.05 were considered as statistically significant differentially expressed miRNAs and are indicated in bold [nT1D, new-onset T1D, *n* = 10; 10yT1D, 10 years duration T1D, *n* = 10; HC, healthy controls, *n* = 10].*

The EVs signals from insulin-producing beta-cells affected by autoimmunity might not be successfully assessed in a cohort of nT1D individuals, because of variably in the preserved beta-cells mass. To evaluate and specify the EVs related to beta-cell destruction (transplantation procedure exposes beta-cells in Langerhans islets to elevated stress characterized by a stress-signature released miRNA), two TX recipients’ blood plasma samples were taken before and one, six and 24 h after the transplantation. EVs from these plasma samples were sequenced (average 125M+ reads/sample), and miRNAs content was analyzed. Generated miRNA profiles demonstrated a typical trend for specific miRNA species, with low levels before the transplantation, increased 1 h after the procedure, followed by a steady temporal decline in their levels. miRNA species following this expression trend included hsa-miR-375-3p, hsa-miR-129-5p, miR-200b-5p, and hsa-miR-216b-5p (Figure 2D and Table 4).

**TABLE 4:** sRNA toolbox differential expression analysis results of transplantation patients EVs miRNAs.

**0**−**1 h**	**TX-A-0h**	**TX-B-0h**	**TX-A-1h**	**TX-B-1h**	**logFC**	**logCPM**	***P*-value**	**FDR**
**hsa-miR-375-3p**	**136,975.67**	**127,121.52**	**4,368,840.55**	**9,970,659.54**	**5.76278**	**15.87156**	**1.69E-10**	**1.02E-07**
**hsa-miR-129-5p**	**1,702.33**	**1,582.07**	**94,994.24**	**68,848.56**	**5.640418**	**9.850739**	**2.65E-07**	**7.99E-05**
**hsa-miR-216b-5p**	**3.69**	**6.78**	**3,046.05**	**9,894.10**	**10.23924**	**5.071745**	**7.66E-06**	**0.00154**
**hsa-miR-200b-5p**	**3.69**	**6.78**	**1,129.48**	**3,414.97**	**8.729792**	**3.849213**	**4.09E-05**	**0.006166**
**hsa-miR-148a-3p**	**9,998,029.89**	**9,061,974.78**	**35,735,485.55**	**98,568,812.39**	**2.816885**	**18.73319**	**0.000112**	**0.013546**
**hsa-miR-122-5p**	**1,216,369.54**	**1,502,879.92**	**15,503,699.54**	**4,756,837.30**	**2.897392**	**16.40263**	**0.000305**	**0.030693**
**hsa-miR-4433b-3p**	**8,357.09**	**14,649.83**	**200.71**	**780.87**	−**4.55107**	**5.750177**	**0.000395**	**0.034015**
hsa-miR-125a-5p	1,070.08	5,163.14	18,243.83	41,876.67	3.269816	7.781954	0.001069	0.080548
hsa-miR-200a-3p	2,311.44	718.73	10,964.20	17,189.51	3.215605	7.092002	0.001227	0.082218
hsa-miR-216a-3p	1.84	3.39	665.09	1,619.78	8.703054	2.902087	0.001504	0.090667

**0-6 h**	**TX-A-0h**	**TX-B-0h**	**TX-A-6h**	**TX-B-6h**	**logFC**	**logCPM**	***P*-value**	**FDR**

**hsa-miR-375-3p**	**136,975.67**	**127,121.52**	**6,901,633.47**	**6,653,568.65**	**5.681633**	**15.87156**	**2.6E-10**	**1.57E-07**
**hsa-miR-129-5p**	**1,702.33**	**1,582.07**	**152,258.22**	**75,867.93**	**6.117951**	**9.850739**	**4.73E-08**	**1.42E-05**
**hsa-miR-200b-5p**	**3.69**	**6.78**	**878.90**	**1,776.87**	**7.959885**	**3.849213**	**0.000105**	**0.018083**
**hsa-miR-216b-5p**	**3.69**	**6.78**	**424.64**	**2,510.18**	**8.103809**	**5.071745**	**0.00012**	**0.018083**

***0-24h***	**TX-A-0h**	**TX-B-0h**	**TX-A-24h**	**TX-B-24h**	**logFC**	**logCPM**	***P*-value**	**FDR**

**hsa-miR-129-5p**	**1,702.33**	**1,582.07**	**62,388.85**	**6,028.34**	**4.380553**	**9.850739**	**2.25E-05**	**0.013545**
**hsa-miR-493-5p**	**695.18**	**1,636.30**	**4.52**	**2.99**	**−8.22825**	**3.977484**	**7.36E-05**	**0.0222**
hsa-miR-375-3p	136,975.67	127,121.52	1,503,963.49	444,589.80	2.883261	15.87156	0.000264	0.05316
hsa-miR-216b-5p	3.69	6.78	982.52	5.98	6.532697	5.071745	0.001061	0.099431
hsa-miR-4433b-3p	8,357.09	14,649.83	1,012.36	344.99	**−**4.08287	5.750177	0.001154	0.099431
hsa-miR-486-3p	81,442.82	34,780.37	306,362.18	502,742.48	2.799422	11.54946	0.000708	0.099431
hsa-miR-654-3p	666.48	1,058.85	2.71	1.99	**−**8.44209	3.083302	0.000844	0.099431

*Langerhans islets transplantation individuals (TX represents a transplantation participant, letters A and B represent an individual A and B) edgeR differential expression EVs miRNAs results before (0 h) and after 1, 6, and 24 h after the transplantation procedure. Differentially expressed miRNAs are reported as miRNAs normalized read count, log fold-change (logFC) and log counts per million (logCPM), the difference of expression is reported with *P*-value and FDR. Only miRNAs with FDR lower than 0.10 are reported, miRNAs with FDR < 0.05 are considered as significant and are indicated in bold.*

Based on our nT1D miRNA and Langerhans islet transplantation individuals’ miRNA profiling results, eight differentially expressed miRNAs were selected in individuals with nT1D with FDR < 0.05 for further *in vitro* miRNAs synthetic vesicles immunomodulation testing.

### Extracellular Vesicles’ miRNA Immunomodulatory Role in Type 1 Diabetes

**FIGURE 3:**
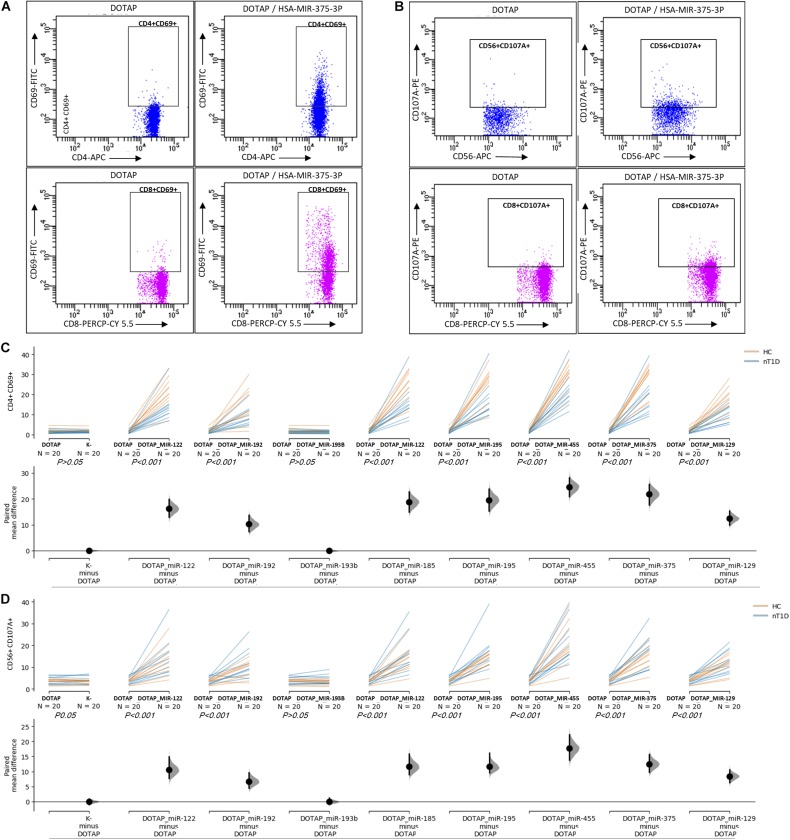
The stimulation effect of endolysosomal delivered miRNAs. **(A)** Representative flow cytometry stimulation effect of DOTAP as a negative control (left scatter plots) and DOTAP/hsa-miR375-3p (right scatter plots) on the upregulated expression of CD69 early activation marker effect on CD4+ (top scatter plots) and CD8+ T-cells (bottom scatter plots). **(B)** The effect of stimulation on the upregulated expression of CD107a marker (cytotoxicity/degranulation) on CD56+ NK cells (top scatter plots) and CD8+ T-cells (bottom scatter plots) after the stimulation with DOTAP – a negative control (left scatter plots) and DOTAP/hsa-miR375-3p (right scatter plots). Paired data analysis plots with effect size are presented for panel **(C)** CD4+ CD69+ results, and **(D)** CD56+ CD107a+. Whole blood samples were treated with different DOTAP/miRNAs and compared to DOTAP and K- (blood sample alone) as negative controls [the observed effects were assessed 21 h after the stimulation; HC and nT1D *N* = 20; *P*-values represent a paired Hedges’ g mean difference analysis significance: *P* < 0.001 significant difference].

**FIGURE 4:**
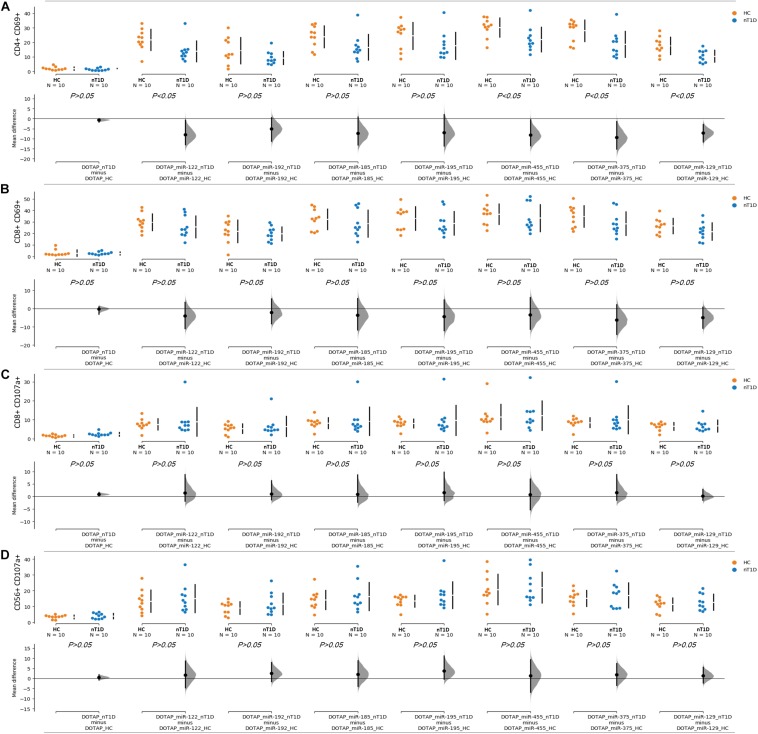
Differences in the stimulation effect between compared HC and nT1D participants. The graphs show the differences in stimulation effect on the upregulated expression of early activation marker CD69 on **(A)** CD4+ T-cells and **(B)** CD8+ T-cells in blood samples of nT1D and HC. The differences between HC and nT1D were significant in the stimulation experiments with DOTAP/hsa-miR-122-5p, DOTAP/hsa-miR-455-5p, DOTAP/hsa-miR-375-3p, and DOTAP/hsa-miR-129-5p (*P* < 0.05). The stimulation effect was not observed in expression of CD107a cytotoxicity/degranulation marker on **(C)** CD8+ T-cells and **(D)** CD56+ NK-cells [the observed effects were assessed 21 h after the stimulation; HC *N* = 10; nT1D *N* = 10; *P-*values represent an unpaired Hedges’ g mean difference analysis significance: *P* < 0.05 significant difference].

The effect of differentially expressed miRNAs in T1D was investigated on the pediatric whole blood specimens from nT1D and HC participants (Characteristics of participants: Table 2), with the aim to preserve as much as possible blood immune cells sub-populations and intricate cells’ interactions in *in vitro* testing. To specify the effect of differentially expressed miRNA and mimic the EVs miRNAs delivery to the target cells of the immune system without other immunomodulatory EVs components, individual miRNA species were packed with N-[1-(2,3-Dioleoyloxy)propyl]-N,N,N-trimethylammonium methyl-sulfate (DOTAP) synthetic vesicles, entering cells via the endolysosomal pathway. Eight selected miRNAs differentially expressed in people with diabetes and transplantation individuals (hsa-miR-122-5p, hsa-miR-192-5p, hsa-miR-193b-5p, hsa-miR-185-5p, hsa-miR-195-3p, hsa-miR-455-5p, hsa-miR-375-3p, and hsa-miR-129-5p) were tested for their potential effect on early and transient activation as well as cytotoxicity of the effector immune cells. All tested miRNAs, except hsa-miR-193b-5p, substantially induced CD69 early and transient activation of CD4C and CD8C T-cells in whole blood samples, as well as CD107a degranulation (cytotoxicity) of CD56+ NK and CD8+ T-cells in all tested samples (Figures 3A–D, 4 and Tables 5A,B) (*p* < 0.0001). Interestingly, the stimulation with “naked” miRNAs (not integrated into vesicles to evade endolysosomal path), and empty DOTAP vesicles did not induce any detectable activation or cytotoxic degranulation (Tables 5A,B). The DOTAP/miRNAs vesicles delivery was generally associated with lower early transient activation of CD4+ T-cell in nT1D compared to HC (Figures 4A,B). Compelling differences in the expression of CD4+ CD69+ between nT1D and HC were observed at stimulation with hsa-miR-122-5p (overexpressed in nT1D), hsa-miR-455-5p (with higher expression in nT1D and HC), hsa-miR-375-3p, and hsa-miR-129-5p (elevated in Langerhans islet transplantation and associated with beta-cell stress and Langerhans islets’ damage) (*p* < 0.05) (Figure 4A and Table 6A). Tested DOTAP/miRNAs complexes strongly induced CD8+ T-cells proliferation, NK cell (CD56+) and T cell (CD8+) CD107a+ cytotoxic degranulation, however, the difference in activity between nT1D and HC were not observed (Figures 4B–D and Tables 6A,B).

**TABLE 5A:** Vesicle delivered miRNA effect on CD69+ T-cell activation (paired mean difference analysis).

**CD4+CD69+**	**Control**	**Difference**	***p*-Value paired_students_*t*-test**	***q*-Value**	**Discovery**
K-	DOTAP	2.6264E-02	8.6365E-01	5.8758E-01	No
**DOTAP/miR-122**	**DOTAP**	**2.7464E+00**	**1.6806E-08**	**4.4134E-08**	**Yes**
**DOTAP/miR-192**	**DOTAP**	**1.8868E+00**	**4.9719E-06**	**8.1600E-06**	**Yes**
DOTAP/miR-193b	DOTAP	−7.4805E-02	5.1846E-01	5.7924E-01	No
**DOTAP/miR-185**	**DOTAP**	**2.9176E+00**	**8.4219E-09**	**2.7645E-08**	**Yes**
**DOTAP/miR-195**	**DOTAP**	**2.7709E+00**	**3.4213E-08**	**6.4174E-08**	**Yes**
**DOTAP/miR-455**	**DOTAP**	**3.9724E+00**	**5.3356E-11**	**7.0056E-10**	**Yes**
**DOTAP/miR-375**	**DOTAP**	**3.2153E+00**	**2.2949E-09**	**1.5066E-08**	**Yes**
**DOTAP/miR-129**	**DOTAP**	**2.7410E+00**	**2.2487E-08**	**4.9210E-08**	**Yes**
DOTAP_RNA41	DOTAP	4.2537E-02	7.6026E-01	5.8758E-01	No
miR-455	DOTAP	1.5354E-02	8.9017E-01	5.8758E-01	No
miR-122	K-	−9.7474E-02	6.6254E-01	5.8758E-01	No
miR-192	K-	−1.5876E-01	4.7228E-01	5.7924E-01	No
miR-193b	K-	−1.1766E-01	5.7350E-01	5.7924E-01	No
miR-185	K-	−1.7129E-02	9.3976E-01	5.8758E-01	No
miR-195	K-	−5.9610E-02	7.8414E-01	5.8758E-01	No
miR-455	K-	−1.1846E-02	9.3739E-01	5.8758E-01	No
miR-375	K-	3.6059E-17	1.0000E+00	5.9682E-01	No
miR-129	K-	9.6813E-02	5.3791E-01	5.7924E-01	No
RNA41	K-	−2.5097E-02	8.8383E-01	5.8758E-01	No
**TLR7-8**	**DOTAP**	**3.0293E+00**	**6.3610E-09**	**2.7645E-08**	**Yes**
**TLR3**	**DOTAP**	**1.0111E+00**	**5.3732E-04**	**7.8389E-04**	**Yes**

**CD8+CD69+**	**Control**	**Difference**	***p*-Value paired_students_*t*-test**	***q*-Value**	**Discovery**

K-	DOTAP	−3.5588E-02	7.3354E-01	5.0607E-01	No
**DOTAP/miR-122**	**DOTAP**	**3.9153E+00**	**9.7819E-12**	**6.4218E-11**	**Yes**
**DOTAP/miR-192**	**DOTAP**	**3.0016E+00**	**1.4703E-09**	**2.4132E-09**	**Yes**
DOTAP/miR-193b	DOTAP	−1.0218E-01	2.7066E-01	2.5384E-01	No
**DOTAP/miR-185**	**DOTAP**	**3.6261E+00**	**9.7638E-11**	**2.5640E-10**	**Yes**
**DOTAP/miR-195**	**DOTAP**	**3.7437E+00**	**2.0622E-10**	**4.5127E-10**	**Yes**
**DOTAP/miR-455**	**DOTAP**	**4.2105E+00**	**8.4335E-12**	**6.4218E-11**	**Yes**
**DOTAP/miR-375**	**DOTAP**	**3.8933E+00**	**5.2785E-11**	**1.7327E-10**	**Yes**
**DOTAP/miR-129**	**DOTAP**	**3.8765E+00**	**2.1802E-11**	**9.5421E-11**	**Yes**
DOTAP_RNA41	DOTAP	2.0586E-02	8.4795E-01	5.0607E-01	No
miR-455	DOTAP	−1.6476E-01	1.2326E-01	1.6184E-01	No
miR-122	K-	−4.3292E-02	7.0370E-01	5.0607E-01	No
miR-192	K-	−1.0950E-01	3.1443E-01	2.7523E-01	No
miR-193b	K-	−2.9184E-02	8.0635E-01	5.0607E-01	No
miR-185	K-	−2.3014E-02	8.4403E-01	5.0607E-01	No
miR-195	K-	−1.7466E-01	1.8840E-01	2.0614E-01	No
miR-455	K-	−1.4118E-01	1.5360E-01	1.8335E-01	No
miR-375	K-	−9.4143E-02	3.3667E-01	2.7628E-01	No
miR-129	K-	−8.4734E-02	4.4671E-01	3.4502E-01	No
RNA41	K-	−1.0715E-01	2.6647E-01	2.5384E-01	No
**TLR7-8**	**DOTAP**	**3.3806E+00**	**3.3197E-10**	**6.2268E-10**	**Yes**
**TLR3**	**DOTAP**	**1.6381E+00**	**4.2134E-06**	**6.1470E-06**	**Yes**

*Hedges’ g correction with 5000 re-samplings and 95% confidence interval was used to predict the effect of miRNA stimulation. Stimulation results with FDR *q*-Value < 0.05 were considered as significant. DOTAP, DOTAP synthetic vesicles; DOTAP/miRNA, miRNA implicated with DOTAP synthetic vesicles; K-, samples with only blood cells; miR, investigated miRNA alone.*

**TABLE 5B:** Vesicle delivered miRNA effect on CD107a+ degranulation (paired mean difference analysis).

**CD8+CD107a+**	**Control**	**Difference**	***p*-Value paired_students_*t*-test**	***q*-value**	**Discovery**
K-	DOTAP	−1.0618E-01	2.9950E-01	1.9105E-01	No
**DOTAP/miR-122**	**DOTAP**	**1.5409E+00**	**1.4560E-05**	**2.5210E-05**	**Yes**
**DOTAP/miR-192**	**DOTAP**	**1.3375E+00**	**4.7945E-05**	**7.2636E-05**	**Yes**
DOTAP/miR-193b	DOTAP	9.6877E-03	9.5480E-01	5.3277E-01	No
**DOTAP/miR-185**	**DOTAP**	**1.6789E+00**	**5.2593E-06**	**1.4867E-05**	**Yes**
**DOTAP/miR-195**	**DOTAP**	**1.6455E+00**	**7.3601E-06**	**1.4867E-05**	**Yes**
**DOTAP/miR-455**	**DOTAP**	**1.9170E+00**	**2.2720E-06**	**9.1790E-06**	**Yes**
**DOTAP/miR-375**	**DOTAP**	**1.8501E+00**	**1.3130E-06**	**7.9570E-06**	**Yes**
**DOTAP/miR-129**	**DOTAP**	**2.4048E+00**	**1.7697E-09**	**2.1448E-08**	**Yes**
DOTAP_RNA41	DOTAP	−5.3420E-03	9.6707E-01	5.3277E-01	No
miR-455	DOTAP	−2.0158E-01	2.7230E-01	1.8335E-01	No
miR-122	K-	−2.1979E-01	8.4525E-02	6.4027E-02	No
miR-192	K-	−2.3455E-01	1.6733E-01	1.1930E-01	No
miR-193b	K-	−3.1049E-01	3.3047E-02	2.8610E-02	No
miR-185	K-	−4.0712E-01	2.5528E-02	2.3800E-02	No
miR-195	K-	−4.4367E-01	2.0979E-02	2.2280E-02	No
miR-455	K-	−8.9528E-02	4.6490E-01	2.8173E-01	No
**miR-375**	**K-**	**−4.3032E-01**	**2.9780E-04**	**3.6094E-04**	**Yes**
miR-129	K-	−2.8434E-01	8.4272E-02	6.4027E-02	No
RNA41	K-	−2.3236E-01	2.2060E-02	2.2280E-02	No
**TLR7-8**	**DOTAP**	**1.5650E+00**	**7.3086E-06**	**1.4867E-05**	**Yes**
**TLR3**	**DOTAP**	**9.4161E-01**	**8.9379E-05**	**1.2036E-04**	**Yes**

**CD56+CD107a+**	**Control**	**Difference**	***p*-Value paired_students_*t*-test**	***q*-Value**	**Discovery**

K-	DOTAP	−1.3256E-02	9.0796E-01	5.7902E-01	No
**DOTAP/miR-122**	**DOTAP**	**1.8273E+00**	**2.4102E-06**	**4.5210E-06**	**Yes**
**DOTAP/miR-192**	**DOTAP**	**1.6059E+00**	**9.7302E-06**	**1.5970E-05**	**Yes**
DOTAP/miR-193b	DOTAP	−1.4613E-02	9.0412E-01	5.7902E-01	No
**DOTAP/miR-185**	**DOTAP**	**2.1148E+00**	**3.7265E-07**	**8.1548E-07**	**Yes**
**DOTAP/miR-195**	**DOTAP**	**2.3678E+00**	**5.7913E-08**	**1.5208E-07**	**Yes**
**DOTAP/miR-455**	**DOTAP**	**2.5599E+00**	**3.8286E-08**	**1.2567E-07**	**Yes**
**DOTAP/miR-375**	**DOTAP**	**2.5780E+00**	**5.9181E-09**	**2.5901E-08**	**Yes**
**DOTAP/miR-129**	**DOTAP**	**2.4245E+00**	**5.4724E-09**	**2.5901E-08**	**Yes**
DOTAP_RNA41	DOTAP	2.0922E-01	2.0392E-01	2.4340E-01	No
miR-455	DOTAP	−6.3502E-03	9.7018E-01	5.7902E-01	No
miR-122	K-	1.5036E-01	5.4555E-01	4.7754E-01	No
miR-192	K-	1.1245E-01	7.0847E-01	5.7902E-01	No
miR-193b	K-	1.7812E-01	5.0498E-01	4.7360E-01	No
miR-185	K-	−1.5432E-02	9.5770E-01	5.7902E-01	No
miR-195	K-	2.3277E-01	3.8008E-01	3.8388E-01	No
miR-455	K-	6.2975E-03	9.6463E-01	5.7902E-01	No
miR-375	K-	−1.5547E-02	9.4458E-01	5.7902E-01	No
miR-129	K-	3.4333E-01	3.0670E-01	3.3558E-01	No
RNA41	K-	3.5283E-01	2.0151E-01	2.4340E-01	No
**TLR7-8**	**DOTAP**	**2.7014E+00**	**1.9012E-09**	**2.4963E-08**	**Yes**
**TLR3**	**DOTAP**	**9.2900E-01**	**4.3701E-04**	**6.3755E-04**	**Yes**

*Hedges’ g correction was used with 5000 re-samplings and 95% confidence interval to predict the effect of miRNA stimulation. Stimulation results with FDR *q*-Value < 0.05 were considered as significant. DOTAP, DOTAP synthetic vesicles; DOTAP/miRNA, miRNA implicated with DOTAP synthetic vesicles; K-, samples with only blood cells; miR, investigated miRNA alone.*

**TABLE 6A:** New-onset T1D vs. healthy controls EVs-miRNA *in vitro* CD69+ T-cell activation (unpaired mean difference analysis).

**CD4+CD69+**	**Control**	**Test**	**Difference**	***p*-Value mann_whitney**	***q*-Value**	**Discovery?**
0	K-_HC	K-_nT1D	−0.44444	0.2712	0.2106	No
1	DOTAP_HC	DOTAP_nT1D	−0.59812	0.1397	0.1454	No
**2**	**DOTAP/miR-122_HC**	**DOTAP/miR-122_nT1D**	**−1.04901**	**0.0190**	**0.0530**	**Yes**
3	DOTAP/miR-192_HC	DOTAP/miR-192_nT1D	−0.68356	0.2123	0.1751	No
4	DOTAP/miR-193b_HC	DOTAP/miR-193b_nT1D	−0.49906	0.3631	0.2663	No
**5**	**DOTAP/miR-185_HC**	**DOTAP/miR-185_nT1D**	**−0.85531**	**0.0587**	**0.0968**	**Yes**
6	DOTAP/miR-195_HC	DOTAP/miR-195_nT1D	−0.7087	0.1405	0.1454	No
**7**	**DOTAP/miR-455_HC**	**DOTAP/miR-455_nT1D**	**−1.02069**	**0.0257**	**0.0530**	**Yes**
**8**	**DOTAP/miR-375_HC**	**DOTAP/miR-375_nT1D**	**−1.08936**	**0.0257**	**0.0530**	**Yes**
**9**	**DOTAP/miR-129_HC**	**DOTAP/miR-129_nT1D**	**−1.32454**	**0.0091**	**0.0530**	**Yes**
**10**	**DOTAP_RNA41_HC**	**DOTAP_RNA41_nT1D**	**−1.15444**	**0.0021**	**0.0272**	**Yes**
**11**	**miR-122_HC**	**miR-122_nT1D**	**−1.24898**	**0.0222**	**0.0530**	**Yes**
12	miR-192_HC	miR-192_nT1D	−0.77725	0.1690	0.1593	No
13	miR-193b_HC	miR-193b_nT1D	−0.85842	0.1992	0.1751	No
**14**	**miR-185_HC**	**miR-185_nT1D**	**−1.23485**	**0.0281**	**0.0530**	**Yes**
**15**	**miR-195_HC**	**miR-195_nT1D**	**−1.01682**	**0.0660**	**0.0968**	**Yes**
16	miR-455_HC	miR-455_nT1D	−0.76955	0.1297	0.1454	No
17	miR-375_HC	miR-375_nT1D	−0.63762	0.5219	0.3626	No
18	miR-129_HC	miR-129_nT1D	−0.75303	0.1432	0.1454	No

**CD8+CD69+**	**Control**	**Test**	**Difference**	***p*-Value mann_whitney**	***q*-Value**	**Discovery?**

0	K-_HC	K-_nT1D	0.111526	0.3073	0.7285	No
1	DOTAP_HC	DOTAP_nT1D	−0.13007	0.4043	0.7285	No
2	DOTAP/miR-122_HC	DOTAP/miR-122_nT1D	−0.45179	0.2565	0.7285	No
3	DOTAP/miR-192_HC	DOTAP/miR-192_nT1D	−0.25269	0.3447	0.7285	No
4	DOTAP/miR-193b_HC	DOTAP/miR-193b_nT1D	−0.24695	0.7332	0.9013	No
5	DOTAP/miR-185_HC	DOTAP/miR-185_nT1D	−0.33599	0.4274	0.7285	No
6	DOTAP/miR-195_HC	DOTAP/miR-195_nT1D	−0.4013	0.4274	0.7285	No
7	DOTAP/miR-455_HC	DOTAP/miR-455_nT1D	−0.30755	0.3847	0.7285	No
8	DOTAP/miR-375_HC	DOTAP/miR-375_nT1D	−0.61642	0.1859	0.7285	No
9	DOTAP/miR-129_HC	DOTAP/miR-129_nT1D	−0.65536	0.1212	0.7285	No
10	DOTAP_RNA41_HC	DOTAP_RNA41_nT1D	−0.41737	0.4490	0.7285	No
11	miR-122_HC	miR-122_nT1D	−0.60098	0.3591	0.7285	No
12	miR-192_HC	miR-192_nT1D	−0.47615	0.7837	0.9100	No
13	miR-193b_HC	miR-193b_nT1D	−0.54299	0.9264	1.0000	No
14	miR-185_HC	miR-185_nT1D	−0.59773	0.5228	0.7285	No
15	miR-195_HC	miR-195_nT1D	−0.6915	0.3153	0.7285	No
16	miR-455_HC	miR-455_nT1D	−0.08925	0.5199	0.7285	No
17	miR-375_HC	miR-375_nT1D	−0.49366	0.4642	0.7285	No
18	miR-129_HC	miR-129_nT1D	−0.57465	0.6466	0.8446	No

*Hedges’ g correction was used with 5000 re-samplings and 95% confidence interval to predict the effect of miRNA stimulation. Stimulation results with FDR *q*-Value < 0.1 were considered as significant. DOTAP, DOTAP synthetic vesicles; DOTAP/miRNA, miRNA implicated with DOTAP synthetic vesicles; K-, samples with only blood cells; miR, investigated miRNA alone; HC, healthy controls; nT1D, new onset type 1 diabetes participants.*

**TABLE 6B:** New-onset T1D vs. healthy controls EVs-miRNA *in vitro* CD107a+ degranulation (unpaired mean difference analysis results).

**CD8+CD107a+**	**Control**	**Test**	**Difference**	***P*-value mann_whitney**	***q*-Value**	**Discovery?**
0	K-_HC	K-_nT1D	0.973031	0.0409	0.4279	No
1	DOTAP_HC	DOTAP_nT1D	1.066027	0.0169	0.3537	No
2	DOTAP/miR-122_HC	DOTAP/miR-122_nT1D	0.230197	0.8500	1.0000	No
3	DOTAP/miR-192_HC	DOTAP/miR-192_nT1D	0.249794	0.7052	1.0000	No
4	DOTAP/miR-193b_HC	DOTAP/miR-193b_nT1D	0.952713	0.0692	0.4823	No
5	DOTAP/miR-185_HC	DOTAP/miR-185_nT1D	0.152561	0.4270	1.0000	No
6	DOTAP/miR-195_HC	DOTAP/miR-195_nT1D	0.259727	0.6229	1.0000	No
7	DOTAP/miR-455_HC	DOTAP/miR-455_nT1D	0.098524	0.9698	1.0000	No
8	DOTAP/miR-375_HC	DOTAP/miR-375_nT1D	0.267454	0.5966	1.0000	No
9	DOTAP/miR-129_HC	DOTAP/miR-129_nT1D	0.06552	0.6225	1.0000	No
10	DOTAP_RNA41_HC	DOTAP_RNA41_nT1D	0.486602	0.3442	1.0000	No
11	miR-122_HC	miR-122_nT1D	0.528305	0.6458	1.0000	No
12	miR-192_HC	miR-192_nT1D	0.490841	1.0000	1.0000	No
13	miR-193b_HC	miR-193b_nT1D	0.479991	0.7827	1.0000	No
14	miR-185_HC	miR-185_nT1D	0.431101	0.6466	1.0000	No
15	miR-195_HC	miR-195_nT1D	0.268122	0.9269	1.0000	No
16	miR-455_HC	miR-455_nT1D	0.716379	0.1109	0.5796	No
17	miR-375_HC	miR-375_nT1D	0.650574	0.2678	1.0000	No
18	miR-129_HC	miR-129_nT1D	0.476454	0.5209	1.0000	No

**CD56+CD107a+**	**Control**	**Test**	**Difference**	***P*-value mann_whitney**	***q*-Value**	**Discovery?**

0	K-_HC	K-_nT1D	0.569524	0.4956	0.9525	No
1	DOTAP_HC	DOTAP_nT1D	0.343142	0.5447	0.9525	No
2	DOTAP/miR-122_HC	DOTAP/miR-122_nT1D	0.19403	0.7623	0.9525	No
3	DOTAP/miR-192_HC	DOTAP/miR-192_nT1D	0.431914	0.5708	0.9525	No
4	DOTAP/miR-193b_HC	DOTAP/miR-193b_nT1D	0.623708	0.5964	0.9525	No
5	DOTAP/miR-185_HC	DOTAP/miR-185_nT1D	0.263588	0.7054	0.9525	No
6	DOTAP/miR-195_HC	DOTAP/miR-195_nT1D	0.537545	0.3073	0.9525	No
7	DOTAP/miR-455_HC	DOTAP/miR-455_nT1D	0.128074	0.9698	1.0000	No
8	DOTAP/miR-375_HC	DOTAP/miR-375_nT1D	0.272265	0.6230	0.9525	No
9	DOTAP/miR-129_HC	DOTAP/miR-129_nT1D	0.276433	0.7623	0.9525	No
10	DOTAP_RNA41_HC	DOTAP_RNA41_nT1D	0.275529	0.8203	0.9525	No
11	miR-122_HC	miR-122_nT1D	−0.30146	0.5200	0.9525	No
12	miR-192_HC	miR-192_nT1D	−0.508	0.2343	0.9525	No
13	miR-193b_HC	miR-193b_nT1D	−0.69441	0.5219	0.9525	No
14	miR-185_HC	miR-185_nT1D	−0.86097	0.2353	0.9525	No
15	miR-195_HC	miR-195_nT1D	−0.32652	0.5228	0.9525	No
16	miR-455_HC	miR-455_nT1D	0.273825	0.7907	0.9525	No
17	miR-375_HC	miR-375_nT1D	−0.6002	0.1709	0.9525	No
18	miR-129_HC	miR-129_nT1D	−1.23716	0.0828	0.9525	No

*Hedges’ g correction with 5000 re-samplings and 95% confidence interval was used to predict the effect of miRNA stimulation. Stimulation results with FDR *q*-Value < 0.1 were considered as significant. DOTAP, DOTAP synthetic vesicles; DOTAP/miRNA, miRNA implicated with DOTAP synthetic vesicles; K-, samples with only blood cells, miR, investigated miRNA alone; HC, healthy controls; nT1D, new onset type 1 diabetes participants.*

The stimulation of immune cells with EVs’ miRNAs influenced the regulation of the immune system. This potentially contributes to the pathogenesis of T1D with modulated T-cell activation and cytotoxicity. Interestingly, vesicles containing miRNAs modulated T-cell activation more intensively in HC compared to nT1D individuals. These results pawed the way for the next step: the investigation of pathway associated with immune cells’ EVs internalization, miRNA interaction, and *in vitro* inhibition of this pathway.

### Extracellular Vesicles’ Intracellular Accumulation in Phagocytes

**FIGURE 5:**
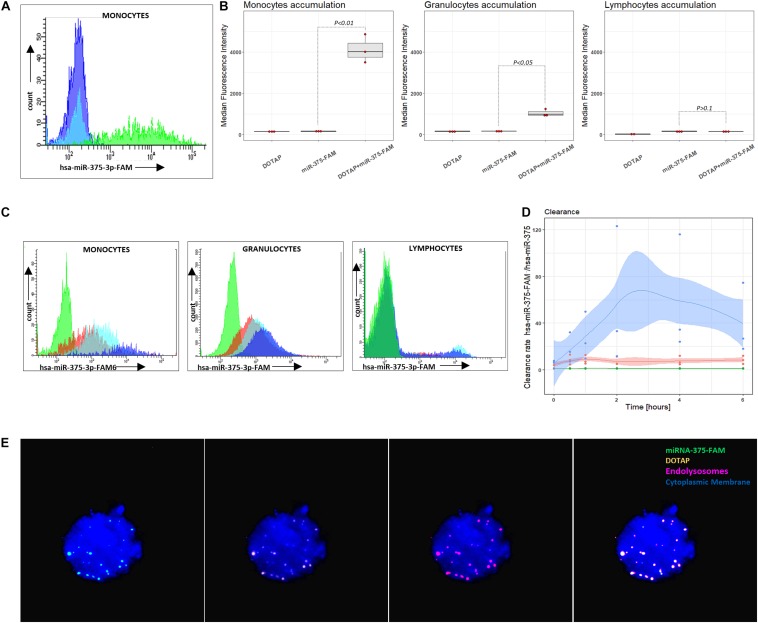
Vesicle internalization and intracellular aggregation. **(A)** Representative results of flow cytometry DOTAP, hsa-miRNA-375-FAM (3’FAM labeled miRNA) and DOTAP/hsa-miRNA-375-FAM transfection in the whole blood samples, detecting miRNA-FAM in monocytes, granulocytes, lymphocytes after [the observed effects were assessed 4h after the stimulation; dark blue: DOTAP, light blue: hsa-miRNA-375-FAM, green peak: DOTAP/hsa-miRNA-375-FAM]. **(B)** Monocytes, granulocytes and lymphocytes median fluorescence intensity regarding the transfected DOTAP, fluorescent-labeled hsa-miRNA-375-FAM and DOTAP/hsa-miRNA-375-FAM [*P*-values represent a paired Hedges’ g mean difference between hsa-miRNA-375-FAM and DOTAP/hsa-miRNA-375-FAM: *P < 0.05* significant difference, *N* = 3]. **(C)** Representative results of flow cytometry increasing monocytes, granulocytes, lymphocytes fluorescence with the time of blood cells exposed to DOTAP formed vesicles with FAM fluorescent-labeled miRNA [green peak: non-labeled hsa-miR-375-3p, 5 min; red color:hsa-miR-375-3p-FAM, 5min; light blue: hsa-miR-375-3p-FAM, 2 h; dark blue: hsa-miR-375-3p-FAM, 6 h]. **(D)** Monocytes, granulocytes and lymphocytes relative mean fluorescence ratio of DOTAP/hsa-miR-375-3p-FAM with DOTAP/hsa-miR-375-3p within 6 h from the beginning of the transfection (DOTAP/hsa-miR-375-FAM / DOTAP/hsa-miR-375) (solid lines: average value, shaded areas: standard deviation; *N* = 3). **(E)** Fluorescent microscopy image of DOTAP/miRNA-375-3p-FAM up-taken vesicles (green) with DOTAP vesicles (orange), located in endolysosomes and lysosomes (red); cytoplasmic membrane (blue).

miRNA intracellular accumulation in cells of the immune system occurred only if miRNA was transfected with vesicles; bare miRNA did not enter the cells of the immune system (Figures 5A,B). The analysis of the DOTAP/miRNA internalization process with fluorescent-labeled miRNA by fluorescence microscopy (Figure 5E) and flow cytometry revealed preferential vesicles internalization by monocytes and, to a lesser degree, by granulocytes (Figures 5B–D), while lymphocytes internalization of DOTAP/miRNA was negligible. These results indicate the essential role of the endolysosomal antigen-presentation pathway of monocytes and granulocytes, and the internalized miRNAs associated immune system activation.

### TLR7/8 miRNA Activation

**FIGURE 6:**
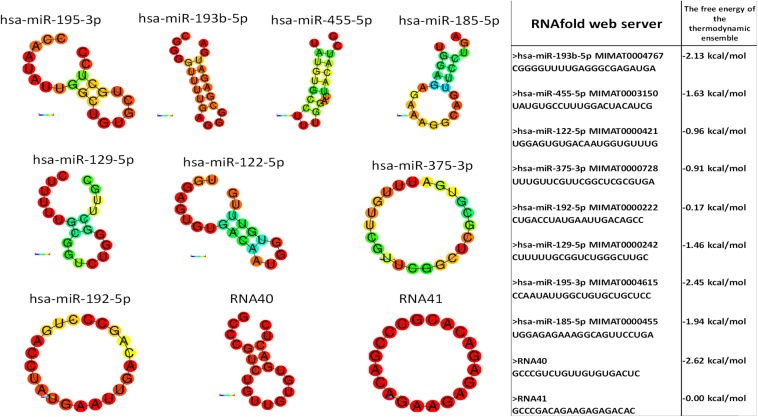
miRNA structure prediction with thermodynamics parameters. RNAfold structure prediction of studied miRNA. Red-colored nucleotides in the predicted structure represent a high probability of nucleotide in predicted structure, blue-colored nucleotides low probability. The table shows the thermodynamic data results of the RNAfold server tool for predicted miRNA structures.

Monocytes and granulocytes express endosomal TLR7 and TLR8 (TLR7/8), capable of detecting intracellular single-stranded pathogen RNA (ssRNA) in the endolysosomal pathway, where the TLR7/8 activation is RNA structure and sequence (uridine nucleotide accessibility) dependent (Gantier et al., 2008). The comparison of miRNA structures and immune response shows the highest immune activation with hsa-miR-455-5p, likely because of exposed uridines in the loop and prime ends of the sequence. Moderate stimulation response of other studied miRNAs containing accessible uridines was observed. The non-response of hsa-miR-193b-3p was probably the consequence of the inaccessibility of paired uridines in dsRNA stem-loop miRNAs structure (Figure 6).

**FIGURE 7:**
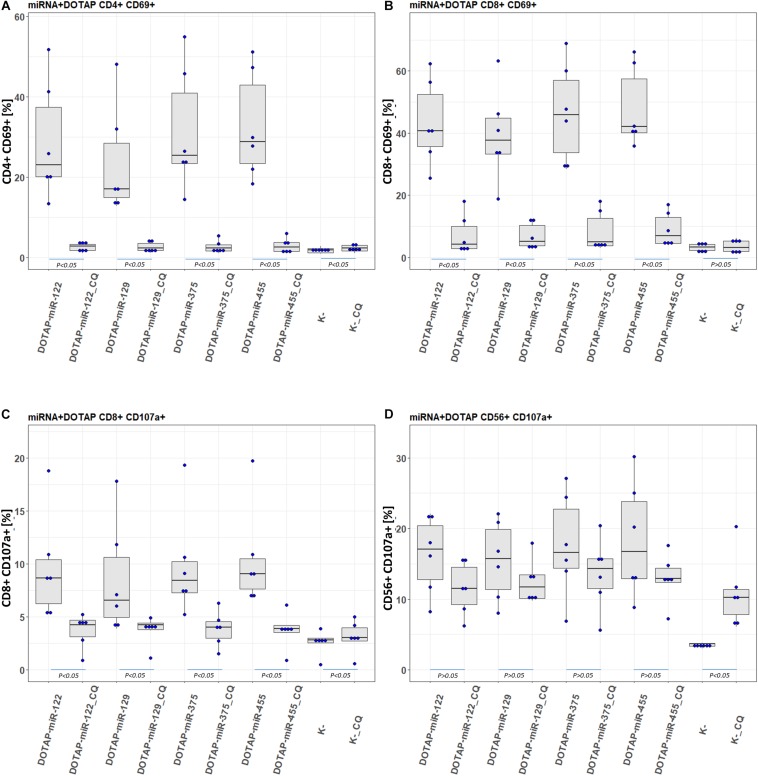
Chloroquine miRNA/DOTAP inhibition. Chloroquine (CQ) inhibition of hsa-miR-122-5p, hsa-miR-455-5p, hsa-miR-375-3p, hsa-miR-129-5p DOTAP vesicle stimulation effect on expression of early transition activation marker (CD69) on panels **(A)** CD4+ and **(B)** CD8+ T-cells and degranulation marker (CD107a) on panels **(C)** CD8+ T-cells and **(D)** CD56+ NK cells after 21h stimulation [box plot: Q1, median, Q3; whisker lines: 1.5 IQR; *N* = 6; K- negative control, *P-*values under blue lines represent a paired Hedges’ g mean difference analysis significance: *P* < 0.05 significant difference].

To confirm the involvement of the endolysosomal TLR7/8 in the activation of the immune system and to investigate potential molecular therapeutic targets, the activated pathway was inhibited by an endosomal TLR7/8 inhibitor. Chloroquine (CQ) decreases ssRNA binding affinity to the TLR7/8 (Kuznik et al., 2011), thus preventing downstream activation of signaling pathways. The CQ co-application with vesicle-miRNAs resulted in an efficient inhibition of the T-cell activation and less effective inhibition of NK cells (Figure 7). This miRNA activation of TLR7/8 did not depend on the synthetic vesicle delivery system: The DOTAP/miRNA vesicle immunomodulation was comparable to Lipofectamine RNAiMAX (Supplementary Tables S1A,B).

### Cytokine/Chemokine Profiling

**FIGURE 8:**
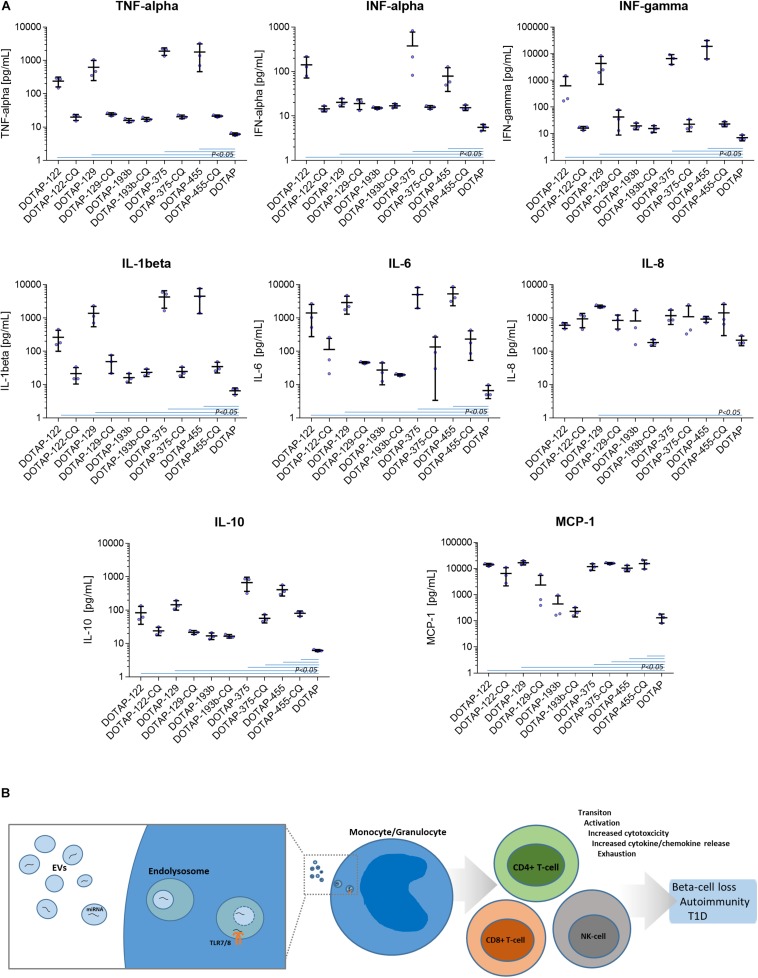
**(A)** Cytokine/chemokine release response as a result of DOTAP/miRNA transfection. Blue lines in graphs show Kruskal-Wallis ANOVA test *P*-values < 0.05 significant difference of DOTAP/miRNA compared to DOTAP negative control blue dots: cytokine/chemokine assessed levels; mean, error bars SD [*N* = 3]. **(B)** The proposed model of EVs derived miRNAs immune system modulation in type 1 diabetes via TLR7/8. EVs miRNAs immune system activation via TLR7/8 increases proliferation and cytotoxicity of the immune system of effector cells, cytokine/chemokine release and immune system exhaustion, dysregulation, and autoimmunity.

The DOTAP/miRNA activation was also associated with the increased cytokine and chemokine release. Vesicle miRNAs immune cell activation was evaluated with the inflammation cytokine profile assessment. The investigated miRNAs hsa-miR-122-5p, hsa-miR-129-5p, hsa-miR-375-3p, and hsa-miR-455-5p delivered with vesicles, but not hsa-miR-193b-5p, resulted in the increase of IFN-alpha, IFN-gamma, TNF-alpha, IL-1beta, IL-10, IL-6, and MCP-1 release (all *p* < 0.05) (Figure 8A and Supplementary Table S2).

The DOTAP/miRNA transfection did not result in a significant increase of IL-8, IL-17A, IL-18, IL-23, and IL-33 levels, moreover, IL-12p70 and IL-17 levels were below the detection limit. miRNA vesicle transfections with the CQ inhibitor did not differentiate with the cytokine/chemokine release levels compared to negative control (DOTAP), with some exception of MCP-1 and IL-10 (Figure 8A).

These data indicate the importance of human self-derived EVs miRNAs as auto-agents, which can modulate the immune system through the TLR7/8 pathway. However, we did not test the miRNA immune system activation via miRNA-mRNA interactions.

## Discussion

Micro-RNAs are primarily involved in the intracellular regulation of mRNA translation, mRNA degradation, and gene expression (Djuranovic et al., 2012; Cottrell et al., 2017). Furthermore, miRNAs can be packed in EVs released from maternal cells, and can modulate specific cellular processes in the recipient cells (Kosaka et al., 2013). A few studies investigated EVs protein cargo (Cianciaruso et al., 2017; Hasilo et al., 2017; Rutman et al., 2018) and total plasma miRNA in T1D (Garcia-Contreras et al., 2017) so far, however, the immunomodulatory role of EVs miRNA has not been well established. To our knowledge, this is the first EVs study demonstrating beta-cell-released EVs presence in blood plasma using TEM (Figure 1), and the potential role of EVs miRNA in the etiology of T1D. The TEM characterization demonstrated the presence of Langerhans islets’ EVs in plasma in a non-quantitative manner. We further specified EVs fraction using NGS miRNA profiling, which included EVs miRNA amplification and allowed low count miRNA detection in the samples with limited sample volume.

Using NGS sequencing and comparison of blood plasma EVs profiles of healthy controls, nT1D individuals with an active beta-cell destruction, and 10yT1D participants with non-detectable beta-cell activity or autoimmunity process, we characterized differentially expressed EVs miRNA in T1D (Figures 2A–C). The most prominently overexpressed miRNAs in the nT1D cohort was hsa-miR-122-5p, previously associated with liver pathology (Hu et al., 2012), metabolism regulation (Willeit et al., 2017; Barajas et al., 2018), and T1D (Åkerman et al., 2018). Elevated hsa-miR-192-5p and hsa-miR-193b-5p in T1D are reported as markers of prediabetes in the adult population (Párrizas et al., 2015). Additionally, some EVs miRNAs differentially expressed in 10yT1D individuals could indicate the early development of diabetic complications (La Sala et al., 2016). Due to the variability of preserved beta-cell mass at the onset of T1D and differences in the autoimmune process activity, the differentially expressed miRNA profiles in T1D individuals might not be clearly associated with beta-cell damage.

The EVs enriched fraction included EVs from all parts of a human body, including Langerhans islets EVs, EVs of the cells of the immune system and other EVs released as the result of a metabolic imbalance. We cannot declare the miRNAs as beta-cell or other tissue specific. However, some miRNA were differentially expressed in T1D individuals, probably as a result of autoimmunity and metabolic imbalance.

Individuals with Langerhans islets transplantation were additionally included in our study to investigate the miRNA during the active (intensive) beta-cell damage and destruction: beta-cells are exposed to the transplantation stress and partial destruction, which can reflect in stress released EVs. The differential expression analysis of TX patients’ EVs miRNA profiles demonstrated increased expression of hsa-miR-375-3p, hsa-miR-129-5p, hsa-miR-200b-5p, and hsa-miR-216b-5p (Figure 2D). The most prominent increase was observed for hsa-miR-375-3p, the miRNA with the highest expression in beta-cells (LaPierre and Stoffel, 2017), and associated with beta-cell destruction at the T1D onset, although the published data are not unequivocal (Marchand et al., 2016; Erener et al., 2017). Other miRNAs were also likely released from beta-cells, where hsa-miR-200 regulate beta-cell apoptosis and miR-126 glucose promoted/inhibited proliferation (Belgardt et al., 2015; Tao et al., 2016).

Eight significantly differentially expressed miRNA were selected for further *in vitro* functional testing on whole blood cells from participants with nT1D and HC. hsa-miR-122-5p, hsa-miR-192-5p, and hsa-miR-193b-5p had higher expression in nT1D, while hsa-miR-185-5p was overexpressed and hsa-miR-195-3p and hsa-miR-455-5p were under-expressed in 10yT1D participants compared to nT1D and HC. Additionally, we also included hsa-miR-375-3p and hsa-miR-129-5p, which were the most significantly differentially expressed EVs miRNAs in the TX patients. These *in vitro* results were obtained on the complex whole blood samples with preserved immune cells sub-populations and immune system interactions in T1D-affected participants that represent a more reliable model compared to the one-dimensional experiments on homogenous cell cultures or model organisms with differences in the immune signaling (Brehm et al., 2012). The results of this functional miRNA study revealed significantly increased NK and T-cell early transition proliferation and cytotoxicity when cells were exposed to diabetes-associated EVs miRNAs in vesicular form, but not bare miRNAs alone (Figure 3). Furthermore, nT1D participants presented considerably lower CD69+ CD4+ activation response compared to HC, indicating the potential effect of the immune exhaustion and tolerance (Yi et al., 2010; Frenz et al., 2016) as the result of previous exposure to miRNAs involved in the development of T1D autoimmunity and beta-cell destruction. The vesicle delivered miRNA also resulted in the increased release of IFN-alpha, IFN-gamma, TNF-alpha, IL-1beta, IL-6 IL-10, and MCP-1 (Figure 8A), which indicates TLR7/8 associated cytokine/chemokine release (Gantier et al., 2008; Salvi et al., 2018; Pluta et al., 2019). The same cytokines/chemokines are reported as increased in the individuals with T1D autoimmunity (Fatima et al., 2016; Waugh et al., 2017).

This difference in the *in vitro* stimulation indicated the potential role of EVs’ delivered miRNAs in the regulation of the immune system in T1D-related to beta-cell damage (Figure 4A). *In vitro* miRNA tests were performed with approximately ten-times higher miRNA concertations compared to the plasma EVs-RNA concentrations estimated from the total isolated amount of blood plasma EVs RNA (Supplementary Figure S7). However, EVs with miRNAs released in affected tissues with prolonged exposure to stress factors might be sufficient to activate the immune system and to drive autoimmunity. The vesicles accumulated in phagocytes (monocytes and granulocytes) (Figures 5B–D) where TLR7/8 are expressed and participate in the ssRNA immune activation in the anti-viral innate immunity (Aharon et al., 2008; Cros et al., 2010; Lester and Li, 2014). The TLR7/8 innate immunity activation is not T1D specific, but is probably involved in the immune system modulation and autoimmunity (Salvi et al., 2018; Pluta et al., 2019). This is in line with the ssRNA-structure dependent TLR7/8 activation (Gantier et al., 2008), with the accessible uridines in the loop and prime ends of the sequence (Figure 6). Additionally, the vesicle miRNAs activation of the TLR7/8 pathway was specified with the utilization of CQ, which inhibited vesicle miRNA immunomodulation and showed strong evidence of TLR7/8 pathway activation (Figure 7). However, additional possible direct effects of EVs miRNA content on T-cells (Zhang et al., 2006; Wang et al., 2008) or direct miRNA-mRNA regulation effects were not excluded.

In light of the reported results, we proposed a novel role of EVs’ miRNAs in the etiology of T1D, suggesting the immune response modulation via the TLR7/8 activation and EVs miRNAs involvement into inflammation and autoimmunity. Beta-cells-released EVs carrying miRNAs could enter the surrounding tissues where EVs are internalized and accumulated in phagocytes, where they trigger endosomal TLR7/8 mediated response. This in turn led to the release of cytokines in the antigen-independent manner and the activation of other sub-populations of the innate and adaptive immune system, resulting in the increased cell activation, proliferation, cytotoxicity, inflammation and cytokine release (Mohammad Hosseini et al., 2015; Farrugia and Baron, 2017; Salvi et al., 2018). EVs released by other cells can enhance the immune response and contribute to the development of autoimmunity during the inflammation stress and metabolic imbalance (Robbins et al., 2016). The prolonged exposure to EVs miRNA could result in the immune system exhaustion and lower immune activation response to vesicle derived miRNA in nT1D. Immune exhaustion with higher CD8+ T-cell activation compared to CD4+ T-cells could represent the specific autoimmune phenotype (Morawski and Bolland, 2018) which promotes the autoimmune beta-cell destruction and overt T1D (Figure 8B).

One of the most common mechanisms proposed in autoimmunity is an innate immune cell hyper-activation, such as dendritic cells (DC), that can overstimulate T lymphocytes (Di Marco et al., 2018). TLR7/8 activation in DC triggers the release of pro-inflammatory cytokines (Morse and Horwitz, 2017; Salvi et al., 2018) and immune cells intercellular interactions, resulting in expanded T-cell-clones and auto-immune risk phenotype, in both mice and humans (Ara et al., 2018; Morawski and Bolland, 2018). The TLR7/8 activation of the downstream immune system and auto-immune risk phenotype contributes to beta-cell destruction, promotes diabetes development, and causes T-cell exhaustion (Lee et al., 2011). Our results are in line with the published miRNA TLR7/8 immunomodulation (Salvi et al., 2018; Pluta et al., 2019), however, we could not link them directly to any specific stage of the T1D development.

TLR7/8 receptors are part of anti-viral innate immunity and can be involved in numerous viral infections as triggering factors associated with T1D (Katsarou et al., 2017; Paschou et al., 2017). Viral infections can also impair and promote cell stress EVs release (Deschamps and Kalamvoki, 2018), impaired EVs cargo loading (de Jong et al., 2012), and contribute to the dysregulation of the immune system, breakdown of self-tolerance, and potential development of autoimmunity (Cianciaruso et al., 2017). Here we showed that differentially expressed human miRNAs in the blood of people with T1D packed into vesicles mimic the action of pathogen ssRNA recognized by TLR7/8. The activation was potentially triggered through the principle of molecular/viral mimicry with pathogenic viral ssRNAs. Additionally, as the immune system is activated in an antigen-independent manner, it also supports the EVs miRNA-induced bystander immune activation and autoimmunity (Pacheco et al., 2019).

The effect of the circulating EVs’ miRNAs on blood immune cells in our study indicated the systemic immune response. However, it is reported that ssRNA molecules can promote the monocyte activation and differentiation into tissue macrophages (Krutzik et al., 2005; Saha et al., 2017), which can infiltrate into the affected target tissues (Eng et al., 2018), resulting in an enhanced local immune response. Moreover, Langerhans islets contain resident macrophages that hold an essential role in the local islet immunity, development of acute insulitis, and autoimmunity (Carrero et al., 2016, 2017). EVs ssRNA TLR7/8 activation also leads to impaired monocyte differentiation into DC (Assier et al., 2007), DC modulation, T-reactive clone selection, and Treg/Teff equilibrium modulation (Wang et al., 2008; Anz et al., 2010; Dominguez-Villar et al., 2015). Our *in vitro* miRNAs stimulation results indicated a substantial EVs miRNAs’ immunoregulatory effect on the systemic innate and adaptive immunity as well as their possible involvement on a local tissue level in the development and progression of autoimmunity and beta-cell loss in T1D.

Collectively, our study provided data describing the importance of a complex involvement of EVs-derived human miRNAs in the regulation of the immune system, and potentially in the development of T1D autoimmunity, with the implications for developing strategies for the prevention and treatment of T1D-related immune processes.

## Data Availability Statement

The datasets GENERATED for this study can be found in NCBI http://www.ncbi.nlm.nih.gov/bioproject/591780.

## Ethics Statement

The study protocol was approved by the Republic of Slovenia National Medical Ethics Committee (No. 29/02/2013 and 31/04/2016), and principles of the Declaration of Helsinki were followed. Written informed consent was obtained from the participants or participants’ parents prior to the study.

## Author Contributions

TT developed the hypothesis, designed the experimental approach, performed the experimental work, analyzed the data, coordinated the project, and wrote the manuscript. JK designed the experimental approach, developed the hypothesis, statistically analyzed the data, and wrote the manuscript. KP designed the experiments and performed the flow cytometry studies and analysis. SH performed the TEM sample preparation and imaging. KD and NB provided the diabetes and healthy control samples. KTP and MD read the manuscript and provided the feedback. PV contributed to TEM experimental approach, data interpretation, and characterization. LP and EB designed the experimental approach, provided and prepared the Langerhans islet medium and Langerhans islets transplantation patients’ plasma samples. AI discussed the hypothesis, flow cytometry study design, interpreted flow cytometry data, and wrote the manuscript. TB conceived the hypothesis, led the project, coordinated the project, and wrote the manuscript. All authors reviewed and approved the final manuscript.

## Conflict of Interest

The authors declare that the research was conducted in the absence of any commercial or financial relationships that could be construed as a potential conflict of interest.

## Footnotes

1 https://atlasantibodies.com/
