# Supplementary material for: Extracellular Vesicles Derived Human-miRNAs Modulate the Immune System in Type 1 Diabetes
Source: Front Cell Dev Biol. 2020 Mar 31;8:202. doi: 10.3389/fcell.2020.00202 (PMC7136501; doi:10.3389/fcell.2020.00202)
Supplement: Supplementary file 1 [file Data_Sheet_1.docx]

**Supplementary Material**

**Extracellular vesicles derived human miRNAs modulate the immune system in type 1 diabetes**

**
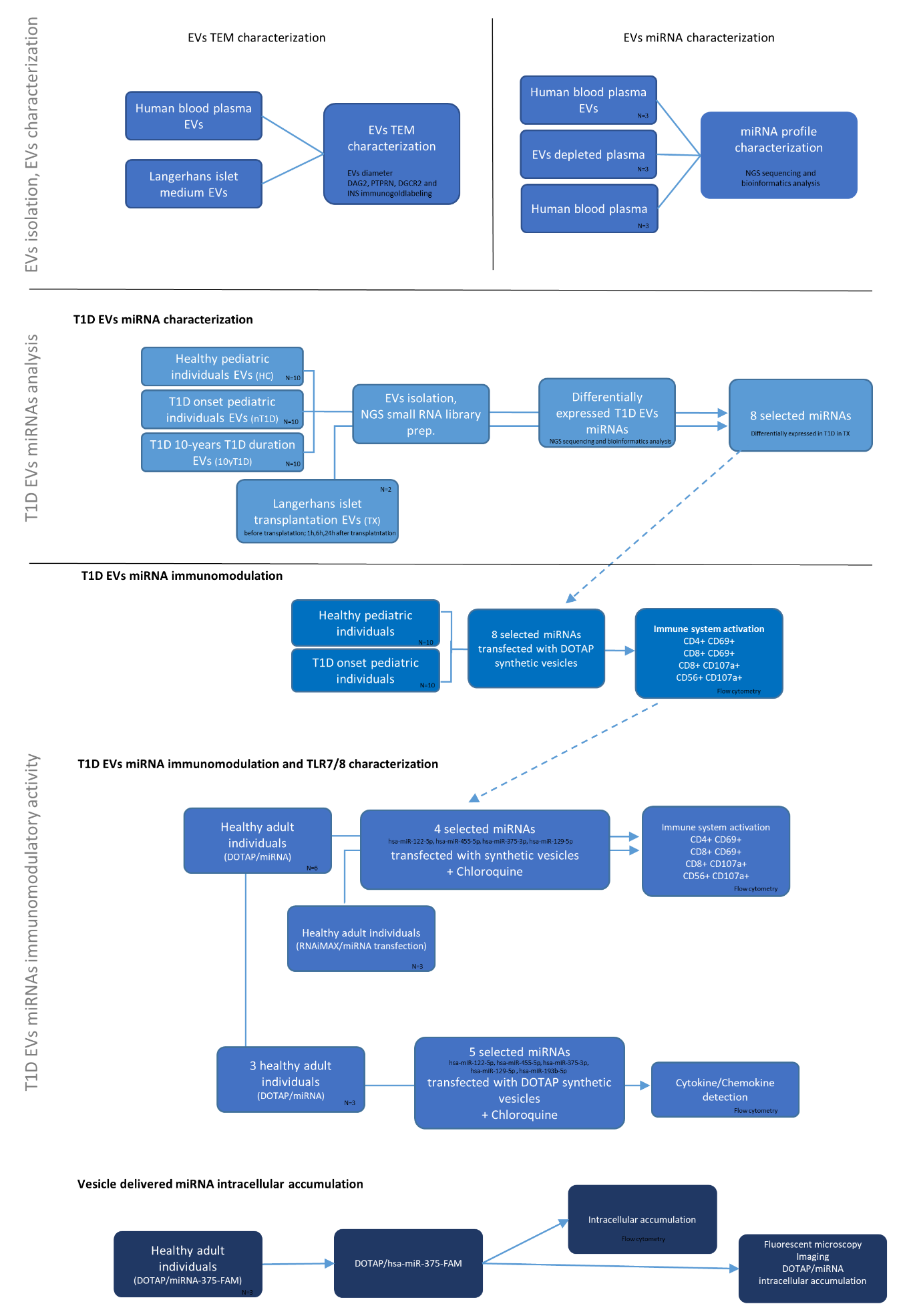
**

**Supplementary Figure S1:** Schematic diagram of study workflow with experiments. The study was designed in three steps: 1) plasma and Langerhans islet medium EVs isolation and TEM EVs imaging; 2) comparative T1D and TX EVs plasma miRNA Next-generation sequencing and analysis; 3) in vitro differentially expressed vesicle miRNA effect study on the human whole blood immune cells.

**
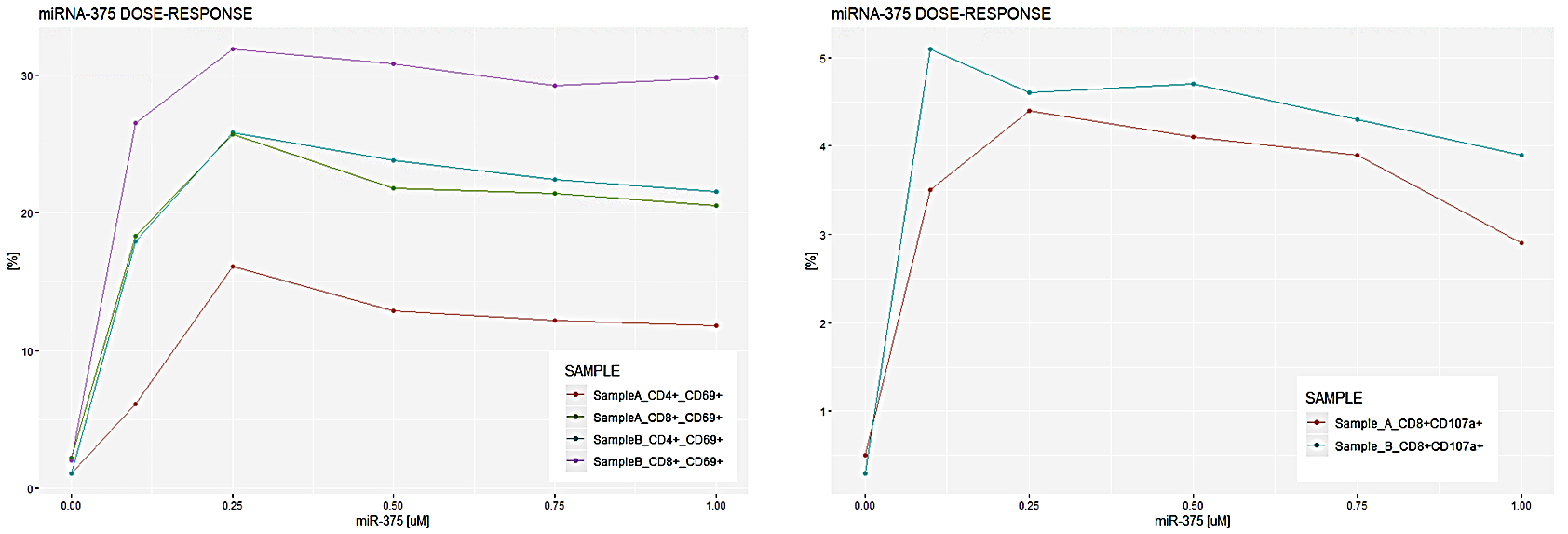
**

**Supplementary Figure S2:** miRNA-375 early activation and cytotoxicity T-cell miRNA dose-response. DOTAP/hsa-miRNA-375-3p miRNA (titration) dose response of two adult whole blood samples with the measured effect of CD69 early transition activation marker on CD4+ and CD8+ and CD8+ CD107a+ cytotoxicity marker expression, 21h after the stimulation.


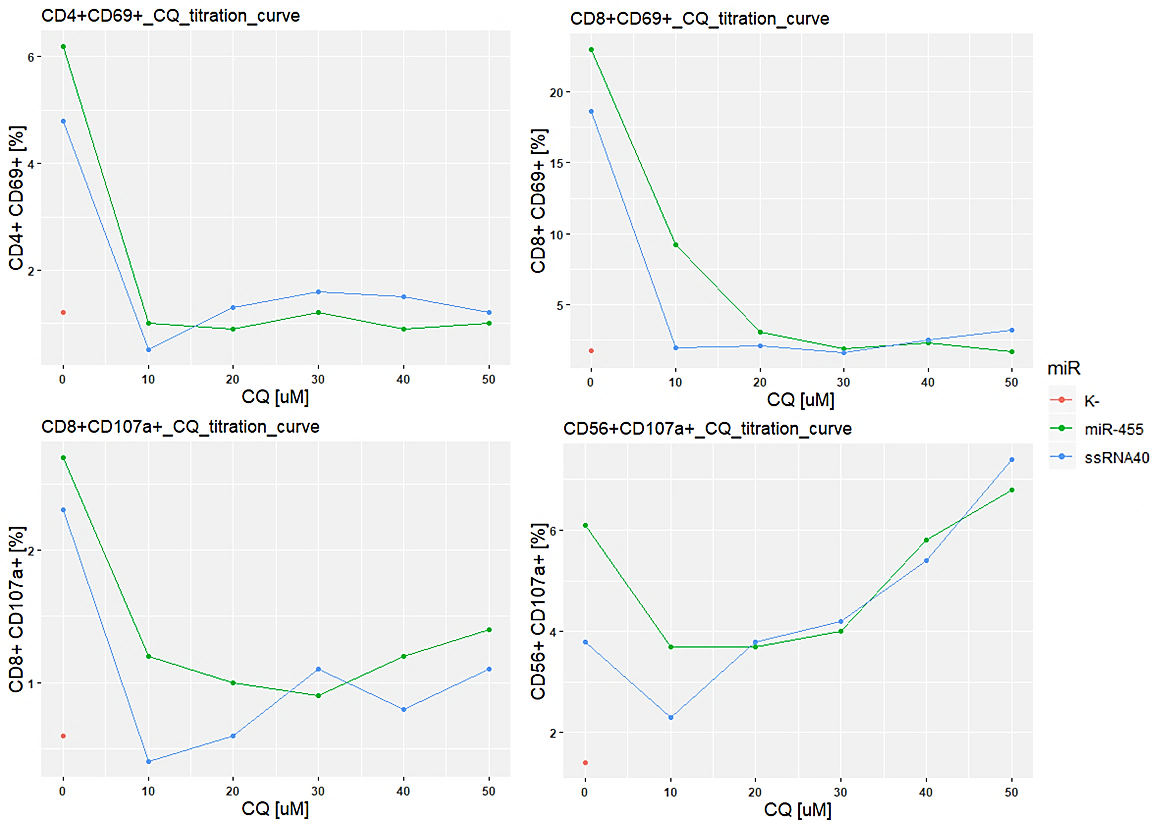


**Supplementary Figure S3:** CQ titration curve.

DOTAP/hsa-miRNA-455-5p and ssRNA40/LyoVec (TLR7/8 activation positive control) stimulation effect inhibition with different chloroquine (CQ) concentrations. CQ inhibition effect on CD4+ CD69+ and CD8+ CD69+ early activation transition marker and CD8+ CD107a+ and CD56+ CD107a+ cytotoxicity marker expression. CQ concentration higher than 50µM (75 and 100 µM) showed the cytotoxic effect on the whole blood cells and are not reported on graphs (K- negative control).

**
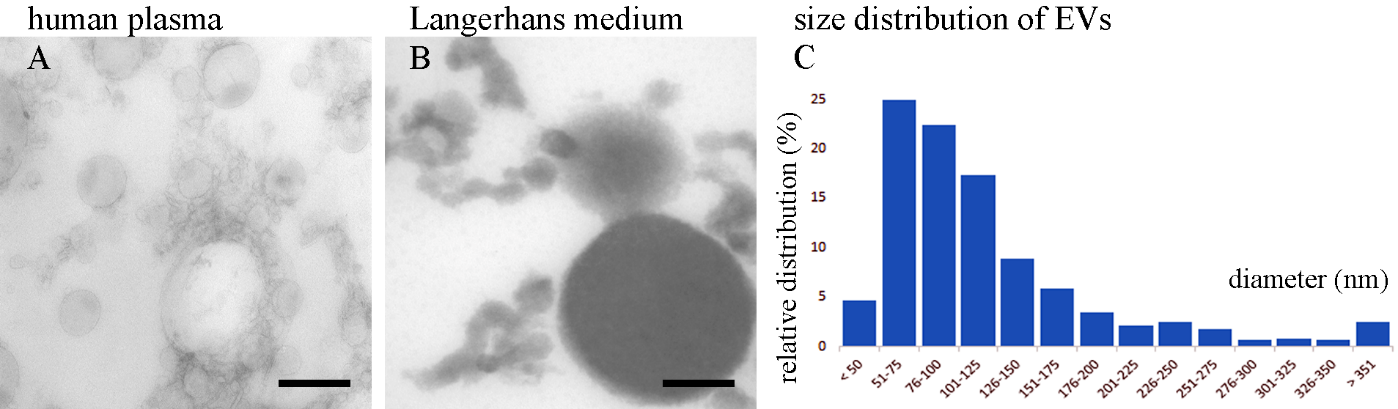
**

**Supplementary Figure S4:** Ultrastructure (A, B) and size distribution (C) of extracellular vesicles isolated from human blood plasma (A) and from the medium of ex vivo cultured human Langerhans islets (B, C). Bars: 200 nm. TEM analysis statistics: Ultrathin sections of in vitro cultured human Langerhans islets were picked on a honey-comb TEM grids and observed with CM100 transmission electron microscope (Philips) running at 80 kV. The sample was analysed in the straight line of 12 🡪 6 o’clock direction, with taking a micrograph in each second grid hole with the sample present. Eleven micrographs collected at 8900 X magnification was imported into ImageJ software (Version 1.52s) and the diameter of 723 vesicles was determined using a measure tool. Results were imported to MS Excel 2016 (Microsoft) and a relative distribution of vesicles per diameter class was presented in a graph.

**
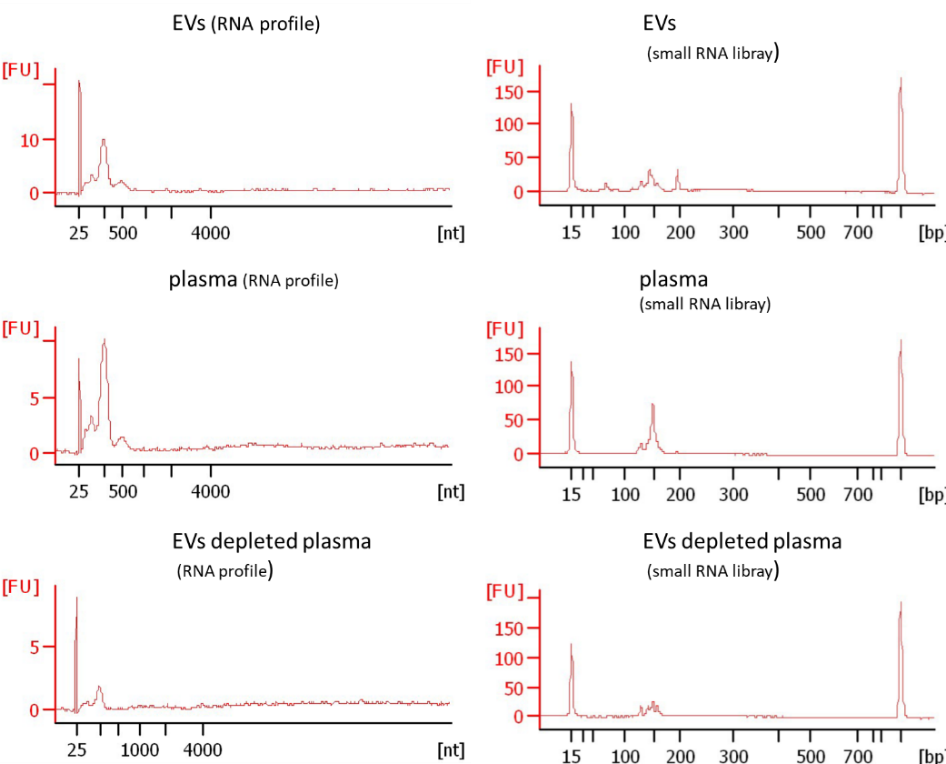
**

**Supplementary Figure S5: Isolated EVs plasma fraction, plasma and EVs depleted plasma RNA profiles and small RNA libraries electropherograms.** RNA profiles were characterized using RNA 6000 Pico kit and small RNA libraries using DNA 1000 kit on Agilent Bioanalyzer 2100.

**Supplementary Figure S6:** miRNA profile sample characterization and sample clustering**.** Heat map presents expression analysis profile sample clustering. S1, S2 and S3 represent healthy adult individuals whose plasma, isolated EVs fraction and EVs depleted plasma samples were sequenced and analysed.


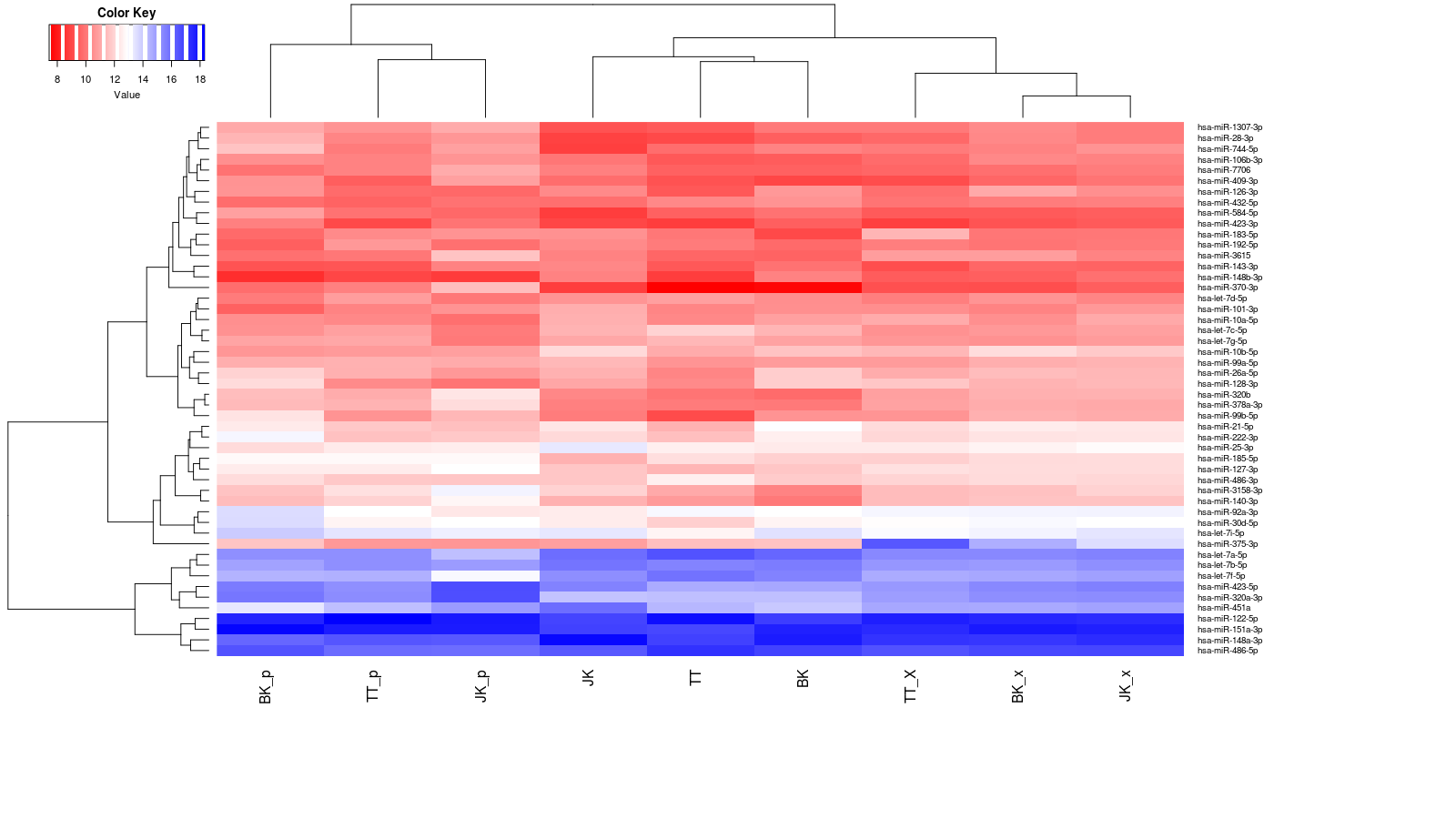


S1 S2 S3

S1 S2 S3

S1 S2 S3

PLASMA

EVs

EVs DEPLETED PLASMA

**
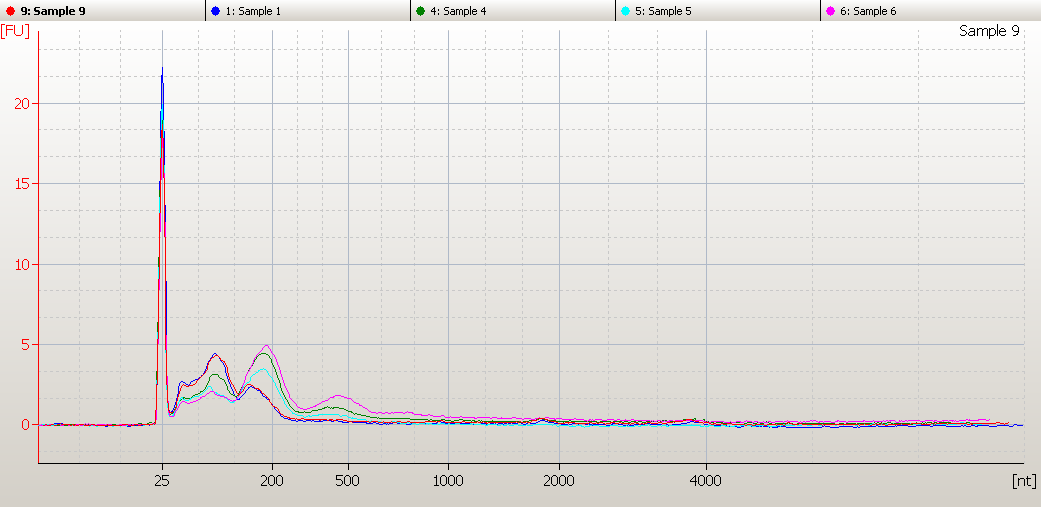
**

**Supplementary Figure S7:** Blood plasma EVs-RNA mass/concentration.

RNA (Bioanalyzer Pico 6000) electropherogram represents amount of RNA in isolated EVs fraction regarding to starting plasma sample volume. The estimated concentration of EVs-RNA isolated from 1mL plasma in Sample 1: 73nM (2.35 ng); Sample 2: 72nM (2.33 ng); Sample 4: 94nM (3.01 ng); Sample 5: 65nM (2.09 ng); Sample 6: 96nM (3.09 ng); Sample 9: 75nM (2.41ng). (estimated average RNA length: 100 base) (moles ssRNA (mol) = mass of ssRNA (g)/((length of ssRNA (nt) x 321.47 g/mol) + 18.02 g/mol).

**Supplementary Table 1A: DOTAP/ miRNA and RNAiMAX Lipofectamine/miRNA effect on CD69+ T‑cell activation (paired mean difference analysis).** Hedges' g correction with 5000 resamplings and 95% confidence interval was used to predict the effect of miRNA stimulation. Stimulation results with FDR q-value < 0.05 were considered as significant. DOTAP: DOTAP synthetic vesicles, DOTAP_miRNA: miRNA transfected with DOTAP synthetic vesicles; Lipo: RNAiMAX Lipofectamine synthetic vesicles, Lipo_miRNA: miRNA transfected with RNAiMAX Lipofectamine, K-: samples with only blood cells, miR: investigated miRNA alone; N=3.

| **CD4+ CD69+** | **control** | **test** | **difference** | **Pvalue wilcoxon** | **statistic_paired students_t** | **pvalue_paired_students_t** | **q value** | **Discovery?** |
| --- | --- | --- | --- | --- | --- | --- | --- | --- |
| 0 | K- | K-_CQ | 0.824052 | 0.108809 | -1.35501 | 0.308167 | 0.201885 | No |
| 1 | Lipo | Lipo_CQ | 0.55375 | 0.108809 | -4.25 | 0.051153 | 0.053711 | No |
| 2 | Lipo_miR-122 | Lipo_miR-122_CQ | -8.68332 | 0.108809 | 48.37967 | 0.000427 | 0.003587 | Yes |
| 3 | Lipo_miR-129 | Lipo_miR-129_CQ | -8.89535 | 0.108809 | 18.87252 | 0.002796 | 0.007429 | Yes |
| 4 | Lipo_miR-375 | Lipo_miR-375_CQ | -11.1144 | 0.108809 | 15.80331 | 0.00398 | 0.007429 | Yes |
| 5 | Lipo_miR-455 | Lipo_miR-455_CQ | -9.66166 | 0.108809 | 22.67837 | 0.001939 | 0.007429 | Yes |
| 6 | K- | Lipo_miR-122 | 11.95204 | 0.108809 | -19.1501 | 0.002716 | 0.007429 | Yes |
| 7 | K- | Lipo_miR-129 | 15.91795 | 0.108809 | -25.9243 | 0.001485 | 0.007429 | Yes |
| 8 | K- | Lipo_miR-375 | 35.38419 | 0.108809 | -56.462 | 0.000314 | 0.003587 | Yes |
| 9 | K- | Lipo_miR-455 | 10.63028 | 0.108809 | -16.6638 | 0.003582 | 0.007429 | Yes |
| 10 | K- | Lipo_miR-122_CQ | 1.535088 | 0.108809 | -2.53432 | 0.126759 | 0.112082 | No |
| 11 | K- | Lipo_miR-129_CQ | 0.982012 | 0.108809 | -1.58944 | 0.252913 | 0.184736 | No |
| 12 | K- | Lipo_miR-375_CQ | 1.427769 | 0.108809 | -2.24792 | 0.153573 | 0.129001 | No |
| 13 | K- | Lipo_miR-455_CQ | 1.296562 | 0.10247 | -2.16667 | 0.162596 | 0.130077 | No |
| 14 | DOTAP | DOTAP_CQ | 0.358261 | 0.108809 | -3.27327 | 0.082015 | 0.076547 | No |
| 15 | DOTAP_miR-122 | DOTAP_miR-122_CQ | -3.08072 | 0.108809 | 5.920323 | 0.027365 | 0.035364 | Yes |
| 16 | DOTAP_miR-129 | DOTAP_miR-129_CQ | -4.74295 | 0.108809 | 17.61362 | 0.003208 | 0.007429 | Yes |
| 17 | DOTAP_miR-375 | DOTAP_miR-375_CQ | -3.174 | 0.108809 | 3.783848 | 0.063286 | 0.062541 | No |
| 18 | DOTAP_miR-455 | DOTAP_miR-455_CQ | -5.48167 | 0.108809 | 10.45038 | 0.009033 | 0.013591 | Yes |
| 19 | K- | DOTAP_miR-122 | 3.372766 | 0.108809 | -5.26187 | 0.034272 | 0.038385 | Yes |
| 20 | K- | DOTAP_miR-129 | 6.299035 | 0.108809 | -10.0753 | 0.009708 | 0.013591 | Yes |
| 21 | K- | DOTAP_miR-375 | 3.665091 | 0.108809 | -5.55878 | 0.030872 | 0.037046 | Yes |
| 22 | K- | DOTAP_miR-455 | 7.020637 | 0.108809 | -10.9681 | 0.00821 | 0.013591 | Yes |
| 23 | K- | DOTAP_miR-122_CQ | 0.747403 | 0.285049 | -1.2069 | 0.350847 | 0.218305 | No |
| 24 | K- | DOTAP_miR-129_CQ | 0.846138 | 0.108809 | -1.365 | 0.305523 | 0.201885 | No |
| 25 | K- | DOTAP_miR-375_CQ | 0.720076 | 0.179712 | -1.14571 | 0.370512 | 0.222307 | No |
| 26 | K- | DOTAP_miR-455_CQ | 0.840711 | 0.108809 | -1.3391 | 0.312441 | 0.201885 | No |
| 27 | ssRNA-40 | ssRNA-40_CQ | -1.14068 | 0.285049 | 1.946201 | 0.191026 | 0.145874 | No |
|  |  |  |  |  |  |  |  |  |
| **CD8+ CD69+** | **control** | **test** | **difference** | **Pvalue wilcoxon** | **statistic_paired students_t** | **pvalue_paired_students_t** | **q value** | **Discovery?** |
| 0 | K- | K-_CQ | 0.158931 | 1 | -0.36651 | 0.749127 | 0.786583 | No |
| 1 | Lipo | Lipo_CQ | 0.300857 | 0.108809 | -1.42374 | 0.290524 | 0.341656 | No |
| 2 | Lipo_miR-122 | Lipo_miR-122_CQ | -2.72774 | 0.108809 | 5.666985 | 0.029756 | 0.068531 | No |
| 3 | Lipo_miR-129 | Lipo_miR-129_CQ | -3.21039 | 0.108809 | 5.064164 | 0.036851 | 0.072228 | No |
| 4 | Lipo_miR-375 | Lipo_miR-375_CQ | -3.73965 | 0.108809 | 9.345761 | 0.011256 | 0.058712 | No |
| 5 | Lipo_miR-455 | Lipo_miR-455_CQ | -4.77154 | 0.108809 | 8.36893 | 0.013979 | 0.058712 | No |
| 6 | K- | Lipo_miR-122 | 3.587389 | 0.108809 | -5.57143 | 0.030738 | 0.068531 | No |
| 7 | K- | Lipo_miR-129 | 4.19463 | 0.108809 | -6.3851 | 0.023661 | 0.068531 | No |
| 8 | K- | Lipo_miR-375 | 7.215321 | 0.108809 | -10.8523 | 0.008384 | 0.058712 | No |
| 9 | K- | Lipo_miR-455 | 5.798314 | 0.108809 | -8.90155 | 0.012386 | 0.058712 | No |
| 10 | K- | Lipo_miR-122_CQ | 2.43669 | 0.108809 | -4.46842 | 0.04661 | 0.085646 | No |
| 11 | K- | Lipo_miR-129_CQ | 1.423248 | 0.108809 | -2.64458 | 0.11817 | 0.182853 | No |
| 12 | K- | Lipo_miR-375_CQ | 1.764999 | 0.108809 | -2.85994 | 0.103606 | 0.169223 | No |
| 13 | K- | Lipo_miR-455_CQ | 1.302976 | 0.108809 | -2.42506 | 0.136158 | 0.200152 | No |
| 14 | DOTAP | DOTAP_CQ | 0.190731 | 0.179712 | -1.88982 | 0.199359 | 0.266416 | No |
| 15 | DOTAP_miR-122 | DOTAP_miR-122_CQ | -3.3431 | 0.108809 | 10.29855 | 0.009297 | 0.058712 | No |
| 16 | DOTAP_miR-129 | DOTAP_miR-129_CQ | -2.56258 | 0.108809 | 6.041493 | 0.026321 | 0.068531 | No |
| 17 | DOTAP_miR-375 | DOTAP_miR-375_CQ | -2.81955 | 0.108809 | 3.459456 | 0.074358 | 0.128596 | No |
| 18 | DOTAP_miR-455 | DOTAP_miR-455_CQ | -4.2962 | 0.108809 | 8.869053 | 0.012476 | 0.058712 | No |
| 19 | K- | DOTAP_miR-122 | 4.535164 | 0.108809 | -7.36667 | 0.017933 | 0.065904 | No |
| 20 | K- | DOTAP_miR-129 | 3.43687 | 0.108809 | -5.39922 | 0.032634 | 0.068531 | No |
| 21 | K- | DOTAP_miR-375 | 4.273461 | 0.108809 | -6.17779 | 0.025215 | 0.068531 | No |
| 22 | K- | DOTAP_miR-455 | 11.72658 | 0.108809 | -19.005 | 0.002757 | 0.058712 | No |
| 23 | K- | DOTAP_miR-122_CQ | 0.652519 | 0.108809 | -1.14433 | 0.370971 | 0.403946 | No |
| 24 | K- | DOTAP_miR-129_CQ | 0.888635 | 0.108809 | -1.568 | 0.257417 | 0.322117 | No |
| 25 | K- | DOTAP_miR-375_CQ | 0.79322 | 0.108809 | -1.3467 | 0.310391 | 0.350981 | No |
| 26 | K- | DOTAP_miR-455_CQ | 0.916798 | 0.108809 | -1.54229 | 0.262953 | 0.322117 | No |
| 27 | ssRNA-40 | ssRNA-40_CQ | -1.16785 | 0.179712 | 1.922996 | 0.194398 | 0.266416 | No |

**Supplementary Table 1B: DOTAP/ miRNA and RNAiMAX Lipofectamine/miRNA effect on CD107a+ T-cell and NK activation (paired mean difference analysis).** Hedges' g correction with 5000 resamplings and 95% confidence interval was used to predict the effect of miRNA stimulation. Stimulation results with FDR q-value < 0.05 were considered as significant. DOTAP: DOTAP synthetic vesicles, DOTAP_miRNA: miRNA transfected with DOTAP synthetic vesicles; Lipo: RNAiMAX Lipofectamine synthetic vesicles, Lipo_miRNA: miRNA transfected with RNAiMAX Lipofectamine, K-: samples with only blood cells, miR: investigated miRNA alone; N=3.

| **CD8+ CD107a+** | **control** | **test** | **difference** | **Pvalue wilcoxon** | **statistic_paired students_t** | **pvalue_paired students_t** | **q value** | **Discovery?** |
| --- | --- | --- | --- | --- | --- | --- | --- | --- |
| 0 | K- | K-_CQ | 1.242772 | 0.108809 | -3.14286 | 0.088071 | 0.124044 | No |
| 1 | Lipo | Lipo_CQ | 0.123392 | 0.654721 | -0.1644 | 0.88453 | 0.928757 | No |
| 2 | Lipo_miR-122 | Lipo_miR-122_CQ | -1.49807 | 0.108809 | 2.160933 | 0.16326 | 0.199994 | No |
| 3 | Lipo_miR-129 | Lipo_miR-129_CQ | -2.39665 | 0.108809 | 4.703171 | 0.042357 | 0.11542 | No |
| 4 | Lipo_miR-375 | Lipo_miR-375_CQ | -2.92356 | 0.108809 | 6.308374 | 0.024219 | 0.107281 | No |
| 5 | Lipo_miR-455 | Lipo_miR-455_CQ | -2.8883 | 0.108809 | 4.541937 | 0.045213 | 0.11542 | No |
| 6 | K- | Lipo_miR-122 | 2.189204 | 0.108809 | -3.38997 | 0.07709 | 0.124044 | No |
| 7 | K- | Lipo_miR-129 | 3.08996 | 0.108809 | -4.52373 | 0.045553 | 0.11542 | No |
| 8 | K- | Lipo_miR-375 | 5.810739 | 0.108809 | -11.2444 | 0.007816 | 0.076597 | No |
| 9 | K- | Lipo_miR-455 | 3.2766 | 0.108809 | -5.5676 | 0.030778 | 0.113109 | No |
| 10 | K- | Lipo_miR-122_CQ | 3.411898 | 0.108809 | -13.8564 | 0.005168 | 0.07597 | No |
| 11 | K- | Lipo_miR-129_CQ | 1.996784 | 0.108809 | -3.90942 | 0.059637 | 0.124044 | No |
| 12 | K- | Lipo_miR-375_CQ | 2.848996 | 0.108809 | -4.34319 | 0.049138 | 0.11542 | No |
| 13 | K- | Lipo_miR-455_CQ | 2.529594 | 0.108809 | -3.21182 | 0.084791 | 0.124044 | No |
| 14 | DOTAP | DOTAP_CQ | 0.392543 | 0.59298 | -0.63311 | 0.591398 | 0.643967 | No |
| 15 | DOTAP_miR-122 | DOTAP_miR-122_CQ | -1.34987 | 0.108809 | 2.207463 | 0.157978 | 0.199994 | No |
| 16 | DOTAP_miR-129 | DOTAP_miR-129_CQ | -1.183 | 0.108809 | 1.834498 | 0.208015 | 0.235217 | No |
| 17 | DOTAP_miR-375 | DOTAP_miR-375_CQ | -3.90988 | 0.108809 | 7.7211 | 0.016364 | 0.107281 | No |
| 18 | DOTAP_miR-455 | DOTAP_miR-455_CQ | -2.17473 | 0.108809 | 6.252289 | 0.02464 | 0.107281 | No |
| 19 | K- | DOTAP_miR-122 | 1.815274 | 0.108809 | -3.09803 | 0.0903 | 0.124044 | No |
| 20 | K- | DOTAP_miR-129 | 1.717448 | 0.108809 | -3.04919 | 0.092822 | 0.124044 | No |
| 21 | K- | DOTAP_miR-375 | 7.377942 | 0.108809 | -14.6206 | 0.004646 | 0.07597 | No |
| 22 | K- | DOTAP_miR-455 | 3.293848 | 0.108809 | -6.13642 | 0.025543 | 0.107281 | No |
| 23 | K- | DOTAP_miR-122_CQ | 2.772167 | 0.108809 | -4.25525 | 0.051036 | 0.11542 | No |
| 24 | K- | DOTAP_miR-129_CQ | 2.66919 | 0.108809 | -3.35451 | 0.07854 | 0.124044 | No |
| 25 | K- | DOTAP_miR-375_CQ | 1.455356 | 0.108809 | -3.28571 | 0.081468 | 0.124044 | No |
| 26 | K- | DOTAP_miR-455_CQ | 0.947065 | 0.108809 | -2.02109 | 0.180663 | 0.21246 | No |
| 27 | ssRNA-40 | ssRNA-40_CQ | -2.30878 | 0.108809 | 3.300317 | 0.080834 | 0.124044 | No |
|  |  |  |  |  |  |  |  |  |
| **CD56+ CD107a+** | **control** | **test** | **difference** | **Pvalue wilcoxon** | **statistic_paired students_t** | **pvalue_paired students_t** | **q value** | **Discovery?** |
| 0 | K- | K-_CQ | 3.662033 | 0.108809 | -5.97284 | 0.026905 | 0.052984 | No |
| 1 | Lipo | Lipo_CQ | 2.778019 | 0.108809 | -4.71429 | 0.04217 | 0.057667 | No |
| 2 | Lipo_miR-122 | Lipo_miR-122_CQ | 1.075523 | 0.108809 | -4.69833 | 0.042439 | 0.057667 | No |
| 3 | Lipo_miR-129 | Lipo_miR-129_CQ | 0.292065 | 0.285049 | -0.73735 | 0.537683 | 0.443588 | No |
| 4 | Lipo_miR-375 | Lipo_miR-375_CQ | -0.73423 | 0.285049 | 0.999787 | 0.422732 | 0.40688 | No |
| 5 | Lipo_miR-455 | Lipo_miR-455_CQ | -1.31479 | 0.108809 | 1.820629 | 0.210263 | 0.220776 | No |
| 6 | K- | Lipo_miR-122 | 2.744463 | 0.108809 | -5.35046 | 0.033202 | 0.054783 | No |
| 7 | K- | Lipo_miR-129 | 4.882129 | 0.10247 | -83 | 0.000145 | 0.003257 | Yes |
| 8 | K- | Lipo_miR-375 | 5.946531 | 0.108809 | -7.68699 | 0.016506 | 0.047661 | Yes |
| 9 | K- | Lipo_miR-455 | 2.820592 | 0.108809 | -3.99961 | 0.057201 | 0.073408 | No |
| 10 | K- | Lipo_miR-122_CQ | 4.149456 | 0.108809 | -11.5899 | 0.007362 | 0.037318 | Yes |
| 11 | K- | Lipo_miR-129_CQ | 3.489971 | 0.108809 | -7.9588 | 0.015423 | 0.047661 | Yes |
| 12 | K- | Lipo_miR-375_CQ | 4.625903 | 0.108809 | -12.5426 | 0.006297 | 0.037318 | Yes |
| 13 | K- | Lipo_miR-455_CQ | 4.888464 | 0.10247 | -59.5 | 0.000282 | 0.003257 | Yes |
| 14 | DOTAP | DOTAP_CQ | 1.871795 | 0.108809 | -2.59242 | 0.122128 | 0.141058 | No |
| 15 | DOTAP_miR-122 | DOTAP_miR-122_CQ | 0.816314 | 0.285049 | -0.93109 | 0.450099 | 0.415891 | No |
| 16 | DOTAP_miR-129 | DOTAP_miR-129_CQ | 1.184938 | 0.108809 | -1.93549 | 0.192573 | 0.21183 | No |
| 17 | DOTAP_miR-375 | DOTAP_miR-375_CQ | 0.601426 | 0.59298 | -0.74573 | 0.533562 | 0.443588 | No |
| 18 | DOTAP_miR-455 | DOTAP_miR-455_CQ | 0.342712 | 0.285049 | -1.12542 | 0.377316 | 0.378957 | No |
| 19 | K- | DOTAP_miR-122 | 2.375886 | 0.108809 | -3.4841 | 0.073422 | 0.089266 | No |
| 20 | K- | DOTAP_miR-129 | 3.176325 | 0.108809 | -5.66074 | 0.029818 | 0.052984 | No |
| 21 | K- | DOTAP_miR-375 | 2.573073 | 0.108809 | -4.77319 | 0.041198 | 0.057667 | No |
| 22 | K- | DOTAP_miR-455 | 5.022702 | 0.108809 | -5.87436 | 0.027777 | 0.052984 | No |
| 23 | K- | DOTAP_miR-122_CQ | 4.483944 | 0.108809 | -10.0832 | 0.009693 | 0.037318 | Yes |
| 24 | K- | DOTAP_miR-129_CQ | 2.736304 | 0.108809 | -6.90215 | 0.020352 | 0.052237 | No |
| 25 | K- | DOTAP_miR-375_CQ | 6.025774 | 0.108809 | -10.53 | 0.008898 | 0.037318 | Yes |
| 26 | K- | DOTAP_miR-455_CQ | 4.300521 | 0.108809 | -5.99692 | 0.026698 | 0.052984 | No |
| 27 | ssRNA-40 | ssRNA-40_CQ | -0.30096 | 0.285049 | 0.783408 | 0.515429 | 0.443588 | No |

**Supplementary Table S2: DOTAP/ miRNA stimulation cytokine/chemokine release.** Kruskal-Wallis one-way ANOVA test was used to estimate cytokine/chemokine release after DOTAP/miRNA transfection (21 h after transfection) compared to negative control - DOTAP alone. The results were evaluated with FDR, cytokine/chemokine release with FDR<0.05 were considered as significant; N=3.

| Kruskal-Wallis test of TNF-alpha |  | |  | |  | |  | | |  | Kruskal-Wallis test of IL-6 | | |  | |  | | | |  | | |  | |  |
| --- | --- | --- | --- | --- | --- | --- | --- | --- | --- | --- | --- | --- | --- | --- | --- | --- | --- | --- | --- | --- | --- | --- | --- | --- | --- |
| Two-stage linear step-up procedure of Benjamini, Krieger and Yekutieli | Mean rank diff, | FDR Discovery? | | q value | | Individual P Value | |  | Two-stage linear step-up procedure of Benjamini, Krieger and Yekutieli | | | Mean rank diff, | | | FDR Discovery? | | q value | | Individual P Value | | |  |  |  |  |
| DOTAP vs. DOTAP-122 | -21 | Yes | | **0.0122** | | 0.0077 | |  | DOTAP vs. DOTAP-122 | | | -22 | | | Yes | | **0.0084** | | 0.0053 | | |  |  |  |  |
| DOTAP vs. DOTAP-122-CQ | -11 | No | | 0.1283 | | 0.1629 | |  | DOTAP vs. DOTAP-122-CQ | | | -11.83 | | | No | | 0.1204 | | 0.1338 | | |  |  |  |  |
| DOTAP vs. DOTAP-129 | -24.33 | Yes | | **0.0043** | | 0.002 | |  | DOTAP vs. DOTAP-129 | | | -24.67 | | | Yes | | **0.0037** | | 0.0018 | | |  |  |  |  |
| DOTAP vs. DOTAP-129-CQ | -16.67 | Yes | | **0.0435** | | 0.0345 | |  | DOTAP vs. DOTAP-129-CQ | | | -10.5 | | | No | | 0.1445 | | 0.1834 | | |  |  |  |  |
| DOTAP vs. DOTAP-193b | -4.5 | No | | 0.3579 | | 0.5681 | |  | DOTAP vs. DOTAP-193b | | | -6.5 | | | No | | 0.2872 | | 0.4102 | | |  |  |  |  |
| DOTAP vs. DOTAP-193b-CQ | -6.667 | No | | 0.2784 | | 0.3978 | |  | DOTAP vs. DOTAP-193b-CQ | | | -4.167 | | | No | | 0.3765 | | 0.5976 | | |  |  |  |  |
| DOTAP vs. DOTAP-375 | -28.67 | Yes | | **0.0012** | | 0.0003 | |  | DOTAP vs. DOTAP-375 | | | -27.33 | | | Yes | | **0.0017** | | 0.0005 | | |  |  |  |  |
| DOTAP vs. DOTAP-375-CQ | -11.17 | No | | 0.1283 | | 0.1567 | |  | DOTAP vs. DOTAP-375-CQ | | | -13.67 | | | No | | 0.0875 | | 0.0834 | | |  |  |  |  |
| DOTAP vs. DOTAP-455 | -28 | Yes | | **0.0012** | | 0.0004 | |  | DOTAP vs. DOTAP-455 | | | -28 | | | Yes | | **0.0017** | | 0.0004 | | |  |  |  |  |
| DOTAP vs. DOTAP-455-CQ | -13 | No | | 0.1041 | | 0.0992 | |  | DOTAP vs. DOTAP-455-CQ | | | -16.33 | | | Yes | | **0.0485** | | 0.0385 | | |  |  |  |  |
|  |  |  | |  | |  | |  |  | | |  | | |  | |  | |  | | |  |  |  |  |
| Kruskal-Wallis test of INF-alpha |  |  | |  | |  | |  | Kruskal-Wallis test of IL-8 | | |  | | |  | |  | |  | | |  |  |  |  |
| Two-stage linear step-up procedure of Benjamini, Krieger and Yekutieli | Mean rank diff, | FDR Discovery? | | q value | | Individual P Value | |  | Two-stage linear step-up procedure of Benjamini, Krieger and Yekutieli | | | Mean rank diff, | | | FDR Discovery? | | q value | | Individual P Value | | |  |  |  |  |
| DOTAP vs. DOTAP-122 | -27.33 | Yes | | **0.0019** | | 0.0005 | |  | DOTAP vs. DOTAP-122 | | | -8.667 | | | No | | 0.2859 | | 0.2723 | | |  |  |  |  |
| DOTAP vs. DOTAP-122-CQ | -6.833 | No | | 0.2835 | | 0.3857 | |  | DOTAP vs. DOTAP-122-CQ | | | -15 | | | No | | 0.1086 | | 0.0574 | | |  |  |  |  |
| DOTAP vs. DOTAP-129 | -18.33 | Yes | | **0.0367** | | 0.0199 | |  | DOTAP vs. DOTAP-129 | | | -24.67 | | | Yes | | **0.0168** | | 0.0018 | | |  |  |  |  |
| DOTAP vs. DOTAP-129-CQ | -14.67 | No | | 0.0883 | | 0.0626 | |  | DOTAP vs. DOTAP-129-CQ | | | -13 | | | No | | 0.1569 | | 0.0996 | | |  |  |  |  |
| DOTAP vs. DOTAP-193b | -9.167 | No | | 0.1997 | | 0.2446 | |  | DOTAP vs. DOTAP-193b | | | -8.667 | | | No | | 0.2859 | | 0.2723 | | |  |  |  |  |
| DOTAP vs. DOTAP-193b-CQ | -14.17 | No | | 0.0883 | | 0.0721 | |  | DOTAP vs. DOTAP-193b-CQ | | | 2 | | | No | | 0.756 | | 0.8 | | |  |  |  |  |
| DOTAP vs. DOTAP-375 | -28.67 | Yes | | **0.0019** | | 0.0003 | |  | DOTAP vs. DOTAP-375 | | | -16.33 | | | No | | 0.1086 | | 0.0386 | | |  |  |  |  |
| DOTAP vs. DOTAP-375-CQ | -10.83 | No | | 0.1775 | | 0.1691 | |  | DOTAP vs. DOTAP-375-CQ | | | -11 | | | No | | 0.2208 | | 0.1635 | | |  |  |  |  |
| DOTAP vs. DOTAP-455 | -25 | Yes | | **0.0037** | | 0.0015 | |  | DOTAP vs. DOTAP-455 | | | -15.33 | | | No | | 0.1086 | | 0.0521 | | |  |  |  |  |
| DOTAP vs. DOTAP-455-CQ | -10 | No | | 0.1877 | | 0.2043 | |  | DOTAP vs. DOTAP-455-CQ | | | -17.67 | | | No | | 0.1086 | | 0.0252 | | |  |  |  |  |
|  |  |  | |  | |  | |  |  | | |  | | |  | |  | |  | | |  |  |  |  |
| Kruskal-Wallis test of INF-gamma |  |  | |  | |  | | Kruskal-Wallis test of IL-10 | | | | |  | | | | |  | | |  | | |  | |
| Two-stage linear step-up procedure of Benjamini, Krieger and Yekutieli | Mean rank diff, | FDR Discovery? | | q value | | Individual P Value | |  | Two-stage linear step-up procedure of Benjamini, Krieger and Yekutieli | | | Mean rank diff, | | | FDR Discovery? | | q value | | Individual P Value | | |  |  |  |  |
| DOTAP vs. DOTAP-122 | -21 | Yes | | **0.0121** | | 0.0077 | |  | DOTAP vs. DOTAP-122 | | | -19 | | | Yes | | **0.0169** | | 0.0161 | | |  |  |  |  |
| DOTAP vs. DOTAP-122-CQ | -7.5 | No | | 0.2359 | | 0.3413 | |  | DOTAP vs. DOTAP-122-CQ | | | -10 | | | No | | 0.1397 | | 0.2053 | | |  |  |  |  |
| DOTAP vs. DOTAP-129 | -25 | Yes | | **0.0032** | | 0.0015 | |  | DOTAP vs. DOTAP-129 | | | -23.33 | | | Yes | | **0.0055** | | 0.0031 | | |  |  |  |  |
| DOTAP vs. DOTAP-129-CQ | -13.33 | No | | 0.0952 | | 0.0907 | |  | DOTAP vs. DOTAP-129-CQ | | | -9.833 | | | No | | 0.1397 | | 0.2129 | | |  |  |  |  |
| DOTAP vs. DOTAP-193b | -10.17 | No | | 0.1552 | | 0.1971 | |  | DOTAP vs. DOTAP-193b | | | -5.333 | | | No | | 0.2837 | | 0.4993 | | |  |  |  |  |
| DOTAP vs. DOTAP-193b-CQ | -7 | No | | 0.2359 | | 0.3744 | |  | DOTAP vs. DOTAP-193b-CQ | | | -4.833 | | | No | | 0.2837 | | 0.5404 | | |  |  |  |  |
| DOTAP vs. DOTAP-375 | -27 | Yes | | **0.0019** | | 0.0006 | |  | DOTAP vs. DOTAP-375 | | | -29.33 | | | Yes | | **0.0011** | | 0.0002 | | |  |  |  |  |
| DOTAP vs. DOTAP-375-CQ | -11 | No | | 0.1465 | | 0.1628 | |  | DOTAP vs. DOTAP-375-CQ | | | -16 | | | Yes | | **0.0374** | | 0.0427 | | |  |  |  |  |
| DOTAP vs. DOTAP-455 | -29 | Yes | | **0.0015** | | 0.0002 | |  | DOTAP vs. DOTAP-455 | | | -27.67 | | | Yes | | **0.0012** | | 0.0005 | | |  |  |  |  |
| DOTAP vs. DOTAP-455-CQ | -14 | No | | 0.0952 | | 0.0757 | |  | DOTAP vs. DOTAP-455-CQ | | | -19.67 | | | Yes | | **0.0167** | | 0.0127 | | |  |  |  |  |
|  |  |  | |  | |  | |  |  | | |  | | |  | |  | |  | | |  |  |  |  |
| Kruskal-Wallis test of IL-1beta |  |  | |  | |  | |  | Kruskal-Wallis test of MCP-1 | | |  | | |  | |  | |  | | |  |  |  |  |
| Two-stage linear step-up procedure of Benjamini, Krieger and Yekutieli | Mean rank diff, | FDR Discovery? | | q value | | Individual P Value | |  | Two-stage linear step-up procedure of Benjamini, Krieger and Yekutieli | | | Mean rank diff, | | | FDR Discovery? | | q value | | Individual P Value | | |  |  |  |  |
| DOTAP vs. DOTAP-122 | -21 | Yes | | **0.0122** | | 0.0077 | |  | DOTAP vs. DOTAP-122 | | | -21.67 | | | Yes | | **0.0096** | | 0.0061 | | |  |  |  |  |
| DOTAP vs. DOTAP-122-CQ | -7.833 | No | | 0.2242 | | 0.3203 | |  | DOTAP vs. DOTAP-122-CQ | | | -12 | | | No | | 0.1157 | | 0.1285 | | |  |  |  |  |
| DOTAP vs. DOTAP-129 | -24.67 | Yes | | **0.0037** | | 0.0018 | |  | DOTAP vs. DOTAP-129 | | | -25.67 | | | Yes | | **0.0042** | | 0.0012 | | |  |  |  |  |
| DOTAP vs. DOTAP-129-CQ | -15.33 | No | | 0.0652 | | 0.0517 | |  | DOTAP vs. DOTAP-129-CQ | | | -8.333 | | | No | | 0.2293 | | 0.2912 | | |  |  |  |  |
| DOTAP vs. DOTAP-193b | -5.167 | No | | 0.3227 | | 0.5122 | |  | DOTAP vs. DOTAP-193b | | | -4.333 | | | No | | 0.4082 | | 0.5831 | | |  |  |  |  |
| DOTAP vs. DOTAP-193b-CQ | -9.833 | No | | 0.1671 | | 0.2122 | |  | DOTAP vs. DOTAP-193b-CQ | | | -3.333 | | | No | | 0.4239 | | 0.6729 | | |  |  |  |  |
| DOTAP vs. DOTAP-375 | -28.33 | Yes | | **0.0012** | | 0.0003 | |  | DOTAP vs. DOTAP-375 | | | -17.67 | | | Yes | | **0.0318** | | 0.0252 | | |  |  |  |  |
| DOTAP vs. DOTAP-375-CQ | -10.5 | No | | 0.1646 | | 0.1828 | |  | DOTAP vs. DOTAP-375-CQ | | | -25.33 | | | Yes | | **0.0042** | | 0.0013 | | |  |  |  |  |
| DOTAP vs. DOTAP-455 | -28 | Yes | | **0.0012** | | 0.0004 | |  | DOTAP vs. DOTAP-455 | | | -15.67 | | | Yes | | **0.0496** | | 0.0472 | | |  |  |  |  |
| DOTAP vs. DOTAP-455-CQ | -14.33 | No | | 0.0725 | | 0.069 | |  | DOTAP vs. DOTAP-455-CQ | | | -23.67 | | | Yes | | **0.0057** | | 0.0027 | | |  |  |  |  |

Gating strategy: T-cell CD4+ and CD8+ CD69+ expression. Sample: DOTAP (negative control; 21h after the stimulation).


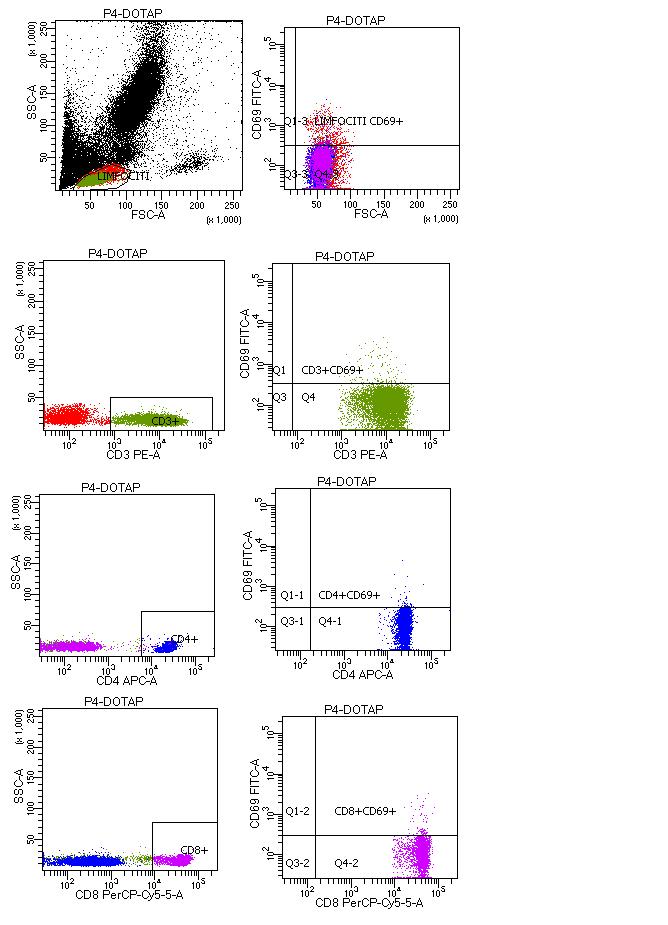


LYMPHOCYTES CD69+


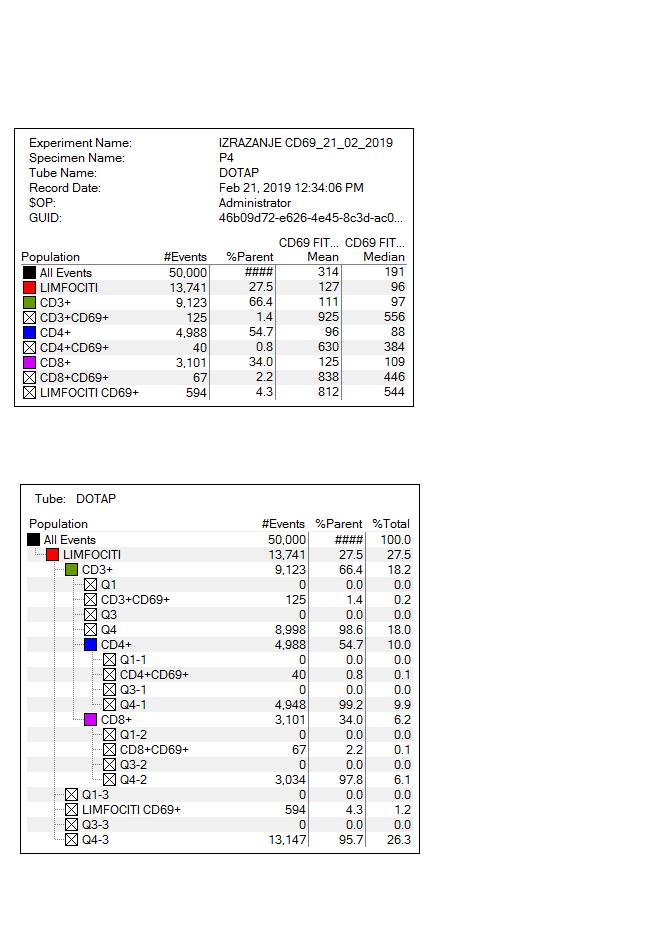


LYMPHOCYTES


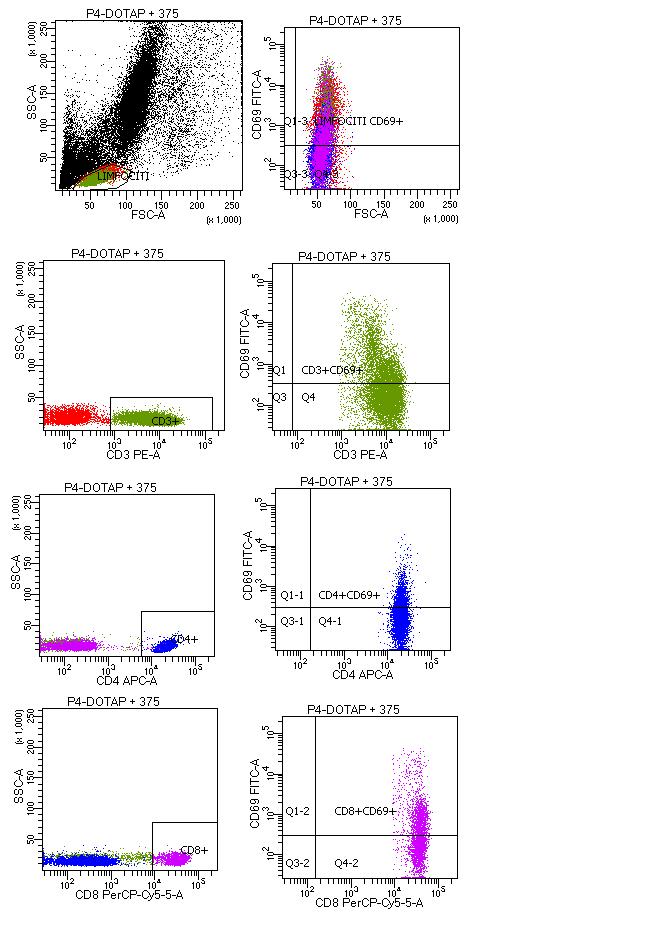
Gating strategy: T-cell CD4+ and CD8+ CD69+ expression. Sample: DOTAP/hsa-miR-375-3p (21h after the stimulation).

LYMPHOCYTES CD69+


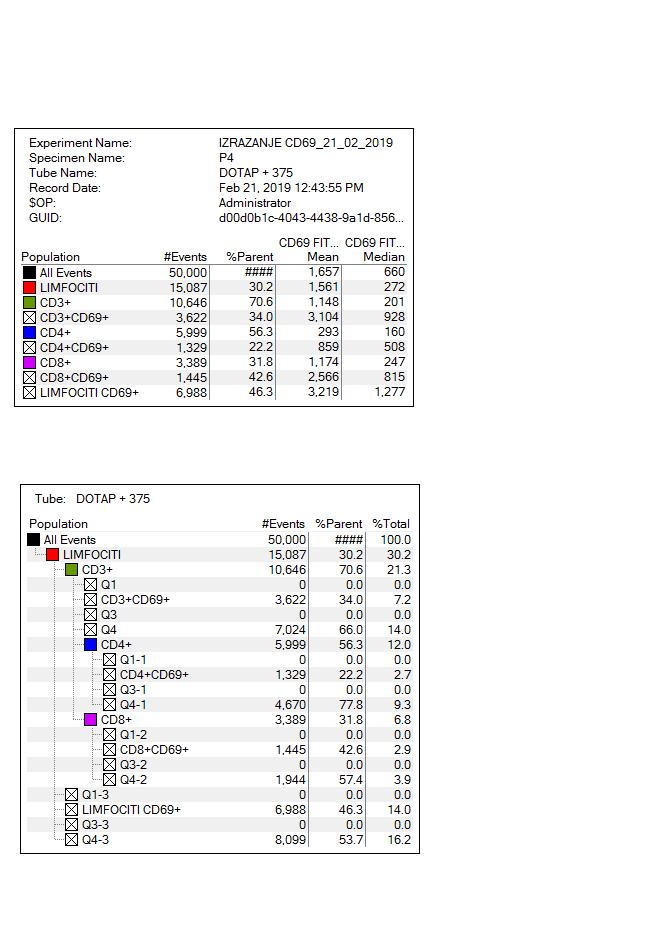


LYMPHOCYTES

Gating strategy: CD8+ T-cell and CD56+ NK-cell CD107a expression. Sample: DOTAP (negative control; 21h after the stimulation) [hsa-miR-375-3p-FAM: FITC absorbance/emission spectrum].


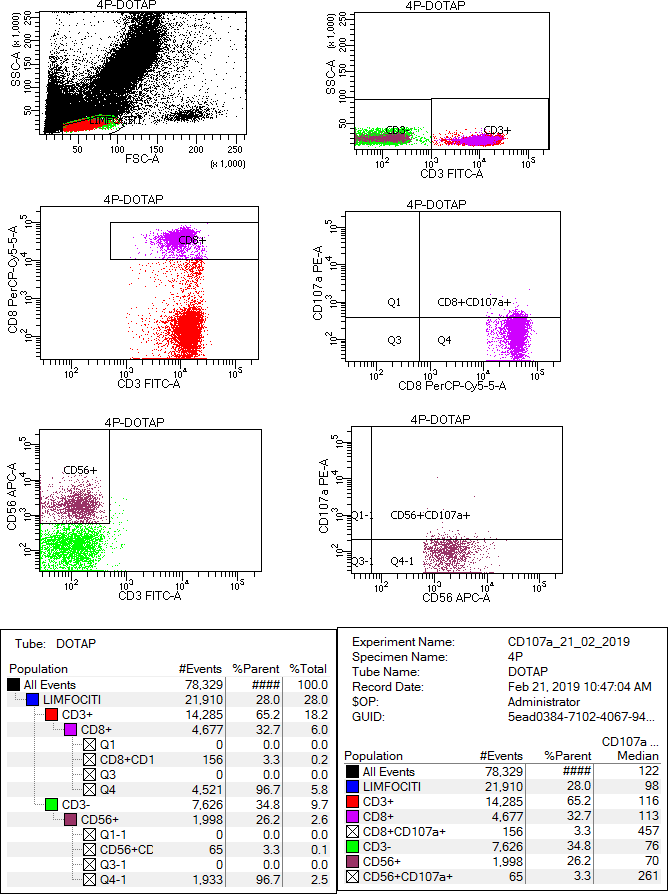

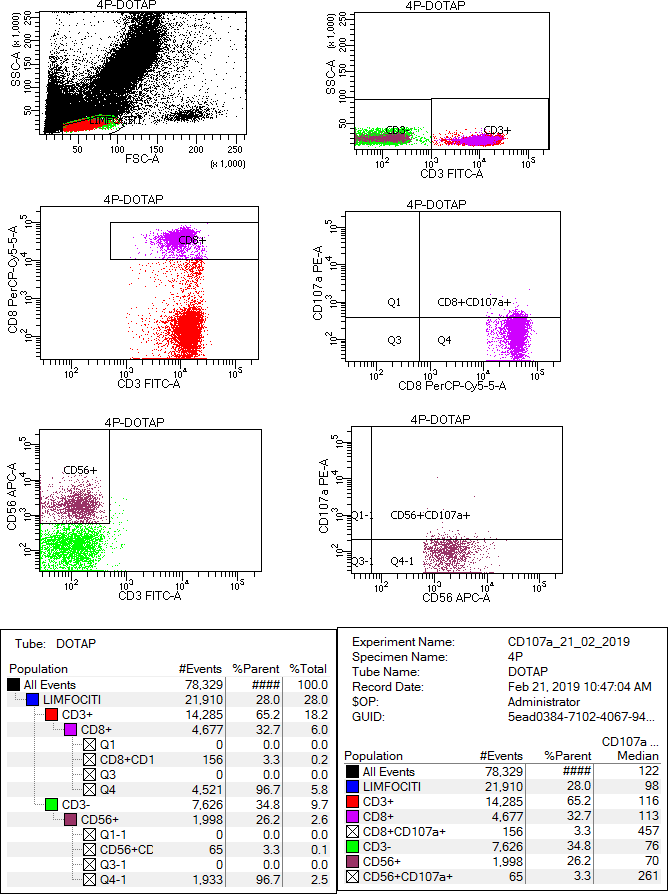


LYMPHOCYTES

CD8+ CD107a+

CD56+ CD107a+

Gating strategy: T-cell and CD56+ NK-cell CD107a expression. Sample: DOTAP/hsa-miR-375-3p (21h after the stimulation) [hsa-miR-375-3p-FAM: FITC absorbance/emission spectrum].


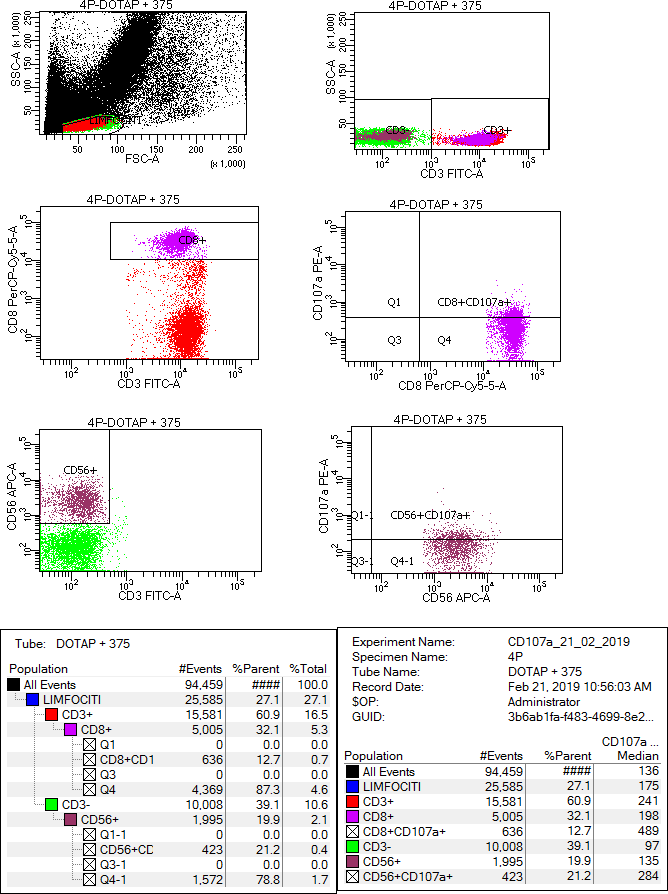

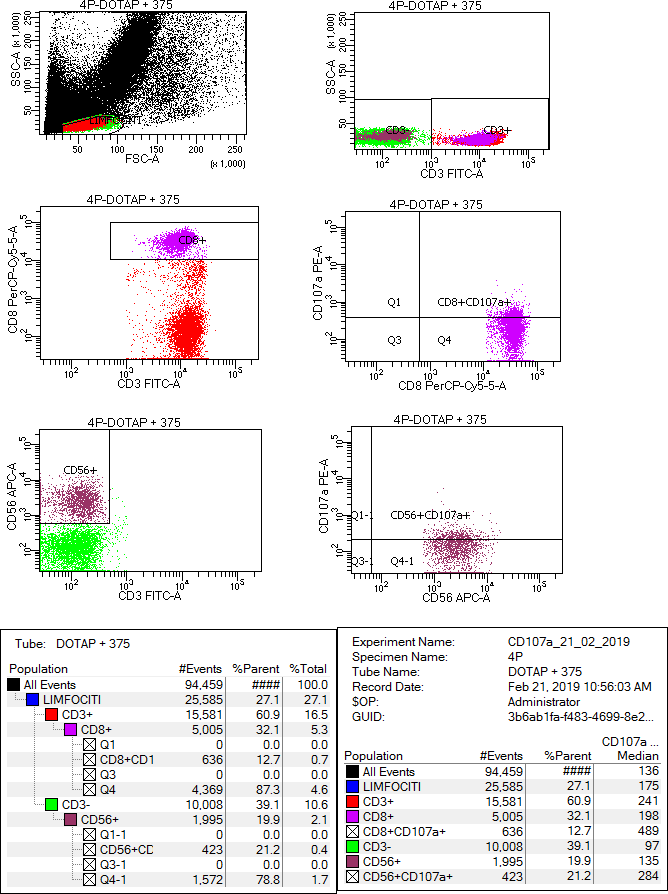


LYMPHOCYTES

CD56+ CD107a+

CD8+ CD107a+

Source data Supplementary Figure 1: Effect of miRNA concentration on CD69+ transition and CD107a degranulation (21h after the stimulation).

|  | CD4+ CD69+ |  | CD8+ CD69+ |  | CD8+CD107a+ |  |
| --- | --- | --- | --- | --- | --- | --- |
| miR concentration [µM] | SampleA | SampleB | SampleA | SampleB | SampleA | SampleB |
| 0.00 | 1.1 | 1.1 | 2.2 | 2.0 | 0.5 | 0.3 |
| 0.10 | 6.1 | 17.9 | 18.3 | 26.5 | 3.5 | 5.1 |
| 0.25 | 16.1 | 25.8 | 25.7 | 31.9 | 4.4 | 4.6 |
| 0.50 | 12.9 | 23.8 | 21.8 | 30.8 | 4.1 | 4.7 |
| 0.75 | 12.2 | 22.4 | 21.4 | 29.2 | 3.9 | 4.3 |
| 1.00 | 11.8 | 21.5 | 20.5 | 29.8 | 2.9 | 3.9 |

| **CD4+CD69+** | FMO | K- | K+ (IL2+ PHA) | TLR3 | TLR7/8 | DOTAP | DOTAP+miR-122 | DOTAP+miR-192 | DOTAP+miR-193b | DOTAP+miR-185 | DOTAP+miR-195 | DOTAP+miR-455 | DOTAP+miR-375 | DOTAP+miR-129 | DOTAP+ RNA41 | miR-122 | miR-192 | miR-193b | miR-185 | miR-195 | miR-455 | miR-375 | miR-129 | RNA41 |
| --- | --- | --- | --- | --- | --- | --- | --- | --- | --- | --- | --- | --- | --- | --- | --- | --- | --- | --- | --- | --- | --- | --- | --- | --- |
| P1 | 0.5 | 0.7 | - | 0.9 | 13.7 | 0.5 | 7 | 4.6 | 0.3 | 6.9 | 12.8 | 19 | 11.6 | 5.1 | 0.4 | 0.4 | 0.3 | 0.3 | 0.5 | 0.4 | 0.4 | 0.3 | 0.3 | 0.3 |
| P2 | 0.3 | 2.2 | 75.6 | 1.2 | 7.4 | 0.7 | 10.6 | 5.9 | 1 | 13.4 | 13.1 | 17.1 | 14.7 | 6 | 1.2 | 1 | 0.8 | 0.9 | 0.9 | 0.9 | 0.7 | 0.7 | 1 | 0.9 |
| P3 | 0.6 | 1.2 | - | 1.6 | 4.9 | 0.9 | 8.5 | 5.1 | 1.6 | 8.8 | 9.5 | 11.5 | 9.2 | 6.1 | 1.4 | 1.3 | 1.8 | 1.4 | 1.7 | 1.6 | 1.3 | 1.5 | 1.2 | 1.1 |
| P4 | 0.4 | 1.4 | 73.8 | 2.3 | 11.3 | 1.3 | 14.9 | 7.4 | 1.5 | 21.3 | 24.4 | 28.6 | 24.5 | 14.2 | 1.3 | 1.9 | 1.8 | 2 | 1.9 | 2.2 | 1.6 | 2 | 1.4 | 1.8 |
| P5 | 0.5 | 1.1 | 84 | 2.6 | 21.6 | 1.5 | 33 | 19.5 | 2.1 | 38.8 | 40.5 | 42 | 39.3 | 17.4 | 1.2 | 1.2 | 1.5 | 1.6 | 1.4 | 1.2 | 1.4 | 1.6 | 1.5 | 1.5 |
| P6 | 0.8 | 2.7 | 48.6 | 6.2 | 5.6 | 3 | 13.2 | 9.9 | 2.4 | 15.1 | 18.4 | 19.3 | 22.6 | 13.9 | 1.4 | 1.3 | 1.1 | 1.2 | 1 | 1.2 | 1.9 | 2.5 | 3.2 | 2.7 |
| P7 | 0.3 | 1.2 | 68.5 | 1.1 | 6.1 | 0.6 | 12.3 | 7.6 | 0.6 | 14.6 | 15.9 | 14.9 | 20.6 | 9.6 | 1 | - | - | - | - | - | 1.1 | - | - | - |
| P8 | 0.5 | 0.9 | 69.4 | 5.3 | 18.6 | 0.6 | 14 | 12.2 | 0.7 | 18.1 | 20.5 | 23 | 20.6 | 11.2 | 0.4 | - | - | - | - | - | 0.5 | - | - | - |
| P9 | 0.1 | 2.1 | 72.5 | 4.1 | 17.2 | 2.3 | 15.2 | 7.9 | 1.9 | 16.8 | 9.8 | 21.3 | 10.3 | 8.4 | 1.8 | - | - | - | - | - | 2.4 | - | - | - |
| P10 | 0.3 | 1.3 | 62.2 | 2.7 | 18.1 | 1.8 | 10.6 | 12.8 | 1.2 | 13.1 | 12.4 | 24.7 | 14 | 13.5 | 1.4 | - | - | - | - | - | 2 | - | - | - |
| N1 | 0.6 | 1.7 | 79.4 | 5 | 17.2 | 0.9 | 18.6 | 11.7 | 1.7 | 23.6 | 30.2 | 28.9 | 32.6 | 20.3 | 2.2 | 1.5 | 1.3 | 1.7 | 1.9 | 1.6 | 1.8 | 1.3 | 2.2 | 1.1 |
| N2 | 0.7 | 1.3 | 76.7 | 3.1 | 23.6 | 1.3 | 20.3 | 10.8 | 0.8 | 23.4 | 29.1 | 30.9 | 32.4 | 15.6 | 1.3 | 1.4 | 0.9 | 0.9 | 1.6 | 2 | 1.9 | 1.3 | 1.2 | 1.8 |
| N3 | 0.7 | 4.3 | 74.4 | 4.9 | 15.1 | 4.6 | 33 | 21.4 | 4.6 | 32.9 | 27.2 | 32.3 | 31.6 | 18.2 | 5.4 | 4.1 | 4.5 | 4.5 | 4.5 | 4.5 | 4.3 | 4.7 | 5.2 | 4.4 |
| N4 | 0.7 | 1.9 | 78.3 | 5.8 | 16.8 | 1.8 | 23.7 | 11.8 | 1.8 | 27 | 31.3 | 37.3 | 34.3 | 20.9 | 1.9 | 2 | 1.9 | 1.9 | 2.1 | 1.7 | 1.9 | 1.7 | 1.7 | 1.7 |
| N5 | 0.6 | 1.2 | 56.1 | 1.9 | 6.5 | 2.4 | 6.8 | 3.8 | 1.9 | 11.6 | 12.3 | 16.3 | 15.9 | 8.2 | 2.3 | 2.5 | 1.9 | 1.9 | 2 | 1.7 | 2.1 | 2.1 | 2.1 | 2.1 |
| N6 | 0.4 | 1.5 | 73.1 | 2.7 | 15.6 | 0.7 | 26.4 | 23.2 | 0.8 | 32.3 | 37.2 | 37.6 | 35.5 | 24.3 | 1.6 | - | - | - | - | - | 0.9 | - | - | - |
| N7 | 0.7 | 1.7 | 61.7 | 4.1 | 20.1 | 1.9 | 23.3 | 20.1 | 1.8 | 30.9 | 28.7 | 30.5 | 30.4 | 16.1 | 2.2 | - | - | - | - | - | 2.2 | - | - | - |
| N8 | 0.5 | 2.2 | 72.5 | 2.4 | 17.8 | 3.2 | 20.2 | 9.4 | 1.8 | 18.8 | 8.5 | 32 | 20.9 | 14.5 | 2.2 | - | - | - | - | - | 2.2 | - | - | - |
| N9 | 0.5 | 1.7 | 62.4 | 1.7 | 11.6 | 1.2 | 16.9 | 1.6 | 1.1 | 13.1 | 15.4 | 22.2 | 16.7 | 10.7 | 1.4 | - | - | - | - | - | 1.2 | - | - | - |
| N10 | 0.5 | 1 | 63.3 | 1.8 | 19.7 | 1.6 | 29.4 | 30 | 1.7 | 26.6 | 26.2 | 34.6 | 30.6 | 28 | 1.7 | - | - | - | - | - | 1.3 | - | - | - |
|  |  |  |  |  |  |  |  |  |  |  |  |  |  |  |  |  |  |  |  |  |  |  |  |  |
|  |  |  |  |  |  |  |  |  |  |  |  |  |  |  |  |  |  |  |  |  |  |  |  |  |
| **CD8+ CD69+** | FMO | K- | K+ (IL2+ PHA) | TLR3 | TLR7/8 | DOTAP | DOTAP+miR-122 | DOTAP+miR-192 | DOTAP+miR-193b | DOTAP+miR-185 | DOTAP+miR-195 | DOTAP+miR-455 | DOTAP+miR-375 | DOTAP+miR-129 | DOTAP+ RNA41 | miR-122 | miR-192 | miR-193b | miR-185 | miR-195 | miR-455 | miR-375 | miR-129 | RNA41 |
| P1 | 0.6 | 0.7 | - | 4.1 | 17.9 | 1.1 | 11.9 | 11.2 | 0.6 | 12.5 | 16.7 | 19.7 | 15.1 | 12 | 0.9 | 0.4 | 0.5 | 0.7 | 0.7 | 0.5 | 0.4 | 0.3 | 0.5 | 0.4 |
| P2 | 0.4 | 3 | 75.6 | 2.6 | 13.3 | 1.6 | 18.4 | 12.4 | 1.4 | 17.9 | 20.4 | 27.1 | 19.7 | 11.4 | 1.7 | 1.5 | 1.7 | 1.6 | 1.7 | 1.2 | 1.6 | 1.4 | 1.2 | 1.3 |
| P3 | 0.5 | 1.8 | - | 5.4 | 19.9 | 1.9 | 23.4 | 20.6 | 2.5 | 25.4 | 25.8 | 25.4 | 23.9 | 20.9 | 1.8 | 1.9 | 1.7 | 1.8 | 1.2 | 1.6 | 1.8 | 1.7 | 1.9 | 1.3 |
| P4 | 0.7 | 3 | 73.8 | 9.2 | 31.4 | 2.5 | 35.9 | 27.2 | 2.8 | 42.5 | 45.3 | 48.7 | 45.2 | 35.6 | 3.1 | 4.1 | 3.2 | 3.1 | 3.6 | 3.2 | 3.2 | 3.7 | 3.2 | 3.5 |
| P5 | 0.6 | 1.6 | 84 | 3.9 | 26.9 | 2.2 | 38.6 | 29.4 | 2.2 | 45.9 | 47.6 | 48.9 | 46.3 | 25.1 | 2.1 | 1.5 | 2.5 | 2.2 | 2.5 | 2 | 2.1 | 1.9 | 2 | 1.8 |
| P6 | 0.8 | 3.7 | 48.6 | 10.7 | 10.8 | 3.4 | 19 | 14.5 | 3.8 | 20 | 22.6 | 23.1 | 24.7 | 16.5 | 2.4 | 2.2 | 2.1 | 2.2 | 2 | 2.4 | 2.8 | 3.2 | 3 | 3.1 |
| P7 | 0.6 | 4.5 | 68.5 | 6.4 | 14.4 | 4.3 | 23.4 | 17.8 | 2.7 | 25.1 | 23.9 | 27.4 | 28.3 | 19.4 | 4.1 | - | - | - | - | - | 3.8 | - | - | - |
| P8 | 1.3 | 4 | 69.4 | 14.6 | 29.1 | 2.2 | 25.4 | 23.3 | 1.9 | 29 | 31.3 | 32 | 27.9 | 24.1 | 2.1 | - | - | - | - | - | 1.9 | - | - | - |
| P9 | 0.5 | 4.3 | 72.5 | 12.7 | 44.6 | 5.2 | 41 | 22.9 | 4.1 | 44.6 | 31 | 52.1 | 32.3 | 29.8 | 3.3 | - | - | - | - | - | 4.1 | - | - | - |
| P10 | 0.2 | 2 | 62.2 | 5.9 | 24.5 | 2.4 | 20.7 | 18.1 | 1.6 | 23.3 | 23.5 | 29.7 | 22.3 | 22.4 | 2.4 | - | - | - | - | - | 2.7 | - | - | - |
| N1 | 0.4 | 2.1 | 79.4 | 14.1 | 25.4 | 1.6 | 27.3 | 23.1 | 2.5 | 33.1 | 37 | 37.3 | 38 | 30.6 | 2.7 | 2.6 | 1.9 | 1.8 | 2.4 | 2.1 | 2.2 | 2.2 | 3 | 1.8 |
| N2 | 0.3 | 1.9 | 76.7 | 8.2 | 32.7 | 1.5 | 30.5 | 19.3 | 1.5 | 34.2 | 40.7 | 40 | 42.7 | 25.4 | 1.9 | 1.2 | 0.9 | 1.5 | 1.5 | 1.7 | 1.8 | 1.6 | 1 | 2.2 |
| N3 | 0.9 | 8.5 | 74.4 | 13.6 | 27.8 | 9.6 | 42.6 | 35.1 | 10 | 43.3 | 38.9 | 41.3 | 41.9 | 31.6 | 11.6 | 9.1 | 8.8 | 10.5 | 9.7 | 7 | 8.1 | 7.9 | 8.1 | 7.9 |
| N4 | 0.7 | 2.4 | 78.3 | 13.8 | 31.9 | 2.5 | 39.7 | 29.5 | 2.5 | 44.7 | 49.5 | 53.1 | 50.4 | 39.5 | 2.5 | 2.5 | 2.2 | 2 | 2.3 | 2.6 | 1.9 | 1.9 | 2.2 | 2.4 |
| N5 | 0.5 | 1.3 | 56.1 | 7.5 | 15.3 | 1.6 | 18.4 | 12.2 | 1.6 | 20.5 | 23.3 | 22.4 | 21.7 | 17.3 | 2.2 | 1.9 | 1.8 | 1.8 | 1.8 | 1.9 | 2 | 2 | 1.9 | 1.8 |
| N6 | 0.4 | 2.5 | 73.1 | 8.1 | 20.4 | 1.7 | 28.7 | 27.7 | 2.1 | 32.6 | 38.4 | 36.9 | 34.4 | 27.6 | 3.3 | - | - | - | - | - | 1.7 | - | - | - |
| N7 | 0.9 | 1.5 | 61.7 | 10.3 | 29.9 | 2 | 32.3 | 30.3 | 1.8 | 40.6 | 38.1 | 37.1 | 40 | 26 | 2.5 | - | - | - | - | - | 2 | - | - | - |
| N8 | 0.8 | 3.8 | 72.5 | 6.6 | 32.9 | 6.2 | 31.4 | 18.7 | 3.5 | 30.9 | 18.2 | 44.6 | 30.1 | 28.1 | 3.6 | - | - | - | - | - | 3.1 | - | - | - |
| N9 | 0.4 | 1.1 | 62.4 | 3 | 21.2 | 1.1 | 25.3 | 1.3 | 1.3 | 21.8 | 23.8 | 28 | 25.5 | 18.6 | 1.2 | - | - | - | - | - | 1.4 | - | - | - |
| N10 | 0.3 | 1.4 | 63.3 | 3 | 15.3 | 1.9 | 21.9 | 21.8 | 1.9 | 21.2 | 23.1 | 27.3 | 24.1 | 21 | 2 | - | - | - | - | - | 1.7 | - | - | - |

Source data (Figures 3, 4; Tables 5 A, 6 A): CD4+ and CD8+ T-cell CD69+ expression flow cytometry results 21h after the stimulation with DOTAP/miRNA.

| **CD8+ CD107a+** | FMO | K+(IL2+PHA) | K+ (IL2+ PHA) | TLR3 | TLR7/8 | DOTAP | DOTAP+miR-122 | DOTAP+miR-192 | DOTAP+miR-193b | DOTAP+miR-185 | DOTAP+miR-195 | DOTAP+miR-455 | DOTAP+miR-375 | DOTAP+miR-129 | DOTAP+ RNA41 | miR-122 | miR-192 | miR-193b | miR-185 | miR-195 | miR-455 | miR-375 | miR-129 | RNA41 |
| --- | --- | --- | --- | --- | --- | --- | --- | --- | --- | --- | --- | --- | --- | --- | --- | --- | --- | --- | --- | --- | --- | --- | --- | --- |
| P1 | 0.4 | 1.4 |  | 4.5 | 8.6 | 2 | 7 | 6 | 1.3 | 7.5 | 10.9 | 14.2 | 9.8 | 7.8 | 1.4 | 1.3 | 1.5 | 1.4 | 1.6 | 1.5 | 1.7 | 1.2 | 1.4 | 1.3 |
| P2 | 0.9 | 2.5 | 16.2 | 2.4 | 6.5 | 1.7 | 5.8 | 4.6 | 1.8 | 5.7 | 6.4 | 9.4 | 6.8 | 5.1 | 1.9 | 1.9 | 1.2 | 1.5 | 1.8 | 1.1 | 2.1 | 2 | 2.3 | 2 |
| P3 | 1 | 2.6 |  | 2.9 | 7.8 | 3 | 7 | 4.7 | 1.7 | 6.5 | 7.2 | 10.2 | 8 | 6.1 | 2.7 | 1.9 | 2.3 | 1.9 | 1.7 | 2.1 | 2.4 | 2.3 | 2 | 2.2 |
| P4 | 0.7 | 2.5 | 18.2 | 4.9 | 12.1 | 2.7 | 8.8 | 7.3 | 2.6 | 10 | 10.1 | 14.4 | 11.4 | 8.3 | 2.1 | 2.3 | 2.5 | 2.6 | 2.2 | 2 | 1.4 | 1.9 | 2.2 | 2.6 |
| P5 | 0.7 | 4.2 | 35.4 | 9 | 25.3 | 4.8 | 29.9 | 21 | 4.3 | 30 | 31.4 | 32.2 | 30.1 | 14.5 | 3.9 | 4.6 | 4.3 | 3.8 | 3.4 | 3.6 | 3.2 | 3.5 | 3.2 | 3.6 |
| P6 | 0.5 | 1.7 | 12.2 | 3.1 | 4.8 | 2.1 | 4.7 | 4.3 | 1.4 | 3.9 | 4.2 | 4.4 | 5.1 | 4 | 1.4 | 1.3 | 1.3 | 1.4 | 1.3 | 1.2 | 1.3 | 1.4 | 1.5 | 1.7 |
| P7 | 0.5 | 1.6 | 12.1 | 2.7 | 5.4 | 2.1 | 8.7 | 2 | 4.7 | 7.6 | 6.9 | 5.8 | 10 | 5.2 | 1.7 | - | - | - | - | - | 1.3 | - | - | - |
| P8 | 0.3 | 0.8 | 11.1 | 3.4 | 4.9 | 1.2 | 4.7 | 4.2 | 1.1 | 4.9 | 5.7 | 8.8 | 5.6 | 4.4 | 0.9 | - | - | - | - | - | 1 | - | - | - |
| P9 | 0.3 | 2.6 | 14.5 | 2.7 | 4.8 | 2 | 4.4 | 4.7 | 3.1 | 6.5 | 5.1 | 8.5 | 5.5 | 4.8 | 3 | - | - | - | - | - | 3.5 | - | - | - |
| P10 | 0.8 | 2.9 | 15.4 | 4.5 | 9.4 | 2.5 | 8.9 | 6.4 | 2.6 | 10 | 8.9 | 14.5 | 8.4 | 7.5 | 2.8 | - | - | - | - | - | 2.8 | - | - | - |
| N1 | 0.5 | 1.7 | 17.6 | 4 | 6.4 | 1.7 | 7.4 | 6.8 | 1.6 | 9.3 | 10.3 | 11.8 | 11.9 | 8 | 2.1 | 1.5 | 1.4 | 1.5 | 1.3 | 1.4 | 1.3 | 1.4 | 1.9 | 1.6 |
| N2 | 0.5 | 1.9 | 16.3 | 3.6 | 6.6 | 1.9 | 7.8 | 5.6 | 2.3 | 9.6 | 9.2 | 10.3 | 10.1 | 7.8 | 2.2 | 1.6 | 1.6 | 1.9 | 2 | 1.7 | 2.3 | 1.9 | 1.5 | 1.8 |
| N3 | 0.7 | 2.2 | 20.9 | 2.8 | 5.5 | 2.6 | 10.1 | 7.2 | 2.1 | 9.5 | 8.7 | 10.4 | 9 | 7.3 | 3 | 2 | 2.3 | 2.2 | 2.3 | 2.2 | 1.8 | 1.9 | 2.4 | 2 |
| N4 | 0.4 | 1.1 | 10.6 | 5.7 | 7.2 | 1.5 | 8.2 | 5.2 | 1.4 | 7.4 | 6.8 | 10.1 | 7.1 | 6.2 | 1 | 1.5 | 1.6 | 1.1 | 1.2 | 1.2 | 1 | 1 | 1.2 | 1.1 |
| N5 | 0.3 | 2.1 | 10.4 | 3 | 6.4 | 1.5 | 6.9 | 4.6 | 1.8 | 7.7 | 7.5 | 9.2 | 8.7 | 6.2 | 2 | 1.8 | 1.6 | 1.8 | 1.7 | 2 | 1.9 | 1.7 | 2 | 2 |
| N6 | 0.3 | 0.3 | 5.5 | 0.9 | 1.8 | 0.5 | 1.7 | 1.8 | 0.5 | 2.4 | 2.6 | 3 | 2.2 | 2 | 0.5 | - | - | - | - | - | 0.4 | - | - | - |
| N7 | 0.4 | 0.6 | 5.4 | 1.1 | 2.8 | 0.9 | 5.6 | 5.8 | 0.7 | 8.8 | 8.4 | 8.9 | 9.1 | 4.3 | 0.8 | - | - | - | - | - | 0.9 | - | - | - |
| N8 | 0.6 | 2.3 | 14.5 | 2.9 | 8.9 | 2.1 | 13.3 | 7.4 | 2.2 | 13.9 | 11.6 | 13.1 | 10.6 | 8.9 | 3.1 | - | - | - | - | - | 2.7 | - | - | - |
| N9 | 0.3 | 1 | 14.4 | 1.3 | 6.4 | 1.2 | 8.4 | 0.9 | 1.1 | 8.3 | 8.9 | 29 | 8.2 | 7.4 | 1.3 | - | - | - | - | - | 1.3 | - | - | - |
| N10 | 0.3 | 1.1 | 9.5 | 1.6 | 7.5 | 1.1 | 6.7 | 9.1 | 1 | 6.6 | 6.9 | 9.1 | 8.4 | 7.8 | 1.2 | - | - | - | - | - | 1.2 | - | - | - |
|  |  |  |  |  |  |  |  |  |  |  |  |  |  |  |  |  |  |  |  |  |  |  |  |  |
|  |  |  |  |  |  |  |  |  |  |  |  |  |  |  |  |  |  |  |  |  |  |  |  |  |
| **CD56+ CD107a+** | FMO | K- | K+ (IL2+ PHA) | TLR3 | TLR7/8 | DOTAP | DOTAP+miR-122 | DOTAP+miR-192 | DOTAP+miR-193b | DOTAP+miR-185 | DOTAP+miR-195 | DOTAP+miR-455 | DOTAP+miR-375 | DOTAP+miR-129 | DOTAP+ RNA41 | miR-122 | miR-192 | miR-193b | miR-185 | miR-195 | miR-455 | miR-375 | miR-129 | RNA41 |
| P1 | 0.9 | 1.8 | #N/V | 3.2 | 9.8 | 2 | 6.4 | 5 | 2.4 | 6.5 | 11.3 | 13.9 | 8.9 | 6.8 | 2 | 2.6 | 2.7 | 2.3 | 2.3 | 2.9 | 2.7 | 2.3 | 2.5 | 2.1 |
| P2 | 0.9 | 3.7 | 24.8 | 3.4 | 9.4 | 3.4 | 7.7 | 6.8 | 2.6 | 8.4 | 9.1 | 15.7 | 8.7 | 7.5 | 2.4 | 2.6 | 2.1 | 1.5 | 1.9 | 1.6 | 3.3 | 3.5 | 2.8 | 2.2 |
| P3 | 0.9 | 6.2 | 12.6 | 6.6 | 16.1 | 6.1 | 17.5 | 11.7 | 5 | 16 | 17.4 | 24.2 | 18.2 | 13.3 | 6.9 | 5.2 | 5.4 | 5.5 | 4.6 | 6 | 7.4 | 5.5 | 5.2 | 5.2 |
| P4 | 0.5 | 2.4 | 16.3 | 5.1 | 15.2 | 2.6 | 12.5 | 9.1 | 2.5 | 17.6 | 19.3 | 27.9 | 18.7 | 12.8 | 2.1 | 1.7 | 2 | 2.8 | 2.8 | 1.9 | 1 | 2 | 1.9 | 3.6 |
| P5 | 1.1 | 4.7 | 34.6 | 8.5 | 24.8 | 5.4 | 36.4 | 26.2 | 5.6 | 35.4 | 39 | 39.5 | 32.4 | 18.1 | 4.6 | 5.9 | 5.2 | 4.5 | 3.6 | 6.5 | 3.8 | 3.7 | 4.5 | 5.4 |
| P6 | 0.9 | 3.6 | 14.8 | 8.4 | 13.9 | 4.2 | 16.8 | 16.3 | 3.8 | 16.6 | 17.6 | 19.9 | 23.5 | 18.8 | 4.5 | 3.7 | 3.1 | 4 | 3.9 | 3.3 | 3.2 | 2.8 | 3.4 | 4.4 |
| P7 | 0.9 | 6.3 | 16.5 | 5.4 | 10.6 | 6.4 | 13.9 | 4.8 | 8.9 | 12.2 | 13.2 | 11.1 | 19.5 | 10.6 | 5.3 | - | - | - | - | - | 3.9 | - | - | - |
| P8 | 0.6 | 1.9 | 10.9 | 5.4 | 15.2 | 2 | 8.1 | 9.3 | 2.6 | 12.3 | 14.1 | 16.1 | 8.7 | 10 | 2.4 | - | - | - | - | - | 2.1 | - | - | - |
| P9 | 0.7 | 6.9 | 18.4 | 8.6 | 20.6 | 5.1 | 21.1 | 18.6 | 6.9 | 27.7 | 19.9 | 36.6 | 22.3 | 21.4 | 9.4 | - | - | - | - | - | 7.5 | - | - | - |
| P10 | 0.5 | 3.5 | 11.4 | 6.3 | 12.1 | 2.3 | 10.2 | 8.6 | 2.2 | 11.3 | 11.1 | 15.7 | 10.1 | 8.2 | 3.6 | - | - | - | - | - | 4.2 | - | - | - |
| N1 | 0.8 | 3.8 | 19.3 | 7.1 | 11.6 | 3.5 | 9.8 | 11.6 | 3.8 | 14.4 | 16 | 19.3 | 17.8 | 11.9 | 4.4 | 3.5 | 4 | 3.9 | 3.5 | 3.1 | 3.1 | 3.1 | 5.1 | 4.3 |
| N2 | 1 | 4.1 | 21.1 | 8.1 | 14.2 | 4.2 | 13.9 | 10.8 | 4.8 | 14.7 | 15.6 | 17.2 | 17.4 | 12.1 | 4.9 | 3.9 | 3.2 | 4.9 | 4.4 | 5.4 | 3.7 | 4 | 4.7 | 5.6 |
| N3 | 1 | 2.4 | 19.7 | 4.2 | 13.1 | 3.8 | 20.5 | 12.6 | 3.3 | 17.3 | 16 | 20.9 | 15.7 | 12.9 | 4.3 | 3.4 | 4.2 | 3.9 | 3 | 4 | 3.7 | 3.8 | 6.3 | 4.6 |
| N4 | 0.8 | 3.5 | 19.7 | 13 | 21.5 | 5.1 | 16.9 | 11.4 | 4.1 | 17.9 | 16.1 | 27.1 | 19.9 | 15.8 | 4 | 5.9 | 6.5 | 5.4 | 5.5 | 5.2 | 4.8 | 4.9 | 4.9 | 3.4 |
| N5 | 0.5 | 3.7 | 11.8 | 6.6 | 12.5 | 3.7 | 12 | 6.4 | 2.8 | 16 | 14.5 | 18.4 | 16.3 | 11.4 | 3.7 | 3.7 | 3.2 | 3.7 | 4.2 | 3.8 | 3.3 | 4.1 | 3.6 | 3.9 |
| N6 | 0.8 | 1.5 | 7.9 | 1.6 | 5.2 | 1.3 | 4.1 | 3.7 | 1.1 | 4.6 | 4.8 | 5.1 | 5.3 | 4.3 | 1.4 | - | - | - | - | - | 2.5 | - | - | - |
| N7 | 0.5 | 2.1 | 6.6 | 1.9 | 4.8 | 1.5 | 6 | 6.5 | 1.4 | 10.4 | 11.2 | 12 | 10.6 | 5 | 2.1 | - | - | - | - | - | 1.4 | - | - | - |
| N8 | 0.2 | 3.8 | 16 | 4.8 | 17.4 | 3.4 | 27.8 | 10.6 | 2.7 | 27.2 | 17.3 | 32.2 | 23.1 | 16.9 | 5.3 | - | - | - | - | - | 3.3 | - | - | - |
| N9 | 0.5 | 3.4 | 16.2 | 3.3 | 12.5 | 3.3 | 15 | 2.8 | 3.2 | 11.1 | 12.7 | 38.4 | 13.2 | 10 | 3 | - | - | - | - | - | 3.7 | - | - | - |
| N10 | 0.6 | 4 | 13 | 3.8 | 13.5 | 4.4 | 8.3 | 14.8 | 3.5 | 9.4 | 10.9 | 17 | 12.8 | 13.8 | 4.6 | - | - | - | - | - | 4.9 | - | - | - |

Source data (Figures 3, 4; Tables 5 B, 6 B).: CD56+ NK-cell and CD8+ T-cell CD107a+ expression flow cytometry results 21h after the stimulation with DOTAP/miRNA.

Gating strategy: Vesicle/miRNA complex accumulation in lymphocytes, granulocytes, and monocytes for non-labeled hsa-miR-375-3p 2h after the stimulation with DOTAP/miRNA vesicle complex.


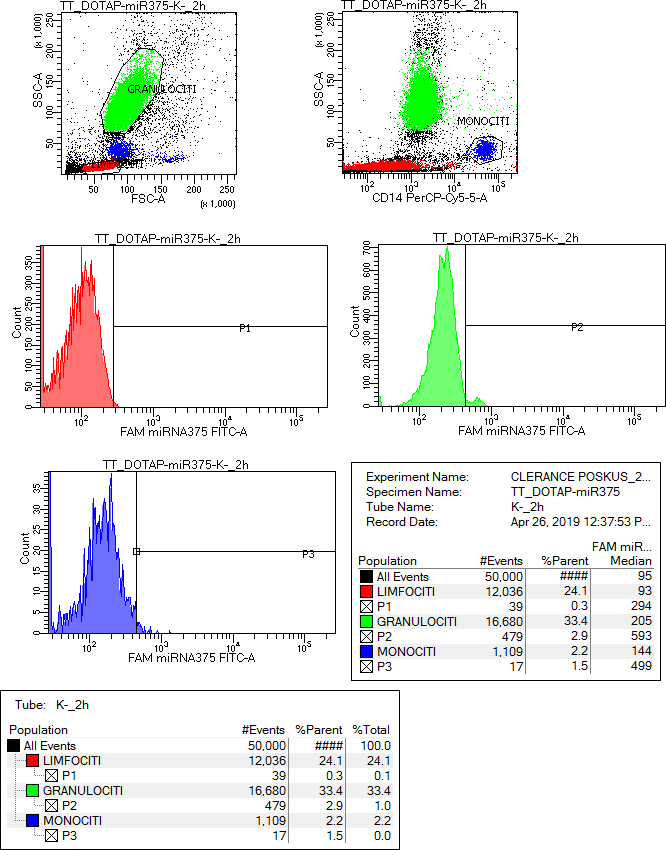


GRANULOCYTES

MONOCYTES

LYMPHOCYTES

LYMPHOCYTES

GRANULOCYTES

MONOCYTES

Gating strategy: Vesicle/miRNA complex accumulation in lymphocytes, granulocytes and monocytes, for FAM-labeled hsa-miR-375-3p -FAM 2h after the stimulation with DOTAP/miRNA vesicle complex.


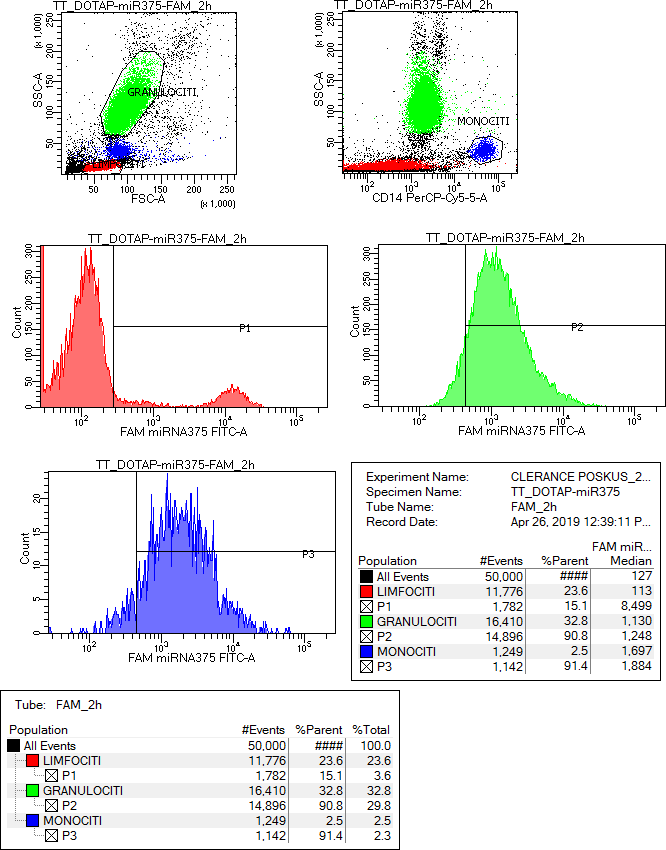


LYMPHOCYTES

GRANULOCYTES

MONOCYTES

LYMPHOCYTES

GRANULOCYTES

MONOCYTES

Source data Figure 5 D: Flow cytometry DOTAP/hsa-miR-375-3p -FAM / DOTAP/ hsa-miR-375‑3p ratio of vesicle accumulation in lymphocytes, granulocytes, and monocytes.

| Participant | DOTAP/miR  incubation time | Lymphocytes | Granulocytes | Monocytes |
| --- | --- | --- | --- | --- |
|  | 5min | 1.13 | 3.98 | 6.05 |
|  | 30min | 1.21 | 4.86 | 7.02 |
|  | 1h | 1.18 | 5.45 | 9.79 |
| A | 2h | 1.22 | 5.51 | 11.78 |
|  | 4h | 1.23 | 6.30 | 24.21 |
|  | 6h | 1.20 | 8.42 | 26.84 |
|  | 5min | 0.87 | 1.11 | 1.50 |
|  | 30min | 1.12 | 13.01 | 32.16 |
|  | 1h | 1.14 | 12.76 | 49.78 |
| B | 2h | 1.16 | 11.38 | 123.12 |
|  | 4h | 1.22 | 12.10 | 115.84 |
|  | 6h | 1.21 | 12.23 | 74.40 |
|  | 5min | 1.07 | 7.52 | 7.65 |
|  | 30min | 1.20 | 8.33 | 15.55 |
|  | 1h | 1.23 | 6.90 | 22.77 |
| C | 2h | 1.22 | 5.03 | 33.01 |
|  | 4h | 1.14 | 4.61 | 34.40 |
|  | 6h | 1.16 | 4.98 | 18.03 |

Source data Supplementary Figure 2: Chloroquine concentration (CQ) inhibitory response on DOTAP/miRNA stimulation (21h after the stimulation).

| miR-sample | CQ [µM] | CD4+CD69+ | CD8+CD69+ | CD8+CD107a+ | CD56+CD107a+ |
| --- | --- | --- | --- | --- | --- |
| K- | - | 1.2 | 1.8 | 1.2 | 1.8 |
| DOTAP/miR-455 | 0 | 6.2 | 23 | 2.7 | 6.1 |
| DOTAP/miR-455 | 10 | 1 | 9.2 | 1.2 | 3.7 |
| DOTAP/miR-455 | 20 | 0.9 | 3.1 | 1 | 3.7 |
| DOTAP/miR-455 | 30 | 1.2 | 1.9 | 0.9 | 4 |
| DOTAP/miR-455 | 40 | 0.9 | 2.3 | 1.2 | 5.8 |
| DOTAP/miR-455 | 50 | 1 | 1.7 | 1.4 | 6.8 |
| ssRNA40/LyoVec | 0 | 4.8 | 18.6 | 2.3 | 3.8 |
| ssRNA40/LyoVec | 10 | 0.5 | 2 | 0.4 | 2.3 |
| ssRNA40/LyoVec | 20 | 1.3 | 2.1 | 0.6 | 3.8 |
| ssRNA40/LyoVec | 30 | 1.6 | 1.6 | 1.1 | 4.2 |
| ssRNA40/LyoVec | 40 | 1.5 | 2.5 | 0.8 | 5.4 |
| ssRNA40/LyoVec | 50 | 1.2 | 3.2 | 1.1 | 7.4 |

Source data Figure 7: Chloroquine (CQ) inhibition of DOTAP/miRNA CD69 and CD107a activation 21h after the stimulation. In the table miRNA with synthetic DOTAP vesicles with the addition or without of CQ are listed. [K-: negative control (only blood cells); DOTAP-miR: miRNA with DOTAP synthetic vesicles. N=6].

| CD4+CD69+ | K- | DOTAP-miR-122 | DOTAP-miR-129 | DOTAP-miR-375 | DOTAP-miR-455 | K-_CQ | DOTAP-miR-122_CQ | DOTAP-miR-129_CQ | DOTAP-miR-375_CQ | DOTAP-miR-455_CQ |
| --- | --- | --- | --- | --- | --- | --- | --- | --- | --- | --- |
| A | 1.6 | 41.3 | 32 | 45.8 | 47.3 | 1.7 | 2.5 | 2.4 | 2.4 | 4 |
| B | 2.1 | 51.7 | 48.1 | 54.9 | 51.2 | 3.2 | 3.2 | 2.2 | 2.4 | 1.4 |
| C | 2.3 | 20.1 | 16.3 | 23.1 | 18.4 | 3.1 | 3.3 | 3.9 | 3.4 | 3.2 |
| D | 1.1 | 20.1 | 14.5 | 26.4 | 29.9 | 1.1 | 1 | 1.2 | 1.1 | 1.9 |
| E | 0.9 | 13.4 | 12.8 | 24.3 | 22 | 1.6 | 1.5 | 1.6 | 1.4 | 1.2 |
| F | 2.8 | 25.9 | 17.7 | 14.5 | 27.8 | 3 | 4 | 4.3 | 5.4 | 6 |
|  |  |  |  |  |  |  |  |  |  |  |
| CD4+CD69+ | K- | DOTAP-miR-122 | DOTAP-miR-129 | DOTAP-miR-375 | DOTAP-miR-455 | K-_CQ | DOTAP-miR-122_CQ | DOTAP-miR-129_CQ | DOTAP-miR-375_CQ | DOTAP-miR-455_CQ |
| A | 2.6 | 56.4 | 46.2 | 60.1 | 62.6 | 2 | 3.7 | 6.2 | 5.2 | 8.6 |
| B | 4.2 | 62.4 | 63.2 | 68.9 | 66.2 | 6.2 | 18.1 | 12.3 | 18.1 | 14.3 |
| C | 4.2 | 40.6 | 40.9 | 47.8 | 39.5 | 4.3 | 4.8 | 3.7 | 4.9 | 5.5 |
| D | 2.2 | 25.5 | 18.8 | 30.4 | 35.9 | 1.5 | 2.7 | 2.8 | 3.1 | 4 |
| E | 1.3 | 34.1 | 33 | 43.9 | 41.8 | 1.9 | 2.1 | 4.1 | 3.4 | 4.3 |
| F | 4.7 | 40.9 | 34.4 | 28.5 | 42.3 | 5.6 | 11.8 | 11.8 | 15.1 | 17.1 |
|  |  |  |  |  |  |  |  |  |  |  |
| CD8+CD107a+ | K- | DOTAP-miR-122 | DOTAP-miR-129 | DOTAP-miR-375 | DOTAP-miR-455 | K-_CQ | DOTAP-miR-122_CQ | DOTAP-miR-129_CQ | DOTAP-miR-375_CQ | DOTAP-miR-455_CQ |
| A | 0.5 | 5.3 | 3.9 | 7.1 | 6.8 | 0.6 | 0.9 | 1.1 | 1.5 | 0.9 |
| B | 2.5 | 18.8 | 17.8 | 19.3 | 19.7 | 2.8 | 4.1 | 3.7 | 3.9 | 4.2 |
| C | 3 | 8.8 | 6 | 7.8 | 9.2 | 2.7 | 2.8 | 4.1 | 2.7 | 3.5 |
| D | 2.9 | 8.5 | 7.1 | 10.6 | 8.9 | 5 | 5.2 | 4.9 | 4.1 | 3.6 |
| E | 2.7 | 5.5 | 4.6 | 9.1 | 7.2 | 3.3 | 4.4 | 4.4 | 4.7 | 4.2 |
| F | 3.9 | 10.9 | 11.8 | 5.2 | 10.9 | 4.2 | 4.8 | 4.4 | 6.3 | 6.1 |
|  |  |  |  |  |  |  |  |  |  |  |
| CD56+CD107a+ | K- | DOTAP-miR-122 | DOTAP-miR-129 | DOTAP-miR-375 | DOTAP-miR-455 | K-_CQ | DOTAP-miR-122_CQ | DOTAP-miR-129_CQ | DOTAP-miR-375_CQ | DOTAP-miR-455_CQ |
| A | 0.5 | 5.3 | 3.9 | 7.1 | 6.8 | 0.6 | 0.9 | 1.1 | 1.5 | 0.9 |
| B | 2.5 | 18.8 | 17.8 | 19.3 | 19.7 | 2.8 | 4.1 | 3.7 | 3.9 | 4.2 |
| C | 3 | 8.8 | 6 | 7.8 | 9.2 | 2.7 | 2.8 | 4.1 | 2.7 | 3.5 |
| D | 2.9 | 8.5 | 7.1 | 10.6 | 8.9 | 5 | 5.2 | 4.9 | 4.1 | 3.6 |
| E | 2.7 | 5.5 | 4.6 | 9.1 | 7.2 | 3.3 | 4.4 | 4.4 | 4.7 | 4.2 |
| F | 3.9 | 10.9 | 11.8 | 5.2 | 10.9 | 4.2 | 4.8 | 4.4 | 6.3 | 6.1 |

Source data Figure 7 (statistics): Chloroquine (CQ) inhibition of DOTAP/miRNA CD69 and CD107a expression 21h after the DOTAP/miRNA transfection (paired mean difference analysis). Hedges' g correction with 5000 resamplings and 95% confidence interval was used to predict the effect of miRNA stimulation. Stimulation results with FDR q-value < 0.05 were considered as significant [DOTAP: DOTAP synthetic vesicles, DOTAP_miRNA: miRNA transfected with DOTAP synthetic vesicles; CQ: Chloroquine; K-: samples with only blood cells; N=3].

| **CD4+CD69+** | control | test | difference | statistic paired students_t | P value | q value | Discovery? |
| --- | --- | --- | --- | --- | --- | --- | --- |
| 0 | K- | K-_CQ | 0.536589404 | -2.662904879 | 0.044728 | **0.012808** | Yes |
| 1 | DOTAP_miR-122 | DOTAP_miR-122_CQ | -2.318884008 | 4.492408378 | 0.006444 | **0.003722** | Yes |
| 2 | DOTAP_miR-129 | DOTAP_miR-129_CQ | -1.968347781 | 3.666507376 | 0.014496 | **0.005074** | Yes |
| 3 | DOTAP_miR-375 | DOTAP_miR-375_CQ | -2.425679519 | 4.389724845 | 0.00709 | **0.003722** | Yes |
| 4 | DOTAP_miR-455 | DOTAP_miR-455_CQ | -2.863398617 | 5.316363026 | 0.00315 | **0.003722** | Yes |
|  |  |  |  |  |  |  |  |
|  |  |  |  |  |  |  |  |
| **CD8+CD69+** | control | test | difference | statistic paired students_t | P value | q value | Discovery? |
| 0 | K- | K-_CQ | 0.2031114 | -0.925345429 | 0.397251 | 0.128343 | No |
| 1 | DOTAP_miR-122 | DOTAP_miR-122_CQ | -3.100724999 | 8.174704834 | 0.000445 | **0.000623** | Yes |
| 2 | DOTAP_miR-129 | DOTAP_miR-129_CQ | -2.753224857 | 6.312726132 | 0.001469 | **0.000928** | Yes |
| 3 | DOTAP_miR-375 | DOTAP_miR-375_CQ | -2.892524448 | 6.058741333 | 0.001767 | **0.000928** | Yes |
| 4 | DOTAP_miR-455 | DOTAP_miR-455_CQ | -3.629760546 | 8.335952877 | 0.000406 | **0.000623** | Yes |
|  |  |  |  |  |  |  |  |
|  |  |  |  |  |  |  |  |
| **CD8+CD107a+** | control | test | difference | statistic paired students_t | P value | q value | Discovery? |
| 0 | K- | K-_CQ | 0.3580622 | -1.523935025 | 0.188031 | **0.045561** | Yes |
| 1 | DOTAP_miR-122 | DOTAP_miR-122_CQ | -1.483334312 | 3.105176014 | 0.026696 | **0.010512** | Yes |
| 2 | DOTAP_miR-129 | DOTAP_miR-129_CQ | -1.130902466 | 2.258996344 | 0.073444 | **0.019279** | Yes |
| 3 | DOTAP_miR-375 | DOTAP_miR-375_CQ | -1.489208293 | 2.744876149 | 0.040556 | **0.011614** | Yes |
| 4 | DOTAP_miR-455 | DOTAP_miR-455_CQ | -1.729722103 | 3.700584523 | 0.013991 | **0.009018** | Yes |
|  |  |  |  |  |  |  |  |
|  |  |  |  |  |  |  |  |
| **CD56+CD107a+** | control | test | difference | statistic paired students_t | P value | q value | Discovery? |
| 0 | K- | K-_CQ | 1.912659674 | -3.69468309 | 0.014077 | **0.006569** | Yes |
| 1 | DOTAP_miR-122 | DOTAP_miR-122_CQ | -0.937956539 | 1.346325421 | 0.236009 | 0.095577 | No |
| 2 | DOTAP_miR-129 | DOTAP_miR-129_CQ | -0.609309395 | 0.961920628 | 0.380268 | 0.122856 | No |
| 3 | DOTAP_miR-375 | DOTAP_miR-375_CQ | -0.592882456 | 1.163558362 | 0.297105 | 0.103987 | No |
| 4 | DOTAP_miR-455 | DOTAP_miR-455_CQ | -0.798443384 | 1.29993806 | 0.25032 | 0.095577 | No |

Source data Figure 8A: DOTAP/ miRNA stimulation cytokine/chemokine release. DOTAP/miRNA immune system activation was evaluated with the assessment of cytokine and chemokine inflammatory profiles after 21h overnight incubation (37°C, 5% CO2 using LEGENDplex Human Inflammation Panel multi-analyte flow assay kit. [DOTAP: DOTAP synthetic vesicles, DOTAP_miRNA: miRNA transfected with DOTAP synthetic vesicles; CQ: Chloroquine; K-: samples with only blood cells; N=3].

| **Sample** | **Transfection** | IL-1beta | IFN-alpha2 | IFN-gamma | TNF-alpha | MCP-1 (CCL-2) | IL-6 | IL-8 | IL-10 | IL-12p70 | IL-17A | IL-18 | IL-23 | IL-33 |
| --- | --- | --- | --- | --- | --- | --- | --- | --- | --- | --- | --- | --- | --- | --- |
| S1 | DOTAP-122 | 180.63 | 218.01 | 205.84 | 273.58 | 12,189.00 | 1,033.00 | 648.47 | 61.48 | 42.83 | 38.20 | 23.40 | 26.41 | 26.41 |
| S2 | DOTAP-122 | 159.38 | 127.31 | 170.05 | 148.89 | 15,601.00 | 522.02 | 469.47 | 52.54 | 42.83 | 38.20 | 24.96 | 79.50 | 79.50 |
| S3 | DOTAP-122 | 454.77 | 79.67 | 1,505.00 | 294.03 | 14,602.00 | 2,711.00 | 698.75 | 135.72 | 42.83 | 38.20 | 39.19 | 26.41 | 26.41 |
| S1 | DOTAP-122-CQ | 15.07 | 16.94 | 13.10 | 21.31 | 5,493.00 | 55.80 | 1,131.00 | 22.16 | 42.83 | 38.20 | 33.50 | 9.06 | 9.06 |
| S2 | DOTAP-122-CQ | 15.07 | 13.23 | 17.69 | 15.24 | 2,778.00 | 21.29 | 450.85 | 18.24 | 42.83 | 38.20 | 15.06 | 40.97 | 40.97 |
| S3 | DOTAP-122-CQ | 34.23 | 13.23 | 17.69 | 22.97 | 11,274.00 | 263.99 | 1,249.00 | 31.27 | 42.83 | 38.20 | 39.19 | 15.09 | 15.09 |
| S1 | DOTAP-129 | 1,123.00 | 24.97 | 2,492.00 | 466.75 | 14,211.00 | 2,212.00 | 2,052.00 | 107.23 | 42.83 | 38.20 | 43.20 | 15.09 | 15.09 |
| S2 | DOTAP-129 | 710.10 | 18.88 | 1,977.00 | 352.17 | 20,367.00 | 1,776.00 | 2,176.00 | 132.99 | 42.83 | 38.20 | 24.96 | 79.50 | 79.50 |
| S3 | DOTAP-129 | 2,328.00 | 16.94 | 8,432.00 | 1,053.00 | 16,167.00 | 4,760.00 | 2,473.00 | 195.61 | 42.83 | 38.20 | 39.19 | 15.09 | 15.09 |
| S1 | DOTAP-129-CQ | 21.91 | 13.23 | 13.10 | 21.31 | 6,021.00 | 48.81 | 1,201.00 | 21.58 | 42.83 | 38.20 | 35.35 | 26.41 | 26.41 |
| S2 | DOTAP-129-CQ | 76.75 | 20.87 | 79.03 | 26.44 | 392.37 | 42.18 | 888.32 | 24.50 | 42.83 | 237.68 | 63.41 | 79.50 | 79.50 |
| S3 | DOTAP-129-CQ | 48.70 | 22.90 | 35.12 | 24.68 | 649.71 | 46.56 | 446.21 | 18.78 | 42.83 | 121.94 | 104.17 | 79.50 | 79.50 |
| S1 | DOTAP-193b | 12.00 | 14.14 | 17.69 | 13.86 | 165.79 | 12.50 | 158.60 | 13.56 | 42.83 | 38.20 | 29.94 | 15.09 | 15.09 |
| S2 | DOTAP-193b | 21.91 | 15.99 | 25.75 | 15.24 | 978.00 | 46.56 | 1,776.00 | 21.02 | 42.83 | 38.20 | 31.70 | 15.09 | 15.09 |
| S3 | DOTAP-193b | 15.07 | 15.06 | 15.31 | 18.16 | 187.40 | 22.95 | 509.55 | 16.11 | 42.83 | 38.20 | 37.25 | 26.41 | 26.41 |
| S1 | DOTAP-193b-CQ | 29.86 | 15.06 | 20.22 | 16.67 | 204.80 | 18.14 | 149.33 | 15.59 | 42.83 | 38.20 | 37.25 | 9.06 | 9.06 |
| S2 | DOTAP-193b-CQ | 21.91 | 18.88 | 11.19 | 15.24 | 331.85 | 19.69 | 173.69 | 18.78 | 42.83 | 38.20 | 28.23 | 26.41 | 26.41 |
| S3 | DOTAP-193b-CQ | 18.34 | 16.94 | 15.31 | 19.71 | 155.83 | 21.29 | 228.63 | 15.07 | 42.83 | 38.20 | 41.17 | 40.97 | 40.97 |
| S1 | DOTAP-375 | 5,958.00 | 831.74 | 6,726.00 | 2,242.00 | 15,437.00 | 8,267.00 | 1,797.00 | 828.13 | 63.46 | 223.85 | 145.75 | 26.41 | 26.41 |
| S2 | DOTAP-375 | 1,657.04 | 213.75 | 3,924.00 | 1,347.93 | 11,628.00 | 1,998.00 | 855.49 | 318.01 | 42.83 | 38.20 | 24.96 | 58.70 | 58.70 |
| S3 | DOTAP-375 | 5,224.00 | 82.24 | 9,207.00 | 2,109.00 | 8,702.00 | 4,988.00 | 872.40 | 865.63 | 42.83 | 42.07 | 73.46 | 40.97 | 40.97 |
| S1 | DOTAP-375-CQ | 18.34 | 15.99 | 15.31 | 19.71 | 16,093.00 | 30.08 | 331.57 | 45.36 | 42.83 | 38.20 | 39.19 | 15.09 | 15.09 |
| S2 | DOTAP-375-CQ | 34.23 | 16.94 | 35.12 | 18.16 | 17,040.00 | 92.62 | 2,506.00 | 50.36 | 42.83 | 121.94 | 49.56 | 58.70 | 58.70 |
| S3 | DOTAP-375-CQ | 21.91 | 14.14 | 17.69 | 22.97 | 14,573.00 | 283.56 | 439.28 | 74.73 | 42.83 | 38.20 | 23.40 | 9.06 | 9.06 |
| S1 | DOTAP-455 | 4,379.00 | 128.66 | 19,187.00 | 1,396.00 | 8,768.00 | 4,065.00 | 1,015.00 | 293.28 | 42.83 | 38.20 | 58.63 | 15.09 | 15.09 |
| S2 | DOTAP-455 | 1,415.00 | 58.31 | 6,239.00 | 701.55 | 13,661.00 | 3,173.00 | 716.52 | 363.01 | 42.83 | 38.20 | 29.94 | 26.41 | 26.41 |
| S3 | DOTAP-455 | 7,721.00 | 49.83 | 31,306.00 | 3,277.00 | 8,961.00 | 8,715.00 | 1,059.00 | 570.85 | 76.75 | 38.20 | 65.86 | 40.97 | 40.97 |
| S1 | DOTAP-455-CQ | 29.86 | 14.14 | 20.22 | 21.31 | 9,579.00 | 86.98 | 909.64 | 89.39 | 42.83 | 38.20 | 37.25 | 26.41 | 26.41 |
| S2 | DOTAP-455-CQ | 25.75 | 17.90 | 28.73 | 19.71 | 21,387.00 | 178.94 | 2,731.00 | 63.78 | 42.83 | 38.20 | 47.40 | 40.97 | 40.97 |
| S3 | DOTAP-455-CQ | 48.70 | 14.14 | 20.22 | 22.97 | 15,683.00 | 433.36 | 650.97 | 87.73 | 42.83 | 38.20 | 28.23 | 9.06 | 9.06 |
| S1 | DOTAP | 4.84 | 5.65 | 6.12 | 6.67 | 179.48 | 5.00 | 179.41 | 6.03 | 17.13 | 15.28 | 23.45 | 3.62 | 3.62 |
| S2 | DOTAP | 7.34 | 4.59 | 6.12 | 5.54 | 80.38 | 5.00 | 173.87 | 5.82 | 17.13 | 15.28 | 10.63 | 23.48 | 23.48 |
| S3 | DOTAP | 7.34 | 6.40 | 9.16 | 6.09 | 135.49 | 9.86 | 297.85 | 6.65 | 17.13 | 15.28 | 37.00 | 10.57 | 10.57 |
| S1 | DOTAP-CQ | 6.03 | 5.65 | 9.16 | 8.53 | 289.44 | 6.08 | 173.87 | 6.03 | 17.13 | 16.83 | 28.35 | 16.39 | 16.39 |
| S2 | DOTAP-CQ | 6.03 | 6.78 | 8.09 | 6.67 | 389.64 | 7.88 | 279.50 | 6.86 | 17.13 | 15.28 | 12.68 | 10.57 | 10.57 |
| S3 | DOTAP-CQ | 4.84 | 6.02 | 5.24 | 6.09 | 163.48 | 5.00 | 102.06 | 5.82 | 17.13 | 15.28 | 16.47 | 10.57 | 10.57 |
| **S1** | NTC-10X | 7.34 | 6.02 | 8.09 | 7.88 | 546.35 | 9.86 | 381.03 | 6.23 | 17.13 | 15.28 | 41.67 | 16.39 | 16.39 |
| **S2** | NTC-10x | 11.95 | 6.40 | 6.12 | 8.53 | 109.46 | 10.57 | 295.80 | 6.03 | 17.13 | 15.28 | 14.90 | 6.03 | 6.03 |
| **S3** | NTC-10x | 7.34 | 6.02 | 5.24 | 7.27 | 64.73 | 9.18 | 92.26 | 5.82 | 17.13 | 15.28 | 28.35 | 10.57 | 10.57 |
| **S1** | NTC-10X-CQ | 13.69 | 8.35 | 14.05 | 10.58 | 507.22 | 17.74 | 1,121.00 | 9.09 | 17.13 | 63.25 | 129.93 | 41.31 | 41.31 |
| **S2** | NTC-10X-CQ | 8.76 | 5.29 | 8.09 | 7.88 | 366.13 | 6.66 | 475.93 | 6.23 | 17.13 | 15.28 | 16.47 | 6.03 | 6.03 |
| **S3** | NTC-10x-CQ | 7.34 | 5.65 | 4.47 | 6.67 | 351.18 | 9.18 | 492.91 | 6.03 | 17.13 | 15.28 | 33.65 | 3.62 | 3.62 |
| **S1** | ssRNA-40 | 13,518.00 | 19.87 | 30,031.00 | 4,584.00 | 7,967.00 | 6,933.00 | 2,595.00 | 274.20 | 80.21 | 38.20 | 101.20 | 40.97 | 40.97 |
| **S2** | ssRNA-40 | 10,069.00 | 18.88 | 76,872.00 | 6,170.00 | 11,917.00 | 14,022.00 | 1,452.00 | 549.22 | 71.66 | 38.20 | 63.41 | 26.41 | 26.41 |
| **S3** | ssRNA-40 | 9,860.00 | 15.99 | 10,116.00 | 4,959.00 | 8,162.00 | 9,342.00 | 1,461.00 | 743.90 | 58.71 | 38.20 | 63.41 | 15.09 | 15.09 |
| **S1** | ssRNA-40-CQ | 12.10 | 14.14 | 15.31 | 13.86 | 535.98 | 21.29 | 280.17 | 15.59 | 42.83 | 38.20 | 45.28 | 15.09 | 15.09 |
| **S2** | ssRNA-40-CQ | 25.75 | 16.94 | 17.69 | 21.31 | 2,675.00 | 60.65 | 1,376.00 | 16.63 | 42.83 | 42.07 | 35.35 | 26.41 | 26.41 |
| **S3** | ssRNA-40-CQ | 21.91 | 15.06 | 13.10 | 21.31 | 2,613.00 | 26.41 | 885.66 | 15.59 | 42.83 | 38.20 | 33.50 | 15.09 | 15.09 |

**Small RNA Next-generation sequencing data analysis.**

Raw reads were trimmed for adaptor sequence (AGATCGGAAGAGCACACGTCTGAACTCCAGTCAC) using cutadapt v1.16 (min. sequence length 15, Q20, error rate in adaptor recognition 0.1).
Trimmed sequenced were further analyzed using sRNAtoolboxVM tools collection v1.3; sRNAbench genome mode was used to align trimmed reads on GRCh38 human reference and GRCh38 related RNA database collection of known RNA sequences included in sRNAtoolbox (miRBase, snRNA, snoRNA, tRNA, rRNA, ncRNA, cDNA, mRNA, piRNA, repeats).

sRNAde tool with edgeR was used for miRNAs differential expression analysis between compared groups of studied samples. Only miRNA with minimum ten raw reads of at least one group per nT1D-HC, 10yT1D-nT1D,10yT1D-HC and ten reads per million of at least one participant for Langerhans islet transplantation patients samples were analyzed for differential expression.
